# Evaluating Manual Therapy in Musculoskeletal Pain: Why Certain Trial Designs May Overestimate Effectiveness—A Scoping Review

**DOI:** 10.1002/ejp.70150

**Published:** 2025-11-13

**Authors:** Jean‐Pascal Grenier, Alex Thiel

**Affiliations:** ^1^ Department of Internal Medicine II Medical University of Innsbruck, University Clinic Innsbruck Innsbruck Austria; ^2^ Department of Physiotherapy Health University of Applied Sciences Tyearol, FH Gesundheit Tirol Innsbruck Austria; ^3^ Department of Health Sciences University of Applied Sciences, FH Campus Wien Vienna Austria

**Keywords:** manual therapy, methods, musculoskeletal, pain, scoping review, study design

## Abstract

**Background and Objective:**

Atraumatic musculoskeletal pain, regardless of the affected body region, is a highly prevalent condition impacting over 25% of the global population and contributing significantly to the burden of disease. A common study design compares physiotherapy or exercise therapy alone to the same intervention combined with MT (A vs. A + B). This study design is inherently flawed due to its inability to isolate the effect of treatment B, the potential for interaction effects, and the lack of control for non‐specific contextual factors. The goal of this study was to compile studies using that approach and to examine the short‐, medium‐, and long‐term effects of the addition of MT to a control treatment.

**Databases and Data Treatment:**

This scoping review identified 95 randomised controlled trials (RCTs) with a systematic literature search in the electronic bibliographic databases MEDLINE (via PubMed), EBSCO, and PEDro.

**Results:**

Long‐term effects were absent, and medium‐term effects were infrequent. Approximately half of the studies reported statistically significant effects in the immediate or short‐term follow‐up; however, these effects were of limited clinical relevance and susceptible to methodological issues. Furthermore, studies with lower methodological quality were more likely to report significant effects (85%), whereas medium‐ and high‐quality studies showed positive results in only 50% of cases.

**Conclusions:**

This review highlights significant research gaps and provides methodological insights. The study design in question is therefore methodologically problematic, as it tends to generate positive short‐term results without providing clear answers or meaningful clinical implications for researchers and clinicians.

**Significance Statement:**

This scoping review summarizes studies using an ‘A vs. A+B design’, where manual therapy is added to usual care, with or without a sham control. The review identifies a lack of medium‐ or long‐term effectiveness and highlights a methodological bias toward generating positive short‐ or immediate‐term results of questionable clinical relevance. Based on these findings, we provide several recommendations to improve future research and to support clinicians in interpreting the current evidence base.

## 1 Introduction and Background

Between 20% and 40% of people globally suffer from chronic pain (Cohen et al. 2021). Clinical practice guidelines recommend a multidisciplinary biopsychosocial approach for managing chronic pain, incorporating education, physical exercise, psychological therapies, and, in some cases, manual therapy (MT) and physical interventions (Oliveira et al. 2018). However, the optimal components of physiotherapy treatments remain unclear.

MT, a non‐pharmacological intervention frequently used by physiotherapists in patients with musculoskeletal pain, appears to work primarily through non‐specific effects—an explanation that has also been proposed by MT proponents (Bialosky et al. 2017). Compelling evidence demonstrates that MT is not superior to sham MT in musculoskeletal pain (Lavazza et al. 2021; Molina‐Álvarez et al. 2022) but that MT reduces pain intensity in the short term through neurophysiological and non‐specific contextual effects (Bialosky et al. 2009). Every medical and physiotherapeutic intervention is influenced by non‐specific effects; however, the goal is to go beyond these and offer treatments that have a specific therapeutic effect (McDevitt et al. 2023). MT is sometimes justified by studies comparing exercise therapy alone to exercise therapy combined with MT (Abbott et al. 2015; Michener et al. 2024). This ‘A versus A+B’ design has been criticised for its interpretative limitations and coined as a ‘study design that generates only positive results’ (Ernst and Lee 2008). In efficacy trials, one intervention is typically compared to a credible control to account for non‐specific effects, attention, time spent with clinicians, and face validity (Jaeschke et al. 1994; Tikkinen and Guyatt 2021). Adding an intervention to an established therapy introduces potential confounding factors, such as increased clinician‐patient interaction, altered expectations, and enhanced credibility (Ernst and Lee 2008; Rossettini et al. 2020). If such a trial yields positive results, it may simply reflect amplified non‐specific effects rather than the true efficacy of the added intervention (Cashin et al. 2021; Hafliðadóttir et al. 2021; Rossettini et al. 2018). This is particularly relevant for MT, where the mechanisms of action remain debated and appear rather non‐specific (Grenier and Rothmund 2024; Keter et al. 2025).

Despite these limitations, this study design can be valuable given its proximity to clinical practice and its pragmatic nature (Mintken et al. 2016). Understanding the magnitude of these effects—whether a specific incremental benefit of adding MT or a contextual non‐specific effect—may therefore be clinically relevant (Saueressig et al. 2023). Nonetheless, while this study design is clinically meaningful and potentially useful, it has been criticized for methodological challenges (Ernst and Lee 2008), and its value in providing reliable estimates of efficacy remains questionable.

Despite abundant research, no comprehensive review has summarised the evidence for adding MT to usual care, physiotherapy, or exercise therapy for non‐traumatic musculoskeletal pain. This scoping review aims to systematically compile studies that compare physiotherapy or exercise therapy alone with the same therapy combined with MT in a randomised controlled trial (RCT). Our working hypothesis posits that such studies may be biased toward short‐term positive effects, largely driven by non‐specific contextual factors.

## 2 Methods

This scoping review was reported according to the PRISMA Extension for Scoping Reviews (see Data S4 for PRISMAScR checklist) (Tricco et al. 2018). Our methodological approach was informed by the 5‐stage framework from Arksey and O'Malley (von Elm et al. 2019; Levac et al. 2010; Munn et al. 2018; Tricco et al. 2018). We published a study protocol for this scoping review (https://osf.io/z3vpd/) in April 2024. We adhered to the guidelines provided by the Joanna Briggs Institute for scoping reviews (Peters et al. 2015). The PCC framework (population, concept, and context) was used for guidance for clear objectives and eligibility criteria (see Table 1) as recommended for scoping reviews (Pollock et al. 2023). Given the heterogeneous patient populations and the research question, we conducted a scoping rather than a systematic review. We used the web‐based ‘Right Review’ tool to confirm this decision (Amog et al. 2022).

**TABLE 1:** Inclusion and exclusion criteria.

Inclusion criteria	Exclusion criteria
Adults (aged 18 years or older) in any healthcare setting or location experiencing non‐traumatic acute, subacute, or chronic musculoskeletal pain, regardless of the body region (including neck pain, low back pain, shoulder pain, knee pain, tendinopathy, osteoarthritis, etc.)	Healthy participants with no musculoskeletal pain
	Pain resulting from an underlying specific pathology (e.g., infection, malignancy, inflammatory condition, fracture, spinal stenosis, ankle sprain)
Manual therapy was defined as non‐thrust joint mobilisation techniques, joint thrust techniques (manipulation)	Manual therapy was defined as traditional Chinese manual therapy, exclusively soft tissue techniques (such as Tuina or China‐massage) or specifically osteopathic care
We checked for relevant randomised controlled trials in systematic reviews published in peer‐reviewed journals, of which eligible primary studies (RCTs) where identified and included if appropriate	Non‐research articles such as editorials, commentaries, non‐randomised controlled studies, conference abstracts, letters to the editor, study protocols, observational studies, and cohort studies
This review included studies that added manual therapy to usual care, physiotherapy, or exercise therapy, compared with no additional intervention, a sham intervention or an attention control	When manual therapy, along with one or more additional interventions, was added to the treatment package alongside usual care or as self‐administered manual therapy
Pain intensity or physical function was assessed as an outcome. Results were categorised into the duration of follow‐up into immediate term (< 1 month), short‐term (< 1–6 months), mid‐term (7–12 months), or long‐term (> 12 months) follow‐up	Pain intensity and physical function were not assessed as outcomes

Abbreviations: MT, manual therapy; RCTs, randomised controlled trials.

### 2.1 Population

We included studies focusing on adult patients (> 18 years old) with non‐traumatic musculoskeletal pain (e.g., LBP, osteoarthritis [OA], tendinopathy) of unspecified duration. Studies were excluded if they involved patients with specific pain disorders, such as fractures, infections, autoimmune diseases, cancer, cauda equina syndrome, or post‐traumatic rehabilitation.

### 2.2 Concept

We considered RCTs conducted in the specified patient population that compared usual care, physiotherapy, or exercise therapy, alongside a placebo or sham intervention, to the same therapy with the addition of MT. The primary outcomes of interest were pain intensity and physical function or disability. For studies with more than two treatment groups, we only analysed the groups comparing usual care to usual care plus MT. Systematic reviews were screened for eligible RCTs, and individual studies within those reviews were extracted to avoid duplication in this review.

### 2.3 Context

There were no restrictions on the healthcare setting in which studies were conducted (e.g., primary care, community, acute care, outpatient therapy).

### 2.4 Literature Search

We systematically searched the electronic bibliographic databases MEDLINE (via PubMed), EBSCO, and PEDro between March and May 2024. All sources were searched from inception to May 2024. Studies published in English, German, or French were eligible for inclusion. The search strategies were developed through iterative pilot searches and refined via team discussions. The final search strategies for PubMed, EBSCO, and PEDro are provided as Data S1. Additionally, the reference lists of included studies were reviewed to identify further eligible studies.

### 2.5 Study Selection and Data Extraction

Duplicate records were removed during the selection process. Two reviewers (JG, AT) independently screened titles and abstracts based on predefined inclusion and exclusion criteria, resolving disagreements through discussion. This process was repeated for full‐text screening. Study and participant characteristics were manually extracted into an Excel file (see Data S2).

Key study characteristics—including first author, year, location, study design, intervention and control groups, sample size, intervention duration and frequency, definition of MT, population, control intervention, and metrics such as adverse events—were charted by one reviewer and verified by the other. The primary outcomes of interest, pain intensity and physical function or disability, were categorized and extracted by time frame: immediate‐term (< 4 weeks), short‐term (1–6 months), mid‐term (7–12 months), and long‐term (> 12 months). A study was classified as positive if either pain intensity or disability showed a statistically significant difference compared to the control group at any follow‐up. This inclusive approach was adopted to ensure potential benefits were not overlooked.

### 2.6 Data Synthesis

A narrative and mapping synthesis of the results was conducted. Due to the variability in pain sites and patient populations, a scoping review methodology was deemed more appropriate than a systematic review. Studies that accounted for attention and contextual effects by including sham interventions or attention controls within the usual care group were also considered. To enhance clarity in synthesis and interpretation, statistically significant results were further evaluated for their clinical relevance.

Our analysis of clinical relevance relied on prior research indicating that a 20%–30% reduction in pain or improvement in disability represents the smallest worthwhile difference for patients with musculoskeletal pain, particularly LBP and neck pain (Christiansen et al. 2018; Ferreira et al. 2013; Fritsch et al. 2023; Hansford et al. 2024). However, since the smallest worthwhile effect did not differ across various musculoskeletal pain sites, we applied this threshold to all studies included in our review (Christiansen et al. 2018). A detailed description of this approach, informed by broader evidence on minimally clinically important differences (MCID) despite its limitations (Ferreira et al. 2012), is provided in the Supporting Information.

### 2.7 Study Quality Assessment

While not initially planned or methodologically required for a scoping review (Peters et al. 2015), as outlined in our scoping review protocol, we decided during the review process to assess study quality due to the generally low quality observed in many studies. We employed the validated critical appraisal tool from the Joanna Briggs Institute for RCTs (Barker et al. 2023). This recently revised checklist includes 13 items to evaluate the risk of bias in RCTs (scored as yes = 1 point, no = 0 points, n/a = not applicable, or unclear). While no formal thresholds exist to categorise studies into low, moderate, or high quality, higher scores naturally reflect greater methodological quality and a lower risk of bias. We categorised studies into high quality (10–13 points), medium quality (7–9 points), and low quality (6 points and less). The study quality assessments were conducted independently by two authors (J.‐P.G., A.T.); conflicts were resolved through discussion. The individual assessments are included in the Supporting Information.

### 2.8 Deviations From the Study Protocol

During the review process, we decided that the most effective method to avoid double inclusion in systematic reviews was to extract individual studies from relevant reviews. This approach aligns with methodological recommendations for scoping reviews (Pollock et al. 2023). Initially, given that this was a scoping rather than a systematic review, we did not plan to perform a study quality assessment as outlined above. However, we integrated a formal risk of bias assessment for RCTs as described in Section 2.7 (Barker et al. 2023).

At first, we planned to exclude studies focusing solely on patients with radiculopathy due to differences in pain mechanisms compared to non‐specific musculoskeletal pain. We reconsidered this decision and broadened our inclusion criteria to encompass studies involving patients with radiculopathy. This decision was made because we aimed to examine the effectiveness of manual therapy in addition to usual care for reducing pain intensity in musculoskeletal conditions, with less emphasis on the specific pain mechanisms involved. We view this expansion as a strength rather than a limitation, as it provides a broader scope and demonstrates that MT is applicable to this population.

## 3 Results

**FIGURE 1:**
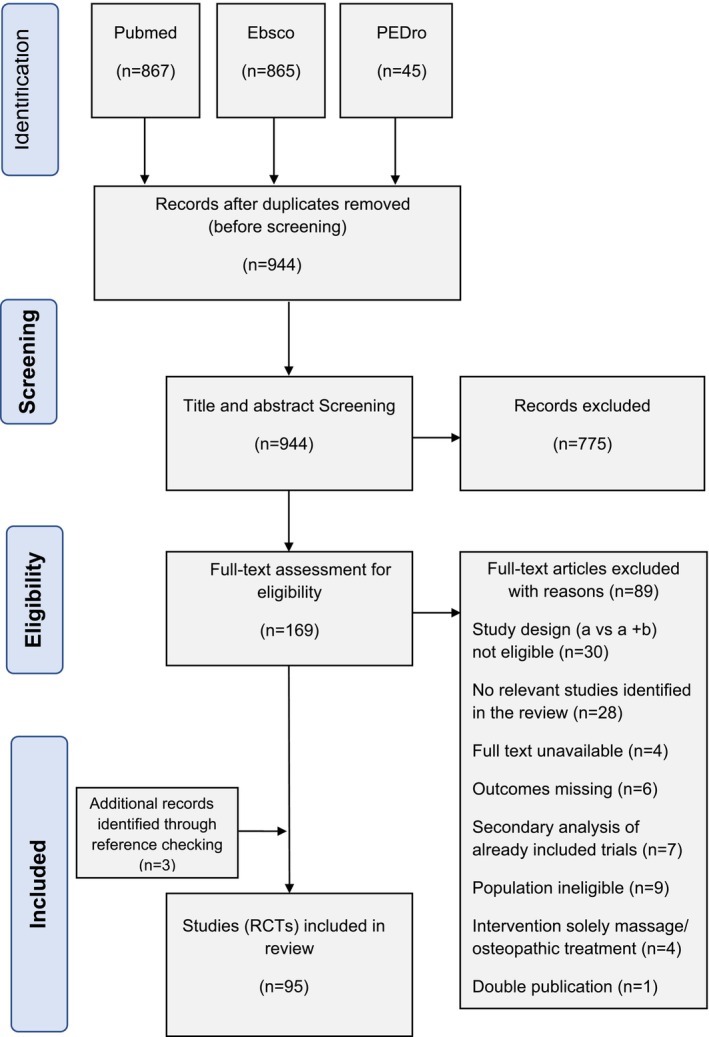
Flowchart of the literature search, according to PRISMA (Page et al. 2021).

A total of 95 studies were deemed eligible for this scoping review (see flowchart in Figure 1). Following the removal of duplicates, two authors independently screened 944 titles and abstracts. Of these, 775 records were excluded based on the predefined inclusion and exclusion criteria. This left 169 full‐text articles for further evaluation, of which 89 were excluded for not meeting the eligibility criteria (detailed reasons are provided in the Supporting Information).

Ultimately, 56 relevant RCTs and 24 systematic reviews were included. From these systematic reviews, an additional 36 eligible primary studies were identified. Additionally, reference‐checking of the included studies yielded three more RCTs. In total, 95 eligible RCTs were included in this scoping review.

### 3.1 Study Characteristics and Methodological Study Quality

In total, 95 RCTs investigated MT in addition to usual care, physiotherapy, or exercise therapy in patients with musculoskeletal pain. Most were conducted in the USA (*n* = 16), Turkey (*n* = 11), and India (*n* = 11). The most frequently examined pathologies included neck pain (*n* = 28), shoulder pain (*n* = 18), LBP (*n* = 14), and knee OA (*n* = 14). For a detailed summary of study descriptions and characteristics, please refer to Table 2.

**TABLE 2:** Study characteristics.

First author, year	Body region	Diagnosis	Sample size (% female)	Age (mean, SD)	Healthcare setting	Country	Treatment duration	Treatment frequency (total treatment sessions)
Abbott et al. 2013	Knee	Knee OA	206 (55.3)	66.0 (Range: 37–92)	Outpatient clinic	New Zealand	16 weeks	1/week for 7 weeks, two booster sessions in week 16 (9 sessions)
Abbott et al. 2015	Knee	Knee OA	75 (61.3)	61.0 (12.0)	Outpatient clinic	New Zealand	9 weeks	1–2/week over 9 weeks (12 sessions)
Akgüller et al. 2024	Neck	Chronic neck pain	60 (56.7)	31.7 (6.7)	Hospital	Turkey	6 weeks	2/week for 6 weeks (12 sessions)
Akhter et al. 2014	Neck	Chronic neck pain	61 (63.0)	38.1 (Range: 23–49)	Hospital	Pakistan	3 weeks	2/week for 3 weeks (6 sessions)
Al‐Banawi et al. 2023	Back	Subacute/chronic LBP	58 (100.0)	48.5 (8.1)	Outpatient clinic	Saudi Arabia	2 weeks	3/week for 3 weeks (9 sessions)
Ali and Khan 2015	Shoulder	Frozen shoulder	22 (?)	51.3 (?)	Hospital	Pakistan	5 weeks	3/week for 5 weeks (15 sessions)
Azlin and Lyn 2011	Knee	Knee OA	20 (15.3)	63.1 (10.8)	Outpatient clinic	Malaysia	4 weeks	2/week for 4 weeks (8 sessions)
Bakken et al. 2021	Neck	Chronic neck pain	131 (55.7)	57.0 (14.0)	Rehabilitation facility	Sweden	2 weeks	2/week for 2 weeks (4 sessions)
Bang and Deyle 2000	Shoulder	SPSS	52 (42.3)	43.0 (9.1)	Hospital	USA	3 weeks	2/week for 3 weeks (6 sessions)
Barbosa et al. 2008	Shoulder	SSP tendinopathy	14 (64.3)	46.1 (7.6)	Hospital	Brazil	4 weeks	3/week for 4 weeks (10 sessions)
Bergman et al. 2004	Shoulder	SPSS	150 (52.7)	48.4 (12.4)	Outpatient clinic	The Netherlands	12 weeks	0.5–1/week for 12 weeks (6 sessions)
Blackman and Atkins 2014	Hip	Hip OA	23 (78.2)	65.0 (10.1)	Outpatient clinic	UK	6 weeks	1/week (6 sessions), control group one session
Bolton et al. 2020	Foot	Ankle instability	30 (70.0)	23.6 (4.4)	Hospital	USA	6 weeks	2/week for 6 weeks (12 sessions)
Bronfort et al. 2001	Neck	Chronic neck pain	191 (59.2)	44.3 (10.6)	Not specified	USA	11 weeks	2–3/week for 11 weeks (20 sessions)
Bronfort et al. 2014	Back	Subacute/chronic LBP	192 (59.0–68.0)	57.1 (12.0)	Not specified	USA	12 weeks	1–3/week for 12 weeks (up to 20 sessions)
Camargo et al. 2015	Shoulder	SPSS	46 (47.8)	36.0 (12.1)	Not specified	Brazil	4 weeks	Unclear
Celenay et al. 2016	Neck	Neck pain	102 (72.5)	44.0 (13.0)	Hospital	Turkey	4 weeks	4 weeks 3 days/week (12 sessions)
Çelik 2016	Shoulder	Frozen shoulder	26 (69.2)	54.2 (7.9)	Hospital	Turkey	6 weeks	3/week for 6 weeks (18 sessions)
Ceylan et al. 2023	Hand	Carpal tunnel syndrome	45 (76.5)	45.9 (11.1)	Hospital	Turkey	4 weeks	3/week for 4 weeks (12 sessions)
Chen et al. 2009	Shoulder	SPSS	90 (71.1)	64.7 (12.5)	Hospital	Australia	8 weeks	1–2/week for 8 weeks (10 sessions)
Childs et al. 2004	Back	LBP	131 (42.0)	33.9 (10.9)	Not specified	USA	4 weeks	2/week first week and then 1/week for 3 weeks (5 sessions)
Conroy and Hayes 1998	Shoulder	SPSS	15 (46.7)	52.9 (?)	Not specified	USA	3 weeks for 3/week	3/week for 3 weeks (9 sessions)
Cook et al. 2014	Shoulder	SPSS	56 (45.6)	52.6 (14.1)	Outpatient clinic	USA	Patient discharge, treatment length, and frequency of treatment were determined by the physiotherapists, although some patients terminated treatment themselves.	
Copurgensli et al. 2016	Neck	Neck pain	45 (?)	49.9 (7.2)	Hospital	Turkey	3 weeks	5/week for 3 weeks (15 sessions)
Corum et al. 2018	TMJ	CMD	60 (100.0)	27.0 (6.3)	Hospital	Turkey	Unclear	6 sessions
Delgado et al. 2020	TMJ	CMD	61 (59.0)	42.5 (12.0)	Outpatient clinic	Spain	4 weeks	1–2/week (5 sessions)
Dogan et al. 2021	Back	SI‐joint pain	64 (67.2)	39.0 (11.3)	Not specified	Turkey	3 weeks	1/week for 3 weeks and 2/day home exercises (3 sessions)
Duymaz 2018	Neck	Neck pain	40 (87.0)	33.4 (6.1)	Hospital	Turkey	2 weeks	5/week for 2 weeks (10 sessions)
Dwyer et al. 2015	Knee	Knee OA	78 (62.8)	62.2 (11.8)	Outpatient clinic	USA/South Africa	4 weeks	3–4/week for 4 weeks (12 sessions)
Dziedzic et al. 2005	Neck	Neck pain	350 (63.1)	52.8 (Range: 24–83)	Outpatient clinic	UK	6 weeks	1–2/week for 6 weeks (8 sessions)
Eldesoky et al. 2019	Neck	Cervical radiculopathy	50 (46.0)	43.9 (4.9)	Hospital	Saudi Arabia	4 weeks	3/week for 4 weeks (12 sessions)
Eliason et al. 2021	Shoulder	SSPS	120 (49.2)	43.2 (9.8)	Outpatient clinic	Sweden	12 weeks	1–2/week for 12 weeks (20 sessions)
Espí‐López et al. 2020	TMJ	CMD	16 (81.0)	30.0 (11.5)	Not specified	Spain	4 weeks	1/week for 4 weeks (4 sessions)
Evans et al. 2012	Neck	Neck pain	270 (71.4)	44.1 (11.6)	Outpatient clinic	USA	12 weeks	1–2/week for 12 weeks (20 sessions)
Farooq et al. 2018	Neck	Neck pain	68 (45.5)	41.8 (10.9)	Outpatient clinic	Iran	4 weeks	2–3/week over 4 weeks (10 sessions)
Fathollahnejad et al. 2019	Neck	Neck pain	60 (100.0)	37.0 (3.1)	Not specified	Iran	6 weeks	3/week for 6 weeks (18 sessions)
Fitzgerald et al. 2016	Knee	Knee OA	300 (66.3)	58.0 (9.8)	Outpatient clinic	USA	9 weeks	1–2/week for 9 weeks (12 sessions)
French et al. 2013	Hip	Hip OA	131 (64.1)	61.4 (10.8)	Hospital	Ireland	8 weeks	1/week for 8 weeks (6–8×)
Ganesh et al. 2015	Neck	Neck pain	60 (36.7)	41.7 (9.8)	Hospital	India	2 weeks	2 weeks 5/week (10 sessions)
González‐Iglesias, Fernández‐de‐las‐Peñas, Cleland, Alburquerque‐Sendín, et al. 2009	Neck	Acute neck pain	45 (53.3)	34.0 (5.0)	Outpatient clinic	Spain	3 weeks	2/week for 3 weeks (6 sessions)
González‐Iglesias, Fernández‐de‐las‐Peñas, Cleland, and Del Gutiérrez‐Vega 2009	Neck	Neck pain	45 (46.7)	34.0 (4.0)	Not specified	Spain	3 weeks	1–2/week for 3 weeks (5 sessions)
González‐Rueda et al. 2021	Neck	Neck pain	78 (81.1)	60.0 (13.3)	Outpatient clinic	Spain	3 weeks	5/week for 3 weeks (15 sessions)
Grunnesjö et al. 2004	Back	LBP	160 (50.6)	41.6 (?)	Hospital	Sweden	Unclear	2.2–2.6 treatments in both groups (2–3 sessions)
Gutiérrez‐Espinoza et al. 2023	Shoulder	SSPS	72 (16.7)	45.2 (6.8)	Not specified	Brazil	6 weeks	6 weeks 2/week (12 sessions)
Haider et al. 2018	Shoulder	SSPS	45 (55.0)	49.5 (9.7)	Hospital	USA	2 weeks	2 weeks, 3/week (6 sessions)
Hallegraeff et al. 2009	Back	Acute LBP	64 (45.3)	40.0 (9.6)	Outpatient clinic	The Netherlands	2.5 weeks	1–2/week over 2.5 weeks (4 sessions)
Hancock et al. 2007	Back	Acute LBP	240 (44.0)	40.7 (15.6)	Outpatient clinic	Australia	4 weeks	2–3/week for 4 weeks (5 sessions)
Hoving et al. 2006	Neck	Neck pain	183 (?)	Unclear	Not specified	The Netherlands	6 weeks with MT 1/week and PT 2/week with a maximum of 6 MT sessions for 6 weeks and a maximum of 12 treatment sessions for 6 weeks PT (6–12 sessions)	
Javadov et al. 2021	Back	SI‐joint pain	69 (100.0)	36.3 (9.1)	Hospital	Turkey	3 weeks	1/week for 3 weeks, 2/day home exercise program
Joshi et al. 2014	Knee	Knee pain	30 (?)	Unclear	Not specified	India	3 weeks	5 days/week for 3 weeks (15 sessions)
Jull et al. 2002	Head	Cervicogenic headache	200 (57.1)	36.6 (1.7)	Outpatient clinic	Australia	6 weeks	1–2/week for 6 weeks (8–12 sessions)
Jüni et al. 2009	Back	Acute LBP	104 (35.6)	34.3 (9.4)	Hospital	Switzerland	2 weeks	2–3/week for 2 weeks (5 sessions)
Just and Stelzer 2009	Shoulder	SSPS	42 (42.9)	52.7 (?)	Outpatient clinic	Austria	4 weeks	1–2/week over 4 weeks (6 sessions)
Kachingwe et al. 2008	Shoulder	SSPS	33 (48.5)	46.4 (?)	Not specified	USA	6 weeks	1/week for 6 weeks (6 sessions)
Khan 2016	Neck	Cervical radiculopathy	100 (50.0)	38.0 (9.0)	Outpatient clinic	Pakistan	2 weeks	6/sessions per week for 2 weeks (12 sessions)
Kromer et al. 2013	Shoulder	SPSS	90 (51.1)	51.8 (11.2)	Outpatient clinic	Germany	5 weeks	5 weeks, 2/week (10 sessions)
Kulkarni and Kamat 2016	Knee	Knee OA	30 (?)	Unclear (?)	Hospital	India	3 days	1/day for 3 days (3 sessions)
Lalnunpuii et al. 2017	Knee	Knee OA	45 (100.0)	49.5 (5.5)	Not specified	India	4 weeks	3/week for 4 weeks (12 sessions)
Lau et al. 2011	Neck	Chronic neck pain	120 (50.0)	43.8 (9.3)	Outpatient clinic	China	4 weeks	2/week for 4 weeks (8 sessions)
Lee and Kim 2016	Neck	Chronic neck pain	46 (?)	42.3 (5.4)	Not specified	South Korea	10 weeks	3/week for 10 weeks (30 sessions)
Lytras et al. 2023	Neck	Chronic neck pain	80 (100.0)	49.5 (8.3)	Outpatient clinic	Greece	10 weeks	1/week for 10 weeks (10 sessions)
Maiers et al. 2014	Neck	Chronic neck pain	241 (45.0)	71.7 (5.2)	Not specified	USA	12 weeks	For 12 weeks 4 sessions and for IG 20 additional visits
Menek et al. 2019	Shoulder	SSPS	30 (40.0)	51.7 (6.6)	Not specified	Turkey	6 weeks	5/week for 6 weeks (30 sessions)
Michener et al. 2024	Shoulder	SSPS	93 (49.5)	53.1 (12.4)	Not specified	USA	6 weeks	1–2/week for 6 weeks (10 sessions)
Mintken et al. 2016	Shoulder	SSPS	140 (54.3)	40.5 (11.7)	Outpatient clinic	USA	4 weeks	2/week over 4 weeks (8 sessions)
Mostamand et al. 2023	Knee	Knee OA	31 (?)	59.6 (8.3)	Outpatient clinic	Iran	4 weeks	3–4/week for 4 weeks (12 sessions)
Murphy et al. 2010	Neck	Chronic neck pain	20 (73.3)	43.5 (9.0)	Rehabilitation facility	Canada	4 weeks manips 2/week for one group, 8 weeks 3/week exercise both groups (32 sessions for intervention, 24 sessions for control group)	
Nagata et al. 2019	TMJ	CMD	61 (82.0)	48.2 (21.1)	Hospital	Japan	Unclear	Unclear
Nam et al. 2013	Knee	Knee OA	30 (73.3)	66.1 (7.4)	Hospital	Korea	6 weeks	3/week for 6 weeks (18 sessions)
Narang and Ganvir 2014	Knee	Knee OA	50 (?)	Unclear	Not specified	India	2 weeks	Unclear
Naranjo‐Cinto et al. 2022	Shoulder	SSPS	45 (48.9)	35.7 (13.7)	Not specified	Spain	5 weeks	2/week for 5 weeks (10 sessions)
Nejati et al. 2019	Back	SI‐joint pain	56 (57.6)	46.8 (Range: 23–60)	Hospital	Iran	12 weeks	1/week for 12 weeks (12 sessions)
Nigam et al. 2021	Knee	Knee OA	40 (62.5)	58.5 (4.4)	Outpatient clinic	India	2 weeks	3/week for 2 weeks (6 sessions)
Ojoawo et al. 2016	Neck	Cervical radiculopathy	26 (46.2)	55.7 (5.4)	Hospital	Nigeria	4 weeks	3/week for 4 weeks (12 sessions)
Ojoawo and Olabode 2018	Neck	Cervical radiculopathy	75 (46.7)	55.7 (5.4)	Outpatient clinic	Nigeria	6 weeks	2/week for 6 weeks (12 sessions)
Park et al. 2020	Shoulder	SSPS	30 (70.0)	50.2 (9.0)	Not specified	Korea	3 weeks	3/week for 4 weeks (12 sessions)
Poulsen et al. 2013	Hip	Hip OA	118 (39.7)	65.8 (8.5)	Hospital	Denmark	6 weeks with 2/week MT sessions 15–25 min and two individual (30–45 min) and three group patient education sessions (1.5 h) for both groups (12 MT sessions and 5 PE sessions)	
Rasmussen et al. 2008	Back	Chronic LBP	72 (52.8)	38–42 (Range: 26–65)	Not specified	Denmark	Unclear	Unclear
Reynolds et al. 2020	TMJ	CMD	50 (86.0)	35.5 (13.4)	Not specified	USA	4 weeks	1/week for 4 weeks (4 sessions)
Rezaie et al. 2022	TMJ	CMD	30 (56.7)	27.7 (4.0)	Not specified	Iran	8 weeks	1–2/week for 8 weeks (10 sessions)
Rodríguez‐Sanz et al. 2020	Neck	Chronic neck pain	58 (70.7)	49.2 (14.5)	Hospital	Spain	4 weeks	1/week for 4 weeks (4 treatment sessions)
Rodríguez‐Sanz et al. 2022	Neck	Chronic neck pain	58 (70.7)	49.2 (15.9)	Not specified	Spain	20 min once	Only one treatment session
Sai and Kumar 2015	Shoulder	Frozen shoulder	68 (57.4)	51.0 (7.7)	Not specified	India	12 weeks	2/week for 12 weeks (24 sessions)
Satpute et al. 2015	Shoulder	SSPS	44 (43.2)	53.4 (7.1)	Outpatient clinic	India	3 weeks	3/week for 3 weeks (9 sessions)
Satpute et al. 2019	Back	Lumbar radiculopathy	60 (58.3)	45.9 (9.1)	Hospital	India	3 weeks	3/week for 2 weeks (6 sessions)
Schulz et al. 2019	Back	LBP	241 (51.5)	72.5 (5.6)	Not specified	USA	1–2/week for maximum of 12 weeks (16 for intervention, 4 for control group treatment sessions)	
Waqas et al. 2023	Back	Thoracic pain	100 (31.0)	36.0 (11.3)	Hospital	Pakistan	4 weeks	2/week for 4 weeks (8 sessions)
Razek and Shenouda 2014	Knee	Knee OA	45 (?)	51.9 (6.5)	Not specified	Egypt	4 weeks	3/week for 4 weeks (12 sessions)
Subhash and Makhija 2020	Shoulder	SSPS	32 (?)	32.4 (9.4)	Outpatient clinic	India	2 weeks	3/week for 2 weeks (6 sessions)
Tauqeer et al. 2024	Shoulder	SPSS	32 (?)	38.2 (7.3)	Not specified	Iran	4 weeks	3/week for 4 weeks (12 sessions)
Tuncer et al. 2013	TMJ	CMD	40 (77.5)	34.8 (Range: 18–72)	Not specified	Turkey	4 weeks	3/week for 4 weeks (12 sessions)
Ughreja and Shukla 2017	Knee	Knee OA	30 (73.3)	55.1 (8.9)	Not specified	India	1 week	7/week for 1 week (7 sessions)
UK Beam Team 2004	Back	LBP	1334 (56.1)	43.5 (11.7)	Hospital	UK	Treatment duration of 20 weeks: 1–2/week for 8 sessions of manipulation over 6 weeks, after 8 sessions of exercise and one refresher course at 12 weeks	
Yang et al. 2015	Neck	Chronic neck pain	30 (50.0)	30.8 (?)	Not specified	Korea	12 weeks	Unclear
Yiasemides et al. 2011	Shoulder	SPSS	98 (52.1)	62.0 (Range: 35–85)	Outpatient clinic	Australia	8 weeks	1–2/week for 8 weeks (12 sessions)

Abbreviations: CMD, craniomandibular dysfunction; LBP, low back pain; OA, osteoarthritis; SD, standard deviation; SPSS, subacromial shoulder pain syndrome; SSP, supraspinatus; TMJ, temporomandibular joint.

**FIGURE 2:**
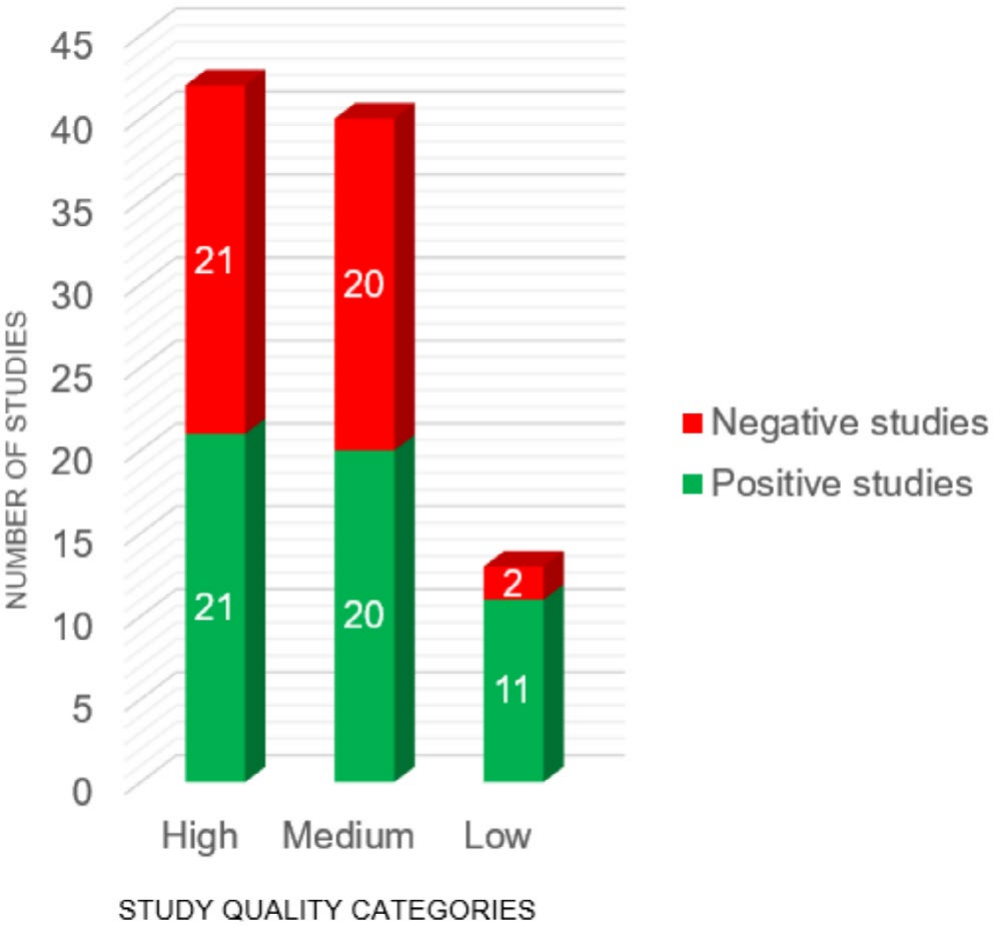
Proportion of positive and negative studies separated into low (0–6 points), medium (7–9 points), and high‐quality (10 or more points) studies according to the study quality assessment.

Although no consensus exists on thresholds for categorising study quality using the critical appraisal tool from the Joanna Briggs Institute (Barker et al. 2023), we classified studies as described in the methods section. Half of the medium‐quality (20/40, 50.0%) and high‐quality (21/42, 50.0%) studies reported statistically significant effects for the intervention group. In contrast, this proportion was substantially higher in low‐quality studies, where the vast majority (11/13, 84.6%) reported positive results for the intervention group. This trend is illustrated in Figure 2 below. Overall, these findings suggest a methodological bias in low‐quality studies. Such studies are more likely to produce statistically significant results, but these effects tend to diminish or disappear as study quality improves (Table 3).

**TABLE 3:** Inner‐group and in‐between group differences for pain and disability of included studies.

First author, year	Pain intensity at baseline and follow ups (1–10, NRS)		Disability mean at follow‐ups		Disability questionnaire	Statistical between group difference	Clinical relevance between group difference
	Intervention	Control	Intervention	Control	Questionnaire (scale, from‐to)		
Abbott et al. 2013	BL: 4.0. 1 year: 2.4. 2 years: 2.2	BL: 3.5. 1 year: 2.5. 2 years: 1.6	BL: 99.1. 1 year: 71.7. 2 years: 58.9	BL: 95.5. 1 year: 66.3. 2 years: 48.9	WOMAC (0–240) with a higher score indicating more disability	Not significant	n/a
Abbott et al. 2015	BL: 2.8. 1 year: 1.5	BL: 2.1. 1 year: 3.1	BL: 71.1. 1 year: 36.9	BL: 70.9. 1 year: 75.9	WOMAC (0–240) with a higher score indicating more disability	Significant at medium‐term FU	Clinically relevant for pain and disability
Akgüller et al. 2024	BL: 6.1. Immediate: 1.7	BL: 5.4. Immediate: 2.5	BL: 13.9. Immediate: 3.6	BL: 13.2. Immediate: 7.6	NDI (0–50) with a higher score indicating more disability	Significant at immediate FU	Clinically not relevant since MCID thresholds for pain intensity (< 1.5 points) and NDI (< 7.5) were not surpassed
Akhter et al. 2014	BL: 7.3. Immediate: 2.1. 3 months: 2.4	BL: 7.6. Immediate: 2.9. 3 months: 3.2	BL: 24.1. Immediate: 15.7. 3 months: 16.8	BL: 27.2. Immediate: 17.7. 3 months: 19.1	NDI (0–50) with a higher score indicating more disability	Not significant	n/a
Al‐Banawi et al. 2023	BL: 7.5. Immediate: 4.2	BL: 7.8. Immediate: 3.9	BL: 33.5. Immediate: 23.6	BL: 38.3. Immediate: 27.4	ODI (1–100) with a higher score indicating more disability	Not significant	n/a
Ali and Khan 2015	BL: 7.7. Immediate: 5.5	BL: 7.6. Immediate: 5.2	BL: 78.4. Immediate: 56.4	BL: 71.1. Immediate: 49.4	SPADI (0–100) with a higher score indicating more disability	Not significant	n/a
Azlin and Lyn 2011	BL: 4.1. Immediate: 2.3	BL: 3.3. Immediate: 2.6	n/a	n/a	n/a	Not significant	n/a
Bakken et al. 2021	BL: 4.7. Immediate: 3.6	BL: 4.2. Immediate: 3.1	BL: 22.6. Immediate: 20.5	BL: 21.7. Immediate: 19.8	NDI (0–50) with a higher score indicating more disability	Not significant	n/a
Bang and Deyle 2000	BL: 579.5. Immediate: 174.4	BL: 557.1. Immediate: 360.6	BL: 28.3. Immediate: 38.2	BL: 28.5. Immediate: 33.3	Study specific pain Composite score for pain assessment. Functional assessment questionnaire (0–45) with a higher score indicating less disability	Significant at immediate FU for pain intensity, unclear for disability	Clinically relevant, however, composite scores challenge the interpretability of the data
Barbosa et al. 2008	n/a	n/a	BL: 47.9. Immediate: 7.3	BL: 42.3. Immediate: 22.3	DASH (0–100) with a higher score indicating more disability	Significant at immediate FU for disability	Clinically relevant for disability since MCID threshold for disability was passed (4–15)
Bergman et al. 2004 NRS 0–28	BL: 17.8. Immediate: 12.1. 6 months: 11.9. 1 year: 11.1	BL: 17.9. Immediate: 14.2. 6 months: 12.7. 1 year: 12.4	BL: 58.6. Immediate: 32.0. 6 months: 25.6. 1 year: 22.3	BL: 60.7. Immediate: 42.4. 6 months: 40.4. 1 year: 33.0	SDQ (0–100) with a higher score indicating more disability	Significant for pain at immediate FU, not short‐/medium‐term. Significant for disability at short‐, not immediate and medium‐term	Clinically not relevant since MCID thresholds for pain intensity (< 1.5 points) and SDQ (< 14.0) were not surpassed
Blackman and Atkins 2014	BL: 6.2. Immediate: 4.4	BL: 3.9. Immediate: 3.4	BL: 38.0. Immediate: 42.0	BL: 43.0. Immediate: 49.4	LEFS (0–80) with a higher score indicating more function	Significant for pain, not disability at immediate FU	Clinically not relevant since MCID of for pain intensity (< 1.5 points) and LEFS (> 12.0) were not surpassed.
Bolton et al. 2020	BL: 19.6. Immediate: 26.1	BL: 20.2. Immediate: 27.6	BL: 94.2%. Immediate: 97.2%	BL: 93.5%. Immediate: 98.9%	AJFAT (0–40) and FAAM (0%–116%) with a higher score indicating more function	Not significant.	n/a
Bronfort et al. 2001	BL: 5.6. Immediate: 2.4. 3 months.: 3.0. 6 months.: 3.0. 1 year: 3.1	BL: 5.7. Immediate: 2.4. 3 months.: 2.5. 6 months: 3.0. 1 year: 3.0	BL: 26.1. Immediate: 14.1. 3 months.: 14.3. 6 months.: 14.8. 1 year: 16.1	BL: 26.7. Immediate: 12.4. 3 months.: 13.7. 6 months.: 15.0. 1 year: 15.6	NDI (0–50) with a higher score indicating more disability	Not significant	n/a
Bronfort et al. 2014	BL: 5.4. Immediate: 3.7. 1 year: 4.2	BL: 5.2. Immediate: 4.6. 1 year: 4.6	BL: 10.2. Immediate: 7.9. 1 year: 8.9	BL: 10.2. Immediate: 10.4. 1 year: 10.2	RMDQ (0–24) with a higher score indicating more disability	Significant for pain and disability at immediate FU, not at medium‐term FU	Clinically not relevant since MCID of pain intensity (< 1.5 points) and RMDQ (> 3.0) were not surpassed
Camargo et al. 2015	BL: 1.9. Immediate: 0.6	BL: 1.1. Immediate: 0.4	BL: 25.3. Immediate: 12.4	BL: 20.8. Immediate: 11.7	DASH (0–100) with a higher score indicating more disability	Not significant	n/a
Celenay et al. 2016	BL: 3.8. Immediate: 1.7	BL: 4.0. Immediate: 2.4	BL: 17.4. Immediate: 9.5	BL: 17.2. Immediate: 11.8	NDI (0–50) with a higher score indicating more disability	Significant for disability, not pain at immediate FU	Clinically not relevant since MCID for disability (> 7.5 points) on the NDI were not surpassed
Çelik 2016	BL: 5.3. Immediate: 0.4. 1 year: 0.2	BL: 5.3. Immediate: 0.9. 1 year: 0.4	BL: 50.7. Immediate: 14.4. 1 year: 5.1	BL: 54.3. Immediate: 22.3. 1 year: 11.5	DASH (0–100) with a higher score indicating more disability	Not significant	n/a
Ceylan et al. 2023	BL: 5.1. Immediate: 1.1	BL: 4.5. Immediate: 1.0	BL: 52.2. Immediate: 27.0	BL: 47.0. Immediate: 41.5	DASH (0–100) with a higher score indicating more disability	Significant for disability, not pain at immediate FU	Clinically relevant since MCID for disability (4–15 points) were reached
Chen et al. 2009	n/a	n/a	BL: 65.0. 6 months: 47.0	BL: 60.0. 6 months.: 43.0	SPADI (0–100) with a higher score indicating more disability	Not significant	n/a
Childs et al. 2004	BL: 5.7. ?	BL: 5.9. ?	BL: 41.4. ?	BL: 40.9. ?	ODI (1–100) with a higher score indicating more disability	Significant for pain and disability at immediate and short‐term FU with unclear values	Clinical relevance unclear since no numerical values are reported for any follow‐up
Conroy and Hayes 1998	BL: 5.0. Immediate: 1.3	BL: 4.8. Immediate: 4.6	n/a	n/a	n/a	Significant for pain at immediate FU	Clinically relevant since MCID for pain intensity (> 1.5 points) were surpassed
Cook et al. 2014	BL: 5.7. Immediate: 2.3	BL: 6.1. Immediate: 2.2	BL: 33.0. Immediate: 13.6	BL: 38.3. Immediate: 13.6	Quick DASH (1–100) with a higher score indicating more disability	Not significant.	n/a
Copurgensli et al. 2016	BL: 4.6. Immediate: 1.3. 1 month.: 0.7	BL: 4.7. Immediate: 1.2. 1 month.: 0.6	BL: 14.7. Immediate: 8.7. 1 month.: 5.1	BL: 13.2. Immediate: 7.8. 1 month.: 3.9	NDI (0–50) with a higher score indicating more disability	Not significant	n/a
Corum et al. 2018	BL: 4.1. Immediate: 1.6. 1 month.: 1.5	BL: 45. Immediate: 4.1. 1 month.: 3.5	BL: 56.0. Immediate: 63.1. 1 month.: 66.1	BL: 51.8. Immediate: 57.9. 1 month: 55.4	SF‐36 PCS (0–100) with a higher score indicating better functioning	Significant for pain and disability at immediate and short‐term FU	Clinically relevant since MCID for pain intensity (> 1.5 points) and SF‐36 PCS (> 1.8 Units) were surpassed
Delgado et al. 2020	BL: 5.2. 3 months: 4.0. 6 months: 3.6	BL: 5.2. 3 months: 2.4. 6 months: 2.2	BL: 36.1. 3 months: 17.1. 6 months: 14.4	BL: 34.2. 3 months: 28.8. 6 months: 28.3	Tinnitus handicap inventory (THI) (0–100) with a higher score indicating more disability	Significant for pain and disability at immediate, short‐term, and medium‐term FU	Clinically relevant since MCID for pain intensity (> 1.5 points) and THI (> 7.0 points) were surpassed
Dogan et al. 2021	BL: 4.0. Immediate: 3.0. 1 month: 1.4	BL: 3.5. Immediate: 2.2. 1 month: 1.7	BL: 70.7. Immediate: 73.7. 1 month: 78.8	BL: 69.5. Immediate: 78.5. 1 month: 81.1	SF‐36 PCS (0–100) with a higher score indicating better functioning	Not significant	n/a
Duymaz 2018	BL: 7.3. Immediate: 1.5	BL: 6.8. Immediate: 5.8	BL: 15.0. Immediate: 2.9	BL: 13.5. Immediate: 11.5	NDI (0–50) with a higher score indicating more disability	Significant for pain and disability at immediate FU	Clinically relevant since MCID for pain (> 1.5 points) and disability (> 7.5 points) on the NDI were surpassed
Dwyer et al. 2015	BL: 21.7. Immediate: 13.4	BL: 20.1. Immediate: 12.8	BL: 41.2. Immediate: 37.9	BL: 73.2. Immediate: 52.1	WOMAC pain (0–50) and function (0–170) subscales with higher scores indicating more impairment	Not significant	n/a
Dziedzic et al. 2005	BL: 5.4. Immediate: 3.8. 6 months: 3.6	BL: 4.6. Immed: 3.4. 6 months: 3.0	BL: 38.6. Immediate: 29.6. 6 months: 27.8	BL: 36.6. Immediate: 25.6. 6 months: 24.2	NPQ (0–36) with a higher score indicating more disability	Not significant	n/a
Eldesoky et al. 2019	BL: 7.9. Immediate: 2.3. 1 month: 2.4	BL: 7.3. Immediate: 4.0. 1 month: 4.3	BL: 49.4. Immediate: 8.3. 1 month: 8.7	BL: 47.2. Immediate: 17.1. 1 month: 17.7	NDI (0–50) with a higher score indicating more disability	Significant for pain and disability at immediate and short‐term FU	Clinically relevant since MCID for pain (> 1.5 points) and disability (> 7.5 points) on the NDI were surpassed
Eliason et al. 2021	n/a	n/a	BL: 40.7. 3 months: 64.9. 6 months: 68.5	BL: 38.3. 3 months: 59.1. 6 months: 66.6	Constant Murley Score (0–100) with a higher score indicating better functioning	Not significant	n/a
Espí‐López et al. 2020	BL: 4.8. Immed: 0.5. 1 month: 1.3	BL: 4.8. Immediate: 5.0. 1 month: 4.0	n/a	n/a	n/a	Significant for pain at immediate and short‐term FU	Clinically relevant since MCID for pain (> 1.5 points) were surpassed
Evans et al. 2012	BL: 5.6. Immediate: 2.3. 6 months: 3.3. 1 year: 3.4	BL: 5.7. Immediate: 2.7. 6 months: 3.1. 1 year: 3.6	BL: 27.8. Immediate: 14.5. 6 months: 17.3. 1 year: 18.0	BL: 26.1. Immediate: 16.0. 6 months: 16.8. 1 year: 17.5	NDI (0–50) with a higher score indicating more disability	Not significant	n/a
Farooq et al. 2018	BL: 6.0. Immediate: 2.0	BL: 5.6. Immediate: 3.2	BL: 35.6. Immediate: 12.1	BL: 31.1. Immediate: 19.4	NDI (0–50) with a higher score indicating more disability	Significant for pain and disability at immediate FU	Not clinically relevant since MCID for pain (> 1.5 points) and disability (> 7.5 points) on the NDI were not surpassed
Fathollahnejad et al. 2019	BL: 4.8. Immediate: 2.2. 1 month: 1.5	BL: 4.9. Immediate: 3.1. 1 month: 2.7	n/a	n/a	n/a	Significant for pain at immediate and short‐term FU	Not clinically relevant since MCID for pain (> 1.5 points) were not surpassed
Fitzgerald et al. 2016	BL: 5.7. 2 months: 3.3. 1 year: 3.9	BL: 5.4. 2 months: 3.2. 1 year: 4.1	BL: 88.1. 2 months: 42.4. 1 year: 57.4	BL: 87.1. 2 months: 46.9. 1 year: 55.4	WOMAC (0–240) with a higher score indicating more disability	Not significant	n/a
French et al. 2013	BL: 5.9. Immediate: 4.3. 4 months: 4.4	BL: 5.6. Immediate: 4.9. 4 months: 4.7	BL: 35.6. Immediate: 29.7. 4 months: 30.5	BL: 33.5. Immediate: 29.5. 4 months: 30.3	WOMAC physical function subscale (0–170) with a higher score indicating more disability	Not significant	n/a
Ganesh et al. 2015	BL: 6.7. Immediate: 2.4. 3 months: 2.2	BL: 5.9. Immediate: 1.7. 3 months: 1.2	BL: 33.9. Immediate: 17.2. 3 months: 13.2	BL: 34.8. Immediate: 10.2. 3 months: 6.7	NDI (0–50) with a higher score indicating more disability	Not significant	n/a
González‐Iglesias, Fernández‐de‐las‐Peñas, Cleland, Alburquerque‐Sendín, et al. 2009	BL: 5.6. Immediate: 2.3	BL: 5.4. Immediate: 4.3	BL: 27.8. Immediate: 15.2	BL: 27.1. Immediate: 22.9	NPQ (0–36) with a higher score indicating more disability	Significant for pain and disability at immediate FU	Clinically relevant since MCID for pain (> 1.5 points) and disability (> 3.0–5.0 points) were surpassed
González‐Iglesias, Fernández‐de‐las‐Peñas, Cleland, and Del Gutiérrez‐Vega 2009	BL: 5.5. Immediate: 2.0	BL: 5.3. Immediate: 4.5	BL: 27.9. Immediate: 15.2	BL: 27.0. Immediate: 23.1	NPQ (0–36) with a higher score indicating more disability.	Significant for pain and disability at immediate FU	Clinically relevant since MCID for pain (> 1.5 points) and disability (> 3.0–5.0 points) were surpassed
Gonzalez‐Rueda et al. 2021	BL: 6.8. Immediate: 3.6. 3 months: 4.0	BL: 5.9. Immediate: 4.5. 3 months: 4.5	BL: 13.2. Immediate: 5.4. 3 months: 4.4	BL: 11.6. Immediate: 9.7. 3 months: 7.6	NDI (0–50) with a higher score indicating more disability	Statistically for pain and disability at immediate FU and disability at short‐term	Not clinically relevant since MCID for pain (> 1.5 points) and disability (> 7.5 points) on the NDI were not surpassed
Grunnesjö et al. 2004	BL: 5.4. Immediate: 2.1. 2 months: 1.6	BL: 5.2. Immediate: 3.0. 2 months: 2.1	BL: 78.3. Immediate: 39.3. 2 months: 29.9	BL: 70.0. Immediate: 45.0. 2 months: 37.8	ODI (1–100) with a higher score indicating more disability	Statistically for disability, not pain, at immediate and short‐term FU	Not clinically relevant since MCID for disability (> 10.0) were not surpassed
Gutiérrez‐Espinoza et al. 2023	BL: 2.6. Immediate: 1.5	BL: 2.8. Immediate: 1.4	BL: 50.7. Immediate: 22.8	BL: 52.6. Immediate: 25.8	DASH (0–100) with a higher score indicating more disability	Not significant	n/a
Haider et al. 2018	BL: 5.1. Immediate: 0.7	BL: 5.4. Immediate: 2.3	BL: 40.3. Immediate: 12.3	BL: 43.1. Immediate: 22.6	SPADI (0–100) with a higher score indicating more disability	Statistically for pain and disability at immediate FU	Not clinically relevant since MCID for disability (< 14.0 points) were not, but clinically relevant MCID for pain (> 1.5 points) were surpassed
Hallegraeff et al. 2009	BL: 4.3. Immediate: 1.9	BL: 5.4. Immediate: 2.5	BL: 24.0. Immediate: 14.0	BL: 26.0. Immediate: 14.0	RMDQ (0–24) with a higher score indicating more disability	Not significant	n/a
Hancock et al. 2007	BL: 6.7. Unclear	BL: 6.3. Unclear	BL: 13.8. Unclear	BL: 12.5. Unclear	RMDQ (0–24) with a higher score indicating more disability	Not significant	n/a
Hoving et al. 2006	BL: 6.0. Immediate: 2.7. 1 year: 1.8	BL: 6.0. Immediate: 3.1. 1 year: 2.9	BL: 15.0. Immediate: 7.2. 1 year: 7.8	BL: 15.0. Immediate: 8.6. 1 year: 8.7	NDI (0–50) with a higher score indicating more disability	Not significant for disability, but significant for pain at immediate and mid‐term FU	Not clinically relevant since MCID for pain (> 1.5 points) were not surpassed
Javadov et al. 2021	BL: 4.8. Immediate: 2.3. 3 months: 1.3	BL: 4.8. Immediate: 3.4. 3 months: 3.1	BL: 57.4. Immediate: 57.4. 3 months: 85.0	BL: 59.6. Immediate: 75.2. 3 months: 75.9	SF‐36 PCS (0–100) with a higher score indicating better functioning	Statistically for pain at immediate and short‐term FU, not significant for disability	Not clinically relevant since MCID for pain (> 1.5 points) were not surpassed
Joshi et al. 2014	BL: 7.8. Immediate: 3.1	BL: 7.4. 3 months: 4.8	BL: 93.8. Immediate: 222.7	BL: 93.3. Immediate: 105.0	KOOS (0–500) with a higher score indicating more functioning	Statistically for pain and disability at immediate FU	Clinically relevant since MCID for pain (> 1.5 points) and disability (> 10.0 points) were surpassed
Jull et al. 2002	BL: 5.1. Immediate: 1.7. 1 year: 2.7	BL: 5.4. Immediate: 2.1. 1 yr.: 2.6	n/a	n/a	n/a	Not significant	n/a
Jüni et al. 2009	BL: 6.8. Unclear	BL: 6.3. Unclear	BL: 12.8. Unclear	BL: 14.3. Unclear	RMDQ (0–24) with a higher score indicating more disability	Not significant	n/a
Just and Stelzer 2009	BL: 4.6. Immediate: 2.9	BL: 5.8. Immediate: 4.0	BL: 47.5. Immediate: 62.4	BL: 46.6. Immediate: 61.5	Constant Murley Score (0–100) with a higher score indicating better functioning	Not significant	n/a
Kachingwe et al. 2008	BL: 6.3. Immediate: 3.5	BL: 5.7. Immediate: 4.5	BL: 53.1. Immediate: 23.0	BL: 62.4. Immediate: 23.7	SPADI (0–100) with a higher score indicating more disability	Not significant	n/a
Khan 2016	BL: 6.9. Immediate: 1.7	BL: 7.0. Immediate: 6.1	n/a	n/a	n/a	Significant for pain at immediate FU	Clinically relevant since MCID for pain (> 1.5 points) were surpassed
Kromer et al. 2013	BL: 5.2. Immediate: 2.9. 3 months: 2.3	BL: 5.0. Immediate: 3.3. 3 months: 2.3	BL: 39.7. Immediate: 23.5. 3 months: 16.1	BL: 4.1. Immediate: 26.8. 3 months: 19.8	SPADI (0–100) with a higher score indicating more disability	Not significant	n/a
Kulkarni and Kamat 2016	BL: 5.8. Immediate: 4.1	BL: 5.0. Immediate: 4.1	n/a	n/a	n/a	Not significant	n/a
Lalnunpuii et al. 2017	BL: 5.6. Immediate: 3.3	BL: 5.5. Immediate: 4.0	n/a	n/a	n/a	Not significant	n/a
Lau et al. 2011	BL: 5.0. Immediate: 3.1. 3 months: 3.3. 6 months: 3.0	BL: 5.1. Immediate: 4.4. 3 months: 4.4. 6 months: 4.2	BL: 39.2. Immediate: 27.2. 3 months: 27.8. 6 months: 28.8	BL: 41.9. Immediate: 36.0. 3 months: 35.4. 6 months: 34.8	NPQ (0–36) with a higher score indicating more disability	Significant for pain and disability at immediate and short‐term FU	Clinically relevant since MCID for disability (> 3.0–5.0 points) but not pain (< 1.5 points) was surpassed
Lee and Kim 2016	BL: 5.2. Immediate: 1.4	BL: 5.1. Immediate: 2.5	BL: 27.6. Immediate: 6.6	BL: 27.2. Immediate: 10.7	NDI (0–50) with a higher score indicating more disability	Significant for pain and disability at immediate FU	Not clinically relevant since MCID for pain (> 1.5 points) and disability (> 7.5 points) on the NDI were not surpassed
Lytras et al. 2023	BL: 5.5. Immediate: 2.0. 6 months: 2.3	BL: 5.5. Immediate: 3.0. 6 months: 3.4	BL: 32.5. Immediate: 16.5. 6 months: 18.9	BL: 31.8. Immediate: 20.7. 6 months: 25.7	NDI (0–50) with a higher score indicating more disability	Significant for pain and disability at immediate and short‐term FU	Not clinically relevant since MCID for pain (> 1.5 points) and disability (> 7.5 points) on the NDI were not surpassed
Maiers et al. 2014	BL: 5.3. Immediate: 2.3. 6 months: 2.9. 1 year: 3.1	BL: 4.9. Immediate: 3.2. 6 months: 3.3. 1 year: 3.2	BL: 22.8. Immediate: 14.4. 6 months: 14.8. 1 year: 15.8	BL: 24.2. Immediate: 16.9. 6 months: 17.7. 1 year: 18.3	NDI (0–50) with a higher score indicating more disability	Significant for pain at immediate and short‐term FU, not medium‐term FU. Not significant for disability	Not clinically relevant since MCID for pain (> 1.5 points) were not surpassed
Menek et al. 2019	BL: 5.9. Immediate: 0.7	BL: 4.9. Immediate: 1.8	BL: 50.9. Immediate: 18.9	BL: 53.2. Immediate: 33.2	DASH (0–100) with a higher score indicating more disability.	Significant for pain and disability at immediate FU	Clinically relevant for disability, not pain (< 1.5 points), since MCID threshold for disability was passed (4–15)
Michener et al. 2024	n/a	n/a	BL: 30.1. Immediate: 13.4. 6 months: 17.2. 1 year: 10.3	BL: 30.0. Immediate: 16.4. 6 months: 21.5. 1 year: 17.6	DASH (0–100) with a higher score indicating more disability.	Significant for disability not at immediate but at short‐ and medium‐term FU	Not clinically relevant for disability since MCID threshold for disability was not passed
Mintken et al. 2016	BL: 4.3. Immediate: 3.4. 1 month: 1.9. 6 months: 1.7	BL: 4.6. Immediate: 3.9. 1 month: 2.6. 6 months: 1.9	BL: 41.2. Immediate: 39.9. 1 month: 14.6. 6 months: 12.3	BL: 46.2. Immediate: 36.3. 1 month: 23.0. 6 months: 16.1	SPADI (0–100) with a higher score indicating more disability	Not significant	n/a
Mostamand et al. 2023	BL: 8.1. Immediate: 3.7	BL: 8.8. Immediate: 6.1	n/a	n/a	n/a	Significant for pain at immediate FU	Clinically relevant since MCID for pain (> 1.5 points) were surpassed
Murphy et al. 2010	BL: 3.4. Immediate: 1.1	BL: 3.3. Immediate: 1.4	BL: 25.0. Immediate: 14.3	BL: 24.9. Immediate: 16.6	NDI (0–50) with a higher score indicating more disability	Not significant	n/a
Nagata et al. 2019	BL: 5.8. Unclear	BL: 5.8. Unclear	n/a	n/a	n/a	Not significant	n/a
Nam et al. 2013	BL: 5.8. Immediate: 3.7	BL: 5.8. Immediate: 5.4	BL: 6.1. Immediate: 4.1	BL: 6.1. Immediate: 5.5	WOMAC physical function (0–68) with higher scores indicating more disability	Significant for pain and disability at immediate FU	Not clinically relevant for disability (< 10.0 points) clinically relevant for pain (> 1.5 points) since MCID was surpassed
Narang and Ganvir 2014	BL: 3.0. Immediate: 1.3	BL: 3.5. Immediate: 2.1	BL: 1.6. Immediate: 0.6	BL: 2.5. Immediate: 1.8	WOMAC physical function (0–68) with higher scores indicating more disability	Significant for pain and disability at immediate FU	Not clinically relevant for disability (< 10.0 points) clinically relevant for pain (> 1.5 points) since MCID was surpassed
Naranjo‐Cinto et al. 2022	BL: 3.4. Immediate: 3.3. 3 months: 0.7	BL: 3.5. Immediate: 2.7. 3 months: 1.2	BL: 31.1. Immediate: 24.9. 3 months: 9.6	BL: 25.7. Immediate: 20.0. 3 months: 13.1	SPADI (0–100) with a higher score indicating more disability	Not significant	n/a
Nejati et al. 2019	BL: 4.7. Immediate: 0.5. 3 months: 2.7	BL: 5.5. Immediate: 0.4. 3 months: 2.2	BL: 28.5. Immediate: 12.2. 3 months: 22.1	BL: 28.5. Immediate: 11.2. 3 months: 19.6	ODI (1–100) with a higher score indicating more disability	Not significant	n/a
Nigam et al. 2021	BL: 6.4. Immediate: 3.2. 3 months: 2.3. 6 months: 2.0	BL: 6.3. Immediate: 5.3. 3 months: 4.2. 6 months: 4.0	BL: 62.6. Immediate: 41.4. 3 months: 25.3. 6 months: 21.6	BL: 61.0. Immediate: 55.6. 3 months: 38.9. 6 months: 29.0	WOMAC (0–240) with a higher score indicating more disability	Significant for pain and disability at immediate and short‐term FU	Not clinically relevant for disability, but significant for pain (> 1.5 points), therefore clinically relevant
Ojoawo et al. 2016	BL: 7.6. Immediate: 2.7	BL: 7.8. Immediate: 3.7	BL: 58.7. Immediate: 16.3	BL: 55.3. Immediate: 21.5	NDI (0–50) with a higher score indicating more disability	Significant for pain, not disability at immediate FU	Not clinically relevant for pain (< 1.5 points), since MCID was not surpassed
Ojo Ojoawo, 2018^a^	BL: 7.6. Immediate: 2.7	BL: 7.8. Immediate: 3.8	BL: 58.7. Immediate: 16.3	BL: 55.3. Immediate: 21.5	NDI (0–50) with a higher score indicating more disability	Significant for pain, not disability at immediate FU	Not clinically relevant for pain (< 1.5 points), since MCID was not surpassed
Park et al. 2020	BL: 50.2. Immediate: 31.6	BL: 54.0. CG: 40.2	BL: 51.4. CG: 34.6	BL: 53.5. CG: 43.1	SPADI pain and disability subscales (0–100) with a higher score indicating more disability	Significant for pain and disability at immediate FU	Not clinically relevant for pain and disability since MCID (14–20 points) were not surpassed
Poulsen et al. 2013	BL: 5.4. Immediate: 3.5. 3 months: 4.0. 1 year: 4.0	BL: 5.1. Immediate: 5.3. 3 months: 5.5. 1 year: 4.9	BL: 68.0. Immediate: 84.0. 3 months: 80.0. 1 year: 79.0	BL: 68.0. Immediate: 69.0. 3 months: 69.0. 1 year: 69.0	HOOS (0–100) with a higher score indicating better functioning	Significant for pain and disability at immediate, short‐ and NOT medium‐term FU	Clinically relevant for pain and disability since MCID for pain (> 1.5 points) and disability (> 10.0 points) were surpassed
Rasmussen et al. 2008	BL: 5.0. Immediate: 3.0. 1 year: 2.0	BL: 5.0. Immediate: 3.0. 1 year: 2.0	n/a	n/a	n/a	Not significant	n/a
Reynolds et al. 2020	BL: 3.7. Immediate: 1.7	BL: 3.7. Immediate: 2.7	BL: 19.4. Immediate: 11.1	BL: 22.8. Immediate: 17.8	NDI (0–50) with a higher score indicating more disability	Not significant	n/a
Rezaie et al. 2022	BL: 5.6. Immediate: 1.7. 1 month: 2.4	BL: 5.4. Immediate: 4.2. 1 month: 4.1	n/a	n/a	n/a	Significant for pain at immediate FU	Clinically relevant since MCID for pain was surpassed (> 1.5 points)
Rodríguez‐Sanz et al. 2020	BL: 3.4. Immediate: 0.8. 3 months: 0.8. 6 months: 1.0	BL: 3.8. Immediate: 2.9. 3 months: 3.9. 6 months: 3.9	BL: 12.6. Immediate: 5.5. 3 months: 4.7. 6 months: 4.8	BL: 15.2. Immediate: 11.0. 3 months: 12.8. 6 months: 13.1	NDI (0–50) with a higher score indicating more disability	Significant for pain and disability at immediate and short‐term FU	Clinically relevant since MCID for pain (> 1.5 points) and disability (> 7.5 points) were surpassed
Rodríguez‐Sanz et al. 2022	BL: 3.4. Immediate: 1.6	BL: 3.8. Immediate: 3.8	n/a	n/a	n/a	Significant for pain at immediate FU	Clinically relevant since MCID for pain (> 1.5 points) was surpassed
Sai and Kumar 2015	BL: 6.6. Immediate: 2.5	BL: 6.3. Immediate: 4.5	n/a	n/a	n/a	Significant for pain at immediate FU	Clinically relevant since MCID for pain (> 1.5 points) was surpassed
Satpute et al. 2015	BL 8.1. Immediate: 2.8	BL: 8.0. Immediate: 4.6	BL: 64.5. Immediate: 23.9	BL: 65.2. Immediate: 46.1	SPADI (0–100) with a higher score indicating more disability	Significant for pain and disability at immediate FU	Clinically relevant since MCID for pain (> 1.5 points) and disability (14–20 points) were surpassed
Satpute et al. 2019	BL: 7.0. Immediate: 3.5. 3 months: 1.6. 6 months: 1.2	BL: 6.7. Immediate: 5.5. 3 months: 4.5. 6 months: 3.8	BL: 24.5. Immediate: 17.4. 3 months: 15.0. 6 months: 14.5	BL: 23.4. Immediate: 21.2. 3 months: 20.0. 6 months: 19.2	ODI (1–100) with a higher score indicating more disability	Significant for pain and disability at immediate‐ and short‐term FU	Clinically relevant since MCID for pain (> 1.5 points) and disability (> 5–10 points) were surpassed
Schulz et al. 2019	BL: 5.1. Immediate: 2.9. 3 months: 3.6. 1 year: 3.8	BL: 5.1. Immediate: 3.6. 3 months: 3.5. 1 year: 3.7	BL: 45.5. Immediate: 29.9. 3 months: 30.0. 1 year: 34.3	BL: 45.3. Immediate: 30.0. 3 months: 34.8. 1 year: 32.9	RMDQ (0–24) with a higher score indicating more disability	Not significant	n/a
Waqas et al. 2023	BL: 6.6. Immediate: 2.3. 3 months: 2.7	BL: 6.5. Immediate: 3.4. 3 months: 2.5	n/a	n/a	n/a	Significant for pain at immediate FU, not at short‐term FU	Not clinically relevant since MCID for pain (< 1.5 points) was not surpassed
Razek and Shenouda 2014	BL: 5.3. Immediate: 1.3	BL: 6.6. Immediate: 4.8	BL: 58.6. Immediate: 30.5	BL: 49.3. Immediate: 36.7	WOMAC (0–240) with a higher score indicating more disability	Significant for pain and disability at immediate FU	Clinically relevant for pain (> 1.5 points), not disability (< 14.0 points), considering the respective MCIDs
Subhash and Makhija 2020	BL: 5.3. Immediate: 2.7	BL: 5.7. Immediate: 3.7	BL: 41.3. Immediate: 26.3	BL: 40.3. Immediate: 27.5	SPADI (0–100) with a higher score indicating more disability	Significant for pain, not disability at immediate FU	Not clinically relevant for pain (< 1.5 points) since MCID was not surpassed
Tauqeer et al. 2024	BL: 5.6. Immediate: 2.2	BL: 6.0. Immediate: 4.6	BL: 26.1. Immediate: 21.3	BL: 19.7. Immediate: 16.3	DASH (0–100) with a higher score indicating more disability	Significant for pain, and control group significantly better in disability at immediate FU	Clinically relevant for pain (> 1.5 points), however, control group clinically relevant (4–15 points) better at DASH‐disability
Tuncer et al. 2013	BL: 2.3. Immediate: 0.1	BL: 1.8. Immediate: 0.5	n/a	n/a	n/a	Not significant	n/a
Ughreja and Shukla 2017	BL: 7.0. Immediate: 3.4	BL: 6.5. Immediate: 5.9	BL: 49.7. Immediate: 30.5	BL: 48.6. Immediate: 44.2	WOMAC (0–240) with a higher score indicating more disability	Significant for pain and disability at immediate FU	Clinically relevant for pain (> 1.5 points) and disability (> 14.0 points) since MCIDs were surpassed
UK Beam Team 2004	BL: 6.0. Immediate: 4.8. 1 year: 4.0	BL: 6.1. Immediate: 4.5. 1 year: 4.2	BL: 9.1. Immediate: 4.8. 1 year: 4.7	BL: 9.2. Immediate: 5.5. 1 year: 5.7	RMDQ (0–24) with a higher score indicating more disability	Not significant	n/a
Yang et al. 2015	BL: 7.1. Immediate: 1.9	BL: 6.9. Immediate: 3.4	n/a	n/a	n/a	Significant for pain at immediate FU	Clinically relevant for pain (> 1.5 points) since MCID was surpassed
Yiasemides et al. 2011	BL: 56.0. 1 month: 38.0. 3 months: 29.0. 6 months: 18.0	BL: 56.0. 1 month: 41. 3 months: 27.0. 6 months: 18.0	BL: 45.0. 1 month: 32.0. 3 months: 24.0. 6 months: 13.0	BL: 46.0. 1 month: 30.0. 3 months: 18.0. 6 months: 12.0	SPADI pain (0–100) and disability sub scores (0–100) with higher scores indicating more pain and disability	Not significant	n/a

*Note:* Immediate follow‐up (< 1 month); short‐term follow‐up (1–6 months); medium‐term follow‐up (7–12 months); long‐term follow‐up, more than 1 year.

Abbreviations: AJFAT, Ankle Joint Functional Assessment; BL, Baseline; DASH, Disability of Arm, Shoulder, and Hand; FAAM, Foot and Ankle Mobility Measure; FU, follow‐up; HOOS, Hip Disability and Osteoarthritis Outcome Score; KOOS, Knee Injury and Osteoarthritis Outcome Score; LEFS, Lower extremity Functional Scale; MCID, minimally clinically relevant difference; n/a, not applicable; NDI, Neck Disability Index; NPQ, Northwick Park Disability Questionnaire; NRS, numeric rating scale; ODI, Oswestry Disability Index; RMDQ, Roland Morris Disability Questionnaire; SD, standard deviation; SDQ, Strength and Difficulties Questionnaire; SF‐36‐PCS, Short‐Form‐36 Physical Component Score; SPADI, Shoulder and Pain Disability Index; WOMAC, Western Ontario and McMasters Universities Osteoarthritis Index.

^a^ Identical numbers as Ojoawo et al. 2016, methods are different but the probability that the numbers are identical for pain and disability is exceptionally low. Therefore, potential for double publication.

### 3.2 Evidence Summary

In most studies, MT was added to the intervention provided to the control group (66/95, 69.5%) without any formal control or additional time for the control group. Some studies (10/96, 10.4%) included MT in the treatment time, so the intervention group did not receive more attention or time, while in other studies the exact application of MT was unclear (15/95, 15.8%). Four studies controlled for the addition of MT using a sham intervention, such as placing hands on the painful region without performing any mobilisation. Of these, three showed no statistically significant differences (Hancock et al. 2007; Naranjo‐Cinto et al. 2022; Reynolds et al. 2020), indicating no effect of MT beyond the sham intervention. The one study reporting significant effects used a manipulation at another cervical spine segment as their sham intervention for the control group (Corum et al. 2018). This positive effect was apparent in the immediate follow‐up for pain intensity and disability.

### 3.3 Sustainability of Effects

From the 95 identified RCTs, only one study assessed the long‐term effectiveness of MT as an addition to exercise or usual care, and it showed no significant benefit (Abbott et al. 2013). Less than one in five studies (16/95, 15.8%) assessed medium‐term effects (7–12 months), of which only three (3/16, 3.2%) showed lasting benefits. Of these three studies, one surpassed the thresholds for the smallest worthwhile effect, demonstrating clinical relevance (Abbott et al. 2015).

**FIGURE 3:**
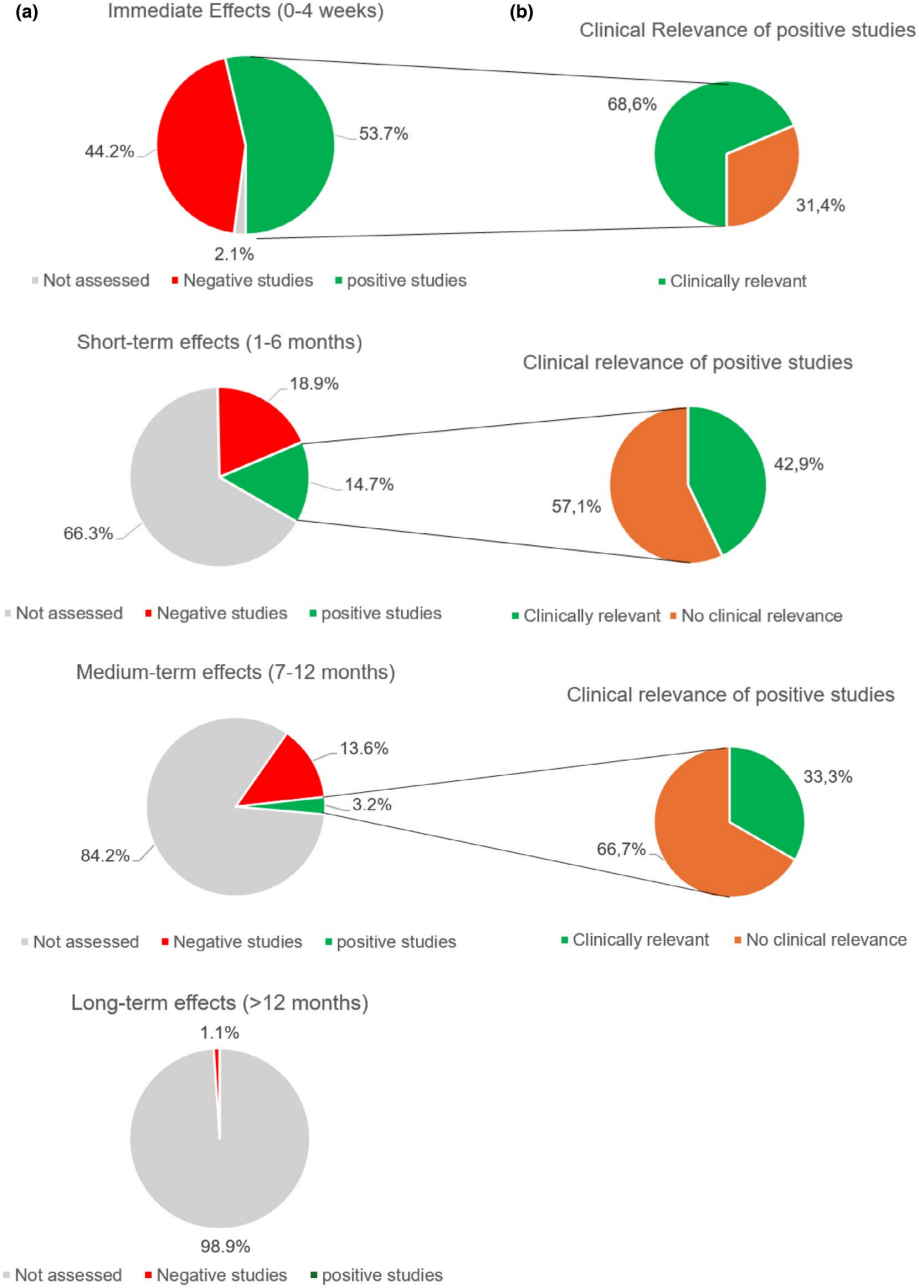
(a) Proportion of studies showing statistically significant effects of MT in addition to usual care, exercise, or physiotherapy, categorised into immediate‐, short‐, medium‐, and long‐term effects. (b) Proportion of statistically significant studies that surpassed thresholds for clinical relevance (i.e., the smallest worthwhile effect).

One of three studies (32/95, 33.6%) assessed short‐term effects (1–6 months). Of these, less than half (14/32, 43.8%) demonstrated benefits, of which again less than half (6/14, 42.9%) were clinically worthwhile. Lastly, almost all studies assessed immediate outcomes (93/95, 97.1%). Around half of these trials (51/93, 53.7%) showed a significant effect for the addition of MT, and two out of three (35/51, 68.6%) showed clinically relevant benefits. For illustrative purposes, these results are presented in Figure 3.

## 4 Discussion

The aim of this scoping review was to systematically compile studies that compare physiotherapy or exercise therapy to the same intervention with the addition of MT. We hypothesized that this study design would inherently favor primarily short‐term positive outcomes for pain and disability reduction with MT as an add‐on to usual care and little to no longer‐term effects. This would lead to unreliable results, questionable clinical relevance, and an inherent bias toward producing positive outcomes driven by non‐specific effects, with limited usefulness for both clinicians and researchers alike.

However, our findings suggest that short‐term effects are more inconclusive than expected. Of the 95 included studies, only half (53.7%) showed a significant benefit for MT at the immediate follow‐up. Long‐term effects were absent, with only one study following patients for more than a year (Abbott et al. 2013). Medium‐term effects were assessed in less than one in five studies (16%), of which the minority (3%) reported significant benefits (Abbott et al. 2015; Hoving et al. 2006; Michener et al. 2024), and only one of these reached clinical significance (Abbott et al. 2015). While MT has been shown to be as effective as other interventions for chronic LBP (Rubinstein et al. 2019), its role as an add‐on therapy remains unclear. The evidence largely points to inconclusive, short‐lived benefits of questionable clinical relevance for knee OA, for instance (Runge et al. 2022). French et al. concluded that all available adjunctive therapies, including MT, were not effective as add‐ons for hip‐ or knee OA in reducing pain or improving disability when added to exercise (French et al. 2022). Most studies identified by our review provided MT on top of usual care (66/95 RCTs) with only a minority using sham controls or some sort of control intervention (4/96 RCTs).

Broader evidence on MT's effectiveness (Grenier and Rothmund 2024; Lavazza et al. 2021; Molina‐Álvarez et al. 2022) shows it is often no better than sham interventions for reducing pain or improving function. The small, short‐lived effects observed in some studies are likely attributable to a combination of non‐specific contextual effects, patient and clinician expectations, biases, study design, physical contact, or neurophysiological influences (Bialosky et al. 2009, 2011; Keter et al. 2025). While contextual effects are integral and inevitable components of every musculoskeletal pain presentation (Poulter et al. 2024, 2025), the evidentiary bar for physiotherapy and manual therapy interventions must be higher than relying on this inherently flawed study design to justify their use. Considering the underwhelming evidence for MT as an add‐on intervention for atraumatic musculoskeletal pain, this review highlights several gaps in knowledge and research priorities.

### 4.1 Methodological Considerations

The identified studies raise concerns about the methodology used to evaluate MT. Comparing an intervention (e.g., MT) with another established intervention without adequately controlling for contextual effects, attention, and time with the clinician is an insufficient study design for establishing efficacy (Ernst and Lee 2008). Analogously, a medication trial would not be accepted if the test drug were added to an effective medication while the control group received no placebo (Ellenberg and Temple 2000; Juszczak et al. 2019; Mauri and D'Agostino 2017).

Although designing sham controls for non‐pharmacological interventions is more challenging than for medications, it is feasible (Bagg et al. 2022; Hohenschurz‐Schmidt, Draper‐Rodi, et al. 2023). Without such controls, these studies cannot justify MT as an intervention, given the inconclusive and heterogeneous evidence, even from designs biased toward producing positive results (Ernst and Lee 2008). In many cases, MT was added to usual care that included non‐guideline‐recommended interventions like a combination of electrotherapy and thermotherapy (González‐Iglesias, Fernández‐de‐las‐Peñas, Cleland, Alburquerque‐Sendín, et al. 2009; González‐Iglesias, Fernández‐de‐las‐Peñas, Cleland, and Del Gutiérrez‐Vega 2009), education as a stand‐alone treatment (Poulsen et al. 2013), or infrared therapy as standalone treatments. Such inappropriate control interventions likely overestimate the effects of MT, as noted in prior critiques (Hohenschurz‐Schmidt, Draper‐Rodi, et al. 2023, 2023). For LBP and knee OA, effect sizes for examined interventions are dependent on the control intervention, with passive and non‐guideline adherent interventions leading to the largest effect sizes for interventions (Bejarano et al. 2023; Marriott et al. 2024). In general, when a non‐pharmacological intervention like MT is controlled for in studies, authors often (61%) use a simulated manoeuvre, manual soft touch (18%), and detuned physical devices (12%) (Hohenschurz‐Schmidt, Draper‐Rodi, et al. 2023). While some authors argue that blinding is impossible in MT or physiotherapy and that controlling for contextual effects is impossible (Boutron et al. 2004), this notion has been refuted by recent evidence, demonstrating that successfully blinding non‐pharmacological interventions like MT with adequate sham interventions resembling the actual intervention is achievable, desirable, and is recommended when designing efficacy studies (Hohenschurz‐Schmidt, Draper‐Rodi, et al. 2023). Mechanistic considerations support the argument to design sham controls as structurally equal to the intervention as possible; manual touch has shown a larger placebo response than non‐manual controls (Hohenschurz‐Schmidt, Cherkin, et al. 2023).

Studies employing a ‘A vs. A + MT’ design are insufficient to justify MT in clinical practice. Without adequately controlling for attention, time, and contextual effects, these designs overestimate MT's effectiveness (Hohenschurz‐Schmidt, Draper‐Rodi, et al. 2023). This review also highlights a concerning trend in study reporting. Researchers often emphasize secondary benefits or minor improvements while downplaying negative or insignificant primary outcomes (Reynolds et al. 2020). Such reporting creates an unrealistically positive perception of MT's effectiveness, further compounded by publication bias, which inherently overestimates intervention efficacy (Dwan et al. 2008, 2013).

### 4.2 Recommendations

Clinicians should interpret these and similar studies cautiously. Researchers must improve study designs for non‐pharmacological pain interventions, including MT, to ensure robust and clinically meaningful conclusions. Helpful guidance and recommendations for advancing research quality have been published recently; for instance, researchers can rely on the TIDier‐Placebo checklist to accurately describe sham interventions when MT is added to usual care and controlled for with a sham intervention (Howick et al. 2020). Studies using inappropriate and non‐similar sham interventions tend to exaggerate the treatment effectiveness, mostly due to unblinding (Baskin et al. 2003). This is why researchers should ensure a similar composition of the sham intervention (Aycock et al. 2018; Benedetti et al. 2016).

### 4.3 Limitations

This scoping review is the first to provide a comprehensive summary of the evidence on MT's effectiveness as an add‐on intervention for musculoskeletal pain, offering valuable insights into this clinically relevant question. Still, it is not without its limitations. First, the heterogenous patient populations included in this review may impede clear conclusions considering the different pain presentations and mechanisms. However, the goal of this scoping review was to provide a comprehensive overview of the available evidence, so this limitation was also a strength since it broadened the scope of our work. Further, original studies often did not further specify the dosage, frequency, concept used, intensity, or rest times between interventions, neither for MT nor for exercise therapy or even duration of individual appointments.

## 5 Conclusion

The debate surrounding ‘A vs. A + B’ studies in non‐pharmacological interventions for atraumatic musculoskeletal pain remains unresolved. However, there is now compelling evidence showing that most studies, if any, demonstrate only immediate or short‐term effects for MT as an add‐on intervention, with questionable clinical relevance. If adequate sham or control interventions for any potential add‐on were used, the effects disappeared. Therefore, justifying MT based upon these studies is not based on solid evidence. Future studies should integrate an adequate sham into the control group and provide a rigorous description of the sham that was used.

## Author Contributions

J.‐P.G.: conceptualisation, methodology, investigation, writing: original draft preparation, writing: review and editing, data extraction, study protocol conceptualization and publication. A.T.: conceptualisation, writing original draft preparation, review and editing, data extraction, methodology.

## Disclosure

During the preparation of this work, the author(s) used CHATGPT 4.5 to ensure the correctness of language and improve the readability of our review. After using this tool/service, the authors reviewed and edited the content as needed and took full responsibility for the content of the publication.

## Ethics Statement

As this will be a scoping review, ethical approval is not required.

## Conflicts of Interest

The authors declare no conflicts of interest.

## Supporting information


**Data S1:** ejp70150‐sup‐0001‐DataS1.docx.

**Data S2:** ejp70150‐sup‐0002‐DataS2.docx.

**Data S3:** ejp70150‐sup‐0003‐DataS3.docx.

**Data S4:** ejp70150‐sup‐0004‐DataS4.docx.
