# Supplementary material for: Evaluating Manual Therapy in Musculoskeletal Pain: Why Certain Trial Designs May Overestimate Effectiveness—A Scoping Review
Source: Eur J Pain. 2025 Nov 13;29(10):e70150. doi: 10.1002/ejp.70150 (PMC12614156; doi:10.1002/ejp.70150)
Supplement: Supplementary file 1 — Data S1: ejp70150‐sup‐0001‐DataS1.docx. [file EJP-29-0-s004.docx]

**Literature Search**

| PubMed | ("manual therapy" OR "spinal manipulative therapy" OR "orthopaedic manual physical therapy" OR "orthopedic manual physical therapy" OR "orthopedic manual therapy" OR "non-thrust-mobilization") AND (pain or osteoarthritis or "Low back pain" OR tendinopathy) AND ("usual care" or "routine care" or "standard care" or physiotherapy or "physical therapy" or exercise or sham or "sham manual therapy") AND ("in addition" or combin* or added) |
| --- | --- |
| EBSCo | ("manual therapy" OR "spinal manipulative therapy" OR "orthopaedic manual physical therapy" OR "orthopedic manual physical therapy" OR "orthopedic manual therapy" OR "non-thrust-mobilization") AND (pain or osteoarthritis or "Low back pain" OR tendinopathy) AND ("usual care" or "routine care" or "standard care" or physiotherapy or "physical therapy" or exercise or sham or "sham manual therapy") AND ("in addition" or combin* or added) |
| PEDro | Abtract&Title: manual therapy  Problem: pain  Subdiscipline: musculoskeletal  When Searching: Match all Search Terms (AND) |
