# Supplementary material for: Evaluating Manual Therapy in Musculoskeletal Pain: Why Certain Trial Designs May Overestimate Effectiveness—A Scoping Review
Source: Eur J Pain. 2025 Nov 13;29(10):e70150. doi: 10.1002/ejp.70150 (PMC12614156; doi:10.1002/ejp.70150)
Supplement: Supplementary file 2 — Data S2: ejp70150‐sup‐0002‐DataS2.docx. [file EJP-29-0-s001.docx]

| Full-text studies excluded from this systematic review with reasons for exclusion | | | |
| --- | --- | --- | --- |
| Author | Year | Study title | Reason for exclusion |
| Rodriguez-Sanz | 2021 | Comparison of an exercise program with and without manual therapy for patients with chronic neck pain and upper cervical rotation restriction. Randomized controlled trial | Double publication, exactly same data set as Rodriguez-Sanz from 2020 used, same methods, same female-male ratio, same clinical trials registry number |
| Reza | 2021 | Efficacy of Specified Manual Therapies in Combination with a Supervised Exercise Protocol for Managing Pain Intensity and Functional Disability in Patients with Knee Osteoarthritis | The intervention examined was soft tissue manipulations not joint manual therapy was applied. |
| Cruser | 2012 | A randomized, controlled trial of osteopathic manipulative treatment for acute low back pain in active-duty military personnel | Excluded because intervention was specifically titled osteopathic manipulation |
| Vismara | 2012 | Osteopathic manipulative treatment in obese patients with chronic low back pain: a pilot study | Excluded because intervention was specifically titled osteopathic manipulation |
| Macdonald | 1990 | An open controlled assessment of osteopathic manipulation in nonspecific low-back pain | Excluded because intervention was specifically titled osteopathic manipulation |
| Tat | 2021 | The effects of manual therapy and exercises on pain, muscle strength, joint health, functionality, and quality of life in haemophilic arthropathy of the elbow joint: A randomized controlled pilot study | The patient population examined was not compatible with inclusion and exclusion criteria, since a specific medical condition, surgery or traumatic event was causative to the pain. |
| Argut | 2021 | The Combination of Exercise and Manual Therapy Versus Exercise Alone in Total Knee Arthroplasty Rehabilitation: A Randomized Controlled Clinical Trial | The patient population was not eligible since it was patients after total knee arthroplasty. |
| Cleland | 2013 | Manual physical therapy and exercise versus supervised home exercise in the management of patients with inversion ankle sprain: a multicentre randomized clinical trial | The patient population was not eligible since it was patients after inverse ankle sprain. |
| Plaza-Manzano | 2016 | Manual therapy in joint and nerve structures combined with exercises in the treatment of recurrent ankle sprains: A randomized, controlled trial | The patient population was not eligible since it was patients after recurrent ankle sprains. |
| Tomruk | 2020 | Effects of early manual therapy on functional outcomes after volar plating of distal radius fractures: A randomized controlled trial | The patient population was not eligible since it was patients after distal radius fracture. |
| Lal | 2017 | Efficacy of Passive Mobilization and Home Exercises in Post Immobilization Period of Distal Radius Fracture | The patient population was not eligible since it was patients after distal radius fracture. |
| Guiterrez-Espinoza | 2022 | Effectiveness of manual therapy in patients with distal radius fracture: a systematic review and meta-analysis | The patient population was not eligible since it was a systematic review of patients after distal radius fracture. |
| Paco do Amaral | 2012 | Manual therapy associated with upper limb exercises vs. exercises alone for shoulder rehabilitation in postoperative breast cancer | The patient population was not eligible since it was patients after breast cancer. |
| Evans | 2019 | Spinal manipulation and exercise for low back pain in adolescents: a randomized trial | The patient population was not eligible since it was adolescents, and this review focusses on adults. |
| Abbott | 2019 | Incremental clinical effectiveness and cost effectiveness of providing supervised physiotherapy in addition to usual medical care in patients with osteoarthritis of the hip or knee: 2-year results of the MOA randomised controlled trial | Excluded since this study is a secondary analysis of a primary study which has already been included in this systematic review. |
| Bove | 2018 | Exercise, Manual Therapy, and Booster Sessions in Knee Osteoarthritis: Cost-Effectiveness Analysis From a Multicenter Randomized Controlled Trial | Excluded since this study is a secondary analysis of a primary study which has already been included in this systematic review. |
| Chaibi | 2017 | Adverse events in a chiropractic spinal manipulative therapy single-blinded, placebo, randomized controlled trial for migraineurs | Excluded since this study is a secondary analysis of a primary study which has already been included in this systematic review. |
| Kromer | 2014 | Effectiveness of physiotherapy and costs in patients with clinical signs of shoulder impingement syndrome: One-year follow-up of a randomized controlled trial | Excluded since this study is a secondary analysis of a primary study which has already been included in this systematic review. |
| Leininger | 2012 | Exploring patient satisfaction: a secondary analysis of a randomized clinical trial of spinal manipulation, home exercise, and medication for acute and subacute neck pain | Excluded since this study is a secondary analysis of a primary study which has already been included in this systematic review. |
| Lewis | 2007 | An economic evaluation of three physiotherapy treatments for non-specific neck disorders alongside a randomized trial | Excluded since this study is a secondary analysis of a primary study which has already been included in this systematic review. |
| Pryymachenko | 2021 | Are manual therapy or booster sessions worthwhile in addition to exercise therapy for knee osteoarthritis: Economic evaluation and 2-year follow-up of a randomized controlled trial | Excluded since this study is a secondary analysis of a primary study which has already been included in this systematic review. |
| Singh | 2012 | An Experimental Study on effects of Mulligan Mobilization Technique and Isometric Exercises in Patients with Osteoarthritis Knee | Excluded because relevant outcomes for inclusion in this review were either missing or not assessed at all. |
| Romero | 2015 | Mobilization With Movement for Shoulder Dysfunction in Older Adults: A Pilot Trial | Excluded because relevant outcomes for inclusion in this review were either missing or not assessed at all. |
| Chaibi | 2017 | Chiropractic spinal manipulative therapy for migraine: a three-armed, single-blinded, placebo, randomized controlled trial | Excluded because relevant outcomes for inclusion in this review were either missing or not assessed at all. |
| Caracole | 2022 | The effectiveness of pain neuroscience education combined with manual therapy and home exercise for chronic low back pain: A single-blind randomized controlled trial. | Excluded because relevant outcomes for inclusion in this review were either missing or not assessed at all. |
| Senbursa | 2011 | The effectiveness of manual therapy in supraspinatus tendinopathy | Excluded because relevant outcomes for inclusion in this review were either missing or not assessed at all. |
| Ko | 2009 | Manual Therapy and Exercise for OA Knee: Effects on Muscle Strength, Proprioception, and Functional Performance | Excluded because relevant outcomes for inclusion in this review were either missing or not assessed at all. |
| Bialoszewski | 2011 | Usefulness of manual therapy in the rehabilitation of patients with chronic rotator cuff injuries. Preliminary report | Excluded because the full-text version was either not in English, not accessible through multiple university channels and upon personal request or retracted. |
| Gutierrez-Espinoza | 2015 | Glenohumeral posterior mobilization versus conventional physiotherapy for primary adhesive capsulitis: a randomized clinical trial | Excluded because the full-text version was either not in English, not accessible through multiple university channels and upon personal request or retracted. |
| Taylor | 1994 | ? | Excluded because the full-text version was either not in English, not accessible through multiple university channels and upon personal request or retracted. |
| Nelson | 1998 | The efficacy of spinal manipulation, amitriptyline, and the combination of both therapies for the prophylaxis of migraine headache | Excluded because the full-text version was either not in English, not accessible through multiple university channels and upon personal request or retracted. |
| Herrera-Valencia | 2020 | Efficacy of Manual Therapy in TemporomandibularJoint Disorders and Its Medium-and Long-Term Effects on Pain and Maximum Mouth Opening: A Systematic Review and Meta-Analysis | This systematic review was checked for relevant primary studies (RCTs) for this review but either no additional or no eligible studies at all were identified or extracted from this systematic review. |
| Liu | 2024 | Efficacy of manual therapy on shoulder pain and function in patients with rotator cuff injury: A systematic review and meta‑analysis | This systematic review was checked for relevant primary studies (RCTs) for this review but either no additional or no eligible studies at all were identified or extracted from this systematic review. |
| Silveira | 2024 | Shoulder specific exercise therapy is effective in reducing chronic shoulder pain: A network meta-analysis. | This systematic review was checked for relevant primary studies (RCTs) for this review but either no additional or no eligible studies at all were identified or extracted from this systematic review. |
| Qin | 2024 | Tension-Type Headache Management: A Systematic Review and Network Meta-analysis of Complementary and Alternative Medicine | This systematic review was checked for relevant primary studies (RCTs) for this review but either no additional or no eligible studies at all were identified or extracted from this systematic review. |
| Zhu | 2024 | The effects of manual therapy in pain and safety of patients with knee osteoarthritis: a systematic review and meta-analysis | This systematic review was checked for relevant primary studies (RCTs) for this review but either no additional or no eligible studies at all were identified or extracted from this systematic review. |
| Yoma | 2022 | The Effect of Exercise Therapy Interventions on Shoulder Pain and Musculoskeletal Risk Factors for Shoulder Pain in Competitive Swimmers: A Scoping Review | This systematic review was checked for relevant primary studies (RCTs) for this review but either no additional or no eligible studies at all were identified or extracted from this systematic review. |
| Yao | 2021 | Clinical Evidence for the Effects of Manual Therapy on Cancer Pain: A Systematic Review and Meta-Analysis | This systematic review was checked for relevant primary studies (RCTs) for this review but either no additional or no eligible studies at all were identified or extracted from this systematic review. |
| Van der Welde | 2016 | Which interventions are cost-effective for the management of whiplash-associated and neck pain-associated disorders? A systematic review of the health economic literature by the Ontario Protocol for Traffic Injury Management (OPTIMa) Collaboration | This systematic review was checked for relevant primary studies (RCTs) for this review but either no additional or no eligible studies at all were identified or extracted from this systematic review. |
| Sampath | 2016 | The effects of manual therapy or exercise therapy or both in people with hip osteoarthritis: a systematic review and meta-analysis | This systematic review was checked for relevant primary studies (RCTs) for this review but either no additional or no eligible studies at all were identified or extracted from this systematic review. |
| Pattanshetty | 2022 | Role of Manual Therapy for Neck Pain and Quality of Life in Head and Neck Cancer Survivors: A Systematic Review | This systematic review was checked for relevant primary studies (RCTs) for this review but either no additional or no eligible studies at all were identified or extracted from this systematic review. |
| Neal | 2022 | Six Treatments Have Positive Effects at 3 Months for People with Patellofemoral Pain: A Systematic Review with Meta-analysis | This systematic review was checked for relevant primary studies (RCTs) for this review but either no additional or no eligible studies at all were identified or extracted from this systematic review. |
| Minkalis | 2018 | A systematic review of thrust manipulation combined with one conservative intervention for rotator cuff and related non-surgical shoulder conditions | This systematic review was checked for relevant primary studies (RCTs) for this review but either no additional or no eligible studies at all were identified or extracted from this systematic review. |
| Landesa-Pineiro | 2022 | Physiotherapy treatment of lateral epicondylitis: A systematic review | This systematic review was checked for relevant primary studies (RCTs) for this review but either no additional or no eligible studies at all were identified or extracted from this systematic review. |
| Lahuerta-Martin | 2023 | The effectiveness of non-surgical interventions in athletes with groin pain: a systematic review and meta-analysis | This systematic review was checked for relevant primary studies (RCTs) for this review but either no additional or no eligible studies at all were identified or extracted from this systematic review. |
| Jung | 2022 | Effectiveness of physiotherapy interventions on headache intensity, frequency, duration and quality of life of patients with tension-type headache. A systematic review and network meta-analysis | This systematic review was checked for relevant primary studies (RCTs) for this review but either no additional or no eligible studies at all were identified or extracted from this systematic review. |
| Hohenschurz-Schmidt | 2023 | Blinding and sham control methods in trials of physical, psychological, and self-management interventions for pain (article II): a meta-analysis relating methods to trial results | This systematic review was checked for relevant primary studies (RCTs) for this review but either no additional or no eligible studies at all were identified or extracted from this systematic review. |
| Hernando-Garijo | 2023 | Effectiveness of non-pharmacological conservative therapies in adults with fibromyalgia: A systematic review of high-quality clinical trials | This systematic review was checked for relevant primary studies (RCTs) for this review but either no additional or no eligible studies at all were identified or extracted from this systematic review. |
| Gonzalez-Sanchez | 2023 | Temporomandibular Joint Dysfunctions: A Systematic Review of Treatment Approaches | This systematic review was checked for relevant primary studies (RCTs) for this review but either no additional or no eligible studies at all were identified or extracted from this systematic review. |
| Gliedt | 2022 | Manual therapy interventions in the management of adults with prior cervical spine surgery for degenerative conditions: a scoping review | This systematic review was checked for relevant primary studies (RCTs) for this review but either no additional or no eligible studies at all were identified or extracted from this systematic review. |
| Gebremariam | 2013 | Subacromial impingement syndrome--effectiveness of physiotherapy and manual therapy | This systematic review was checked for relevant primary studies (RCTs) for this review but either no additional or no eligible studies at all were identified or extracted from this systematic review. |
| Espi-Lopez | 2017 | Effectiveness of Manual Therapy Combined with Physical Therapy in Treatment of Patellofemoral Pain Syndrome: Systematic Review | This systematic review was checked for relevant primary studies (RCTs) for this review but either no additional or no eligible studies at all were identified or extracted from this systematic review. |
| Eckenrode | 2018 | Effectiveness of Manual Therapy for Pain and Self-reported Function in Individuals with Patellofemoral Pain: Systematic Review and Meta-analysis | This systematic review was checked for relevant primary studies (RCTs) for this review but either no additional or no eligible studies at all were identified or extracted from this systematic review. |
| Cumplido-Trasmonte | 2021 | Manual therapy in adults with tension-type headache: A systematic review | This systematic review was checked for relevant primary studies (RCTs) for this review but either no additional or no eligible studies at all were identified or extracted from this systematic review. |
| Chaibi | 2021 | Spinal Manipulative Therapy for Acute Neck Pain: A Systematic Review and Meta-Analysis of Randomised Controlled Trials | This systematic review was checked for relevant primary studies (RCTs) for this review but either no additional or no eligible studies at all were identified or extracted from this systematic review. |
| Ceballos-Laita | 2019 | Effects of non-pharmacological conservative treatment on pain, range of motion and physical function in patients with mild to moderate hip osteoarthritis. A systematic review | This systematic review was checked for relevant primary studies (RCTs) for this review but either no additional or no eligible studies at all were identified or extracted from this systematic review. |
| Castellini | 2022 | Some conservative interventions are more effective than others for people with chronic non-specific neck pain: a systematic review and network meta-analysis. | This systematic review was checked for relevant primary studies (RCTs) for this review but either no additional or no eligible studies at all were identified or extracted from this systematic review. |
| Beumer | 2016 | Effects of exercise and manual therapy on pain associated with hip osteoarthritis: a systematic review and meta-analysis. | This systematic review was checked for relevant primary studies (RCTs) for this review but either no additional or no eligible studies at all were identified or extracted from this systematic review. |
| Bednarczyk | 2024 | The effectiveness of cervical rehabilitation interventions for pain in adults with myogenic temporomandibular disorders: A systematic review and meta-analysis | This systematic review was checked for relevant primary studies (RCTs) for this review but either no additional or no eligible studies at all were identified or extracted from this systematic review. |
| Beltran-Alacreu | 2015 | Manual Therapy, Therapeutic Patient Education, and Therapeutic Exercise, an Effective Multimodal Treatment of Nonspecific Chronic Neck Pain: A Randomized Controlled Trial | Study design not eligible for this systematic review, since not manual therapy as an isolated intervention was added to exercise or usual care. |
| Bronfort | 2012 | Spinal manipulation, medication, or home exercise with advice for acute and subacute neck pain: a randomized trial | Study design not eligible for this systematic review, since not manual therapy as an isolated intervention was added to exercise or usual care. |
| Castien | 2011 | Effectiveness of manual therapy for chronic tension-type headache: a pragmatic, randomised, clinical trial | Study design not eligible for this systematic review, since not manual therapy as an isolated intervention was added to exercise or usual care. |
| Yeole | 2017 | Effectiveness of Movement with Mobilization in Adhesive Capsulitis of Shoulder Randomized Controlled Trial. | Study design not eligible for this systematic review, since not manual therapy as an isolated intervention was added to exercise or usual care. |
| Cuesta-Barriuso | 2021 | The effectiveness of manual therapy in addition to passive stretching exercises in the treatment of patients with haemophilic knee arthropathy: A randomized, single-blind clinical trial | Study design not eligible for this systematic review, since not manual therapy as an isolated intervention was added to exercise or usual care. |
| Deyle | 2005 | Physical Therapy Treatment Effectiveness for Osteoarthritis of the Knee: A Randomized Comparison of Supervised Clinical Exercise and Manual Therapy Procedures Versus a Home Exercise Program | Study design not eligible for this systematic review, since not manual therapy as an isolated intervention was added to exercise or usual care. |
| Domingues | 2019 | Is a combined programme of manual therapy and exercise more effective than usual care in patients with non-specific chronic neck pain? A randomized controlled trial | Study design not eligible for this systematic review, since not manual therapy as an isolated intervention was added to exercise or usual care. |
| Espi-Lopez | 2014 | Efficacy of Manual and Manipulative Therapy in the Perception of Pain and Cervical Motion in Patients with Tension-Type Headache: A Randomized, Controlled Clinical Trial | Study design not eligible for this systematic review, since not manual therapy as an isolated intervention was added to exercise or usual care. |
| Espi-Lopez | 2014 | Effect of manual therapy techniques on headache disability in patients with tension-type headache. Randomized controlled trial | Study design not eligible for this systematic review, since not manual therapy as an isolated intervention was added to exercise or usual care. |
| Espi-Lopez | 2016 | Do manual therapy techniques have a positive effect on quality of life in people with tension-type headache? A randomized controlled trial | Study design not eligible for this systematic review, since not manual therapy as an isolated intervention was added to exercise or usual care. |
| Ford | 2018 | Individualised manual therapy plus guideline-based advice vs advice alone for people with clinical features of lumbar zygapophyseal joint pain: a randomised controlled trial | Study design not eligible for this systematic review, since not manual therapy as an isolated intervention was added to exercise or usual care. |
| Ghodrati | 2019 | Adding Temporomandibular joint treatments to routine physiotherapy for patients with non-specific chronic neck pain: A randomized clinical study | Study design not eligible for this systematic review, since not manual therapy as an isolated intervention was added to exercise or usual care. |
| Gonzalez-Rueda | 2020 | Does Upper Cervical Manual Therapy Provide Additional Benefit in Disability and Mobility over a Physiotherapy Primary Care Program for Chronic Cervicalgia? A Randomized Controlled Trial | Study design not eligible for this systematic review, since not manual therapy as an isolated intervention was added to exercise or usual care. |
| Heggannavar | 2015 | QUANTITATIVE EFFECTS OF PROPRIOCEPTIVE EXERCISES AND MULLIGAN’S MWM IN SUBJECTS WITH OSTEOARTHRITIS OF KNEE – A RANDOMIZED CLINICAL TRAIL | Excluded because the full-text version was not available since this was only published as an abstract. |
| Maiers | 2019 | Short- or Long-Term Treatment of Spinal Disability in Older Adults with Manipulation and Exercise | Study design not eligible for this systematic review, since not manual therapy as an isolated intervention was added to exercise or usual care. |
| Neelapala | 2016 | EFFECT OF MULLIGAN’S POSTEROLATERAL GLIDE ON SHOULDER ROTATOR STRENGTH, SCAPULAR UPWARD ROTATION IN SHOULDER PAIN SUBJECTS – A RANDOMIZED CONTROLLED TRIAL | Study design not eligible for this systematic review, since not manual therapy as an isolated intervention was added to exercise or usual care. |
| Peterson | 2013 | The effect of manual therapy with augmentative exercises for neck pain: a randomised clinical trial | Study design not eligible for this systematic review, since not manual therapy as an isolated intervention was added to exercise or usual care. |
| Ribeiro | 2024 | Mediators of the effects of exercise and manual therapy for people with knee and hip osteoarthritis: A secondary, exploratory analysis of the MOA trial | Excluded since this study is a secondary analysis of a primary study which has already been included in this systematic review. |
| Riley | 2015 | Short-term effects of thoracic spinal manipulations and message conveyed by clinicians to patients with musculoskeletal shoulder symptoms: a randomized clinical trial | Study design not eligible for this systematic review, since not manual therapy as an isolated intervention was added to exercise or usual care. |
| Sharma | 2021 | Effects of exercise therapy plus manual therapy on muscle activity, latency timing and SPADI score in shoulder impingement syndrome | Study design not eligible for this systematic review, since not manual therapy as an isolated intervention was added to exercise or usual care. |
| Sharma | 2021 | Progressive Resistance Exercises Plus Manual Therapy Is Effective in Improving Isometric Strength in Overhead Athletes with Shoulder Impingement Syndrome: A Randomized Controlled Trial | Study design not eligible for this systematic review, since not manual therapy as an isolated intervention was added to exercise or usual care. |
| Sremakaew | 2023 | Effectiveness of adding rehabilitation of cervical related sensorimotor control to manual therapy and exercise for neck pain: A randomized controlled trial | Study design not eligible for this systematic review, since not manual therapy as an isolated intervention was added to exercise or usual care. |
| Walker | 2008 | The Effectiveness of Manual Physical Therapy and Exercise for Mechanical Neck Pain  A Randomized Clinical Trial | Study design not eligible for this systematic review, since not manual therapy as an isolated intervention was added to exercise or usual care. |
| Zaworski | 2021 | The effectiveness of manual therapy and proprioceptive neuromuscular facilitation compared to kinesiotherapy: a four-arm randomized controlled trial | Study design not eligible for this systematic review, since not manual therapy as an isolated intervention was added to exercise or usual care. |
| Rompe | 2001 | Chronic lateral epicondylitis of the elbow: A prospective study of low-energy shockwave therapy and low-energy shockwave therapy plus manual therapy of the cervical spine | Study design not eligible for this systematic review, since not manual therapy as an isolated intervention was added to exercise or usual care. |
| Vinuesa-Montoya | 2017 | A Preliminary Randomized Clinical Trial on the Effect of Cervicothoracic Manipulation Plus Supervised Exercises vs a Home Exercise Program for the Treatment of Shoulder Impingement | Study design not eligible for this systematic review, since not manual therapy as an isolated intervention was added to exercise or usual care. |
| Kasimis | 2024 | Effects of Manual Therapy Plus Pain Neuroscience Education with Integrated Motivational Interviewing in Individuals with Chronic Non-Specific Low Back Pain: A Randomized Clinical Trial Study | Study design not eligible for this systematic review, since not manual therapy as an isolated intervention was added to exercise or usual care. |
| Senbursa | 2007 | Comparison of conservative treatment with and without manual physical therapy for patients with shoulder impingement syndrome: a prospective, randomized clinical trial | Study design not eligible for this systematic review, since not manual therapy as an isolated intervention was added to exercise or usual care. |
| Romeo | 2024 | Physical therapy in addition to occlusal splint in myogenic temporomandibular disorders: A randomised controlled trial | Study design not eligible for this systematic review, since not manual therapy as an isolated intervention was added to exercise or usual care. |
| Tan | 2024 | Effectiveness of focused extracorporeal shock wave versus manual therapy in postpartum patients with sacroiliac joint dysfunction: a prospective clinical trial | Study design not eligible for this systematic review, since not manual therapy as an isolated intervention was added to exercise or usual care. |
|  | | | |
