# Supplementary material for: Evaluating Manual Therapy in Musculoskeletal Pain: Why Certain Trial Designs May Overestimate Effectiveness—A Scoping Review
Source: Eur J Pain. 2025 Nov 13;29(10):e70150. doi: 10.1002/ejp.70150 (PMC12614156; doi:10.1002/ejp.70150)
Supplement: Supplementary file 3 — Data S3: ejp70150‐sup‐0003‐DataS3.docx. [file EJP-29-0-s002.docx]

| **Supplementary Table 1:** Joanna Brigg’s Institute critical appraisal tool for randomized controlled trials | | | | | | | | | | | | | | |  |
| --- | --- | --- | --- | --- | --- | --- | --- | --- | --- | --- | --- | --- | --- | --- | --- |
| First author, year | 1 | 2 | 3 | 4 | 5 | 6 | 7 | 8 | 9 | 10 | 11 | 12 | 13 | Total points | |
| Abbott, 2013 | 1 | 1 | 0 | 0 | 0 | 1 | 1 | 1 | 1 | 1 | 1 | 1 | 1 | 10 | |
| Akgüller, 2014 | 1 | 1 | 1 | 0 | 1 | 1 | 1 | 1 | 1 | 1 | 1 | 1 | 1 | 12 | |
| Al-Banawi, 2023 | 1 | 0 | 1 | 0 | 0 | 1 | 1 | 1 | 1 | 1 | 1 | 1 | 1 | 10 | |
| Abbott, 2015 | 1 | 1 | 1 | 0 | 0 | 1 | 1 | 1 | 1 | 1 | 1 | 1 | 1 | 11 | |
| Akhter, 2014 | 1 | uc | 1 | 0 | 0 | uc | uc | 1 | uc | 1 | 1 | uc | 1 | 6 | |
| Ali, 2015 | 1 | uc | 1 | 0 | 0 | uc | uc | 1 | uc | 1 | 1 | 1 | 1 | 7 | |
| Azlin, 2011 | uc | uc | 1 | 0 | 0 | 1 | uc | 1 | uc | 1 | 1 | 1 | 1 | 7 | |
| Bakken, 2021 | 1 | 1 | 1 | 1 | 0 | 1 | 1 | 1 | 1 | 1 | 1 | 1 | 1 | 12 | |
| Bang, 2020 | 1 | uc | 1 | 0 | 0 | 1 | 1 | 1 | uc | 1 | 1 | 1 | 1 | 9 | |
| Barobsa, 2008 | uc | uc | 0 | uc | 0 | 0 | 1 | 1 | uc | 1 | 1 | 1 | 1 | 6 | |
| Blackman, 2014 | 1 | 1 | 0 | 0 | 0 | uc | 0 | 1 | 0 | 1 | 1 | 1 | 1 | 7 | |
| Bolton, 2020 | 1 | 0 | 1 | 0 | 0 | 1 | 1 | 1 | uc | 1 | 1 | 1 | 1 | 9 | |
| Bronfort, 2001 | 1 | 1 | 1 | 0 | 0 | 1 | 0 | 1 | 1 | 1 | 1 | 1 | 1 | 10 | |
| Bronfort, 2014 | 1 | 1 | 1 | 0 | 0 | 1 | 1 | 1 | 1 | 1 | 1 | 1 | 1 | 11 | |
| Camargo, 2015 | 1 | 1 | 1 | 0 | 0 | 0 | 1 | 1 | 1 | 1 | 1 | 1 | 1 | 10 | |
| Celenay, 2016 | 1 | 1 | 1 | 0 | 0 | 0 | 1 | 1 | 1 | 1 | 1 | 1 | 1 | 10 | |
| Celik, 2015 | 1 | 1 | 1 | 0 | 0 | 0 | 1 | 1 | uc | 1 | 1 | 1 | 1 | 9 | |
| Ceylan, 2023 | 1 | uc | 1 | 0 | 0 | 1 | 1 | 1 | uc | 1 | 1 | 1 | 1 | 9 | |
| Chen, 2009 | 1 | 1 | 1 | 0 | 0 | 1 | 1 | 1 | 1 | 1 | 1 | 1 | 1 | 11 | |
| Conroy, 1998 | 0 | 0 | 1 | 0 | 0 | 1 | 1 | 1 | uc | 1 | 1 | 1 | 1 | 8 | |
| Cook, 2014 | 1 | uc | 1 | 0 | 0 | 1 | 1 | 1 | 1 | 1 | 1 | 1 | 1 | 10 | |
| Copurgensli, 2016 | 1 | 1 | 1 | 0 | 0 | 0 | 1 | 1 | uc | 1 | 1 | 1 | 1 | 9 | |
| Corum, 2018 | uc | 0 | 1 | 0 | 0 | 1 | 1 | uc | uc | 1 | 1 | 1 | 1 | 7 | |
| Delgado de la Serna, 2019 | 1 | 1 | 1 | 0 | 0 | 1 | 1 | 1 | 1 | 1 | 1 | 1 | 1 | 11 | |
| Duymaz, 2018 | uc | 0 | 1 | 0 | 0 | 0 | 0 | 1 | uc | 1 | 1 | 1 | 1 | 6 | |
| Dwyer, 2015 | 1 | 1 | 1 | 0 | 0 | 1 | 1 | 1 | 1 | 1 | 1 | 1 | 1 | 11 | |
| Dziedzic, 2005 | 1 | 1 | 1 | 0 | 0 | 1 | 1 | 1 | 1 | 1 | 1 | 1 | 1 | 11 | |
| Eldesoky, 2019 | 1 | uc | 1 | 0 | 0 | 0 | 1 | 1 | uc | 1 | 1 | 1 | 1 | 8 | |
| Eliason, 2021 | 1 | 1 | 1 | 0 | 0 | 1 | 1 | 1 | 1 | 1 | 1 | 1 | 1 | 11 | |
| Evans, 2012 | 1 | 1 | 1 | 0 | 0 | 1 | 1 | 1 | 1 | 1 | 1 | 1 | 1 | 11 | |
| Farooq, 2018 | 1 | 1 | 1 | 1 | 0 | 1 | 1 | 1 | uc | 1 | 1 | 1 | 1 | 11 | |
| Fathollahnejad, 2019 | 1 | 1 | 1 | 0 | 0 | 1 | 1 | 1 | uc | 1 | 1 | 1 | 1 | 10 | |
| Fitzgerald, 2016 | 1 | 1 | 1 | 0 | 0 | 1 | 1 | 1 | 1 | 1 | 1 | 1 | 1 | 11 | |
| French, 2013 | 1 | 1 | 1 | 0 | 0 | 1 | 1 | 1 | 1 | 1 | 1 | 1 | 1 | 11 | |
| Ganesh, 2015 | 1 | 1 | 1 | 0 | 0 | 1 | 1 | 0 | uc | 1 | 1 | 1 | 1 | 9 | |
| Gonzalez-Iglesias, 2009a | 1 | 1 | 1 | 1 | 0 | 1 | 1 | 1 | 1 | 1 | 1 | 1 | 1 | 12 | |
| Gonzalez-Iglesias, 2009b | 1 | 1 | 1 | 1 | 0 | 1 | 1 | 1 | 1 | 1 | 1 | 1 | 1 | 12 | |
| Gonzalez-Rueda, | 1 | 1 | 1 | 0 | 0 | 1 | 1 | 1 | 1 | 1 | 1 | 1 | 1 | 11 | |
| Grunnesjo, 2004 | 1 | 1 | 1 | 0 | 0 | 1 | 0 | 1 | 1 | 1 | 1 | 1 | 1 | 10 | |
| Gutierrez-Espinosa, 2023 | 1 | 1 | 1 | 0 | 0 | 1 | 1 | 1 | 1 | 1 | 1 | 1 | 1 | 11 | |
| Haider, 2018 | 1 | uc | 1 | 0 | 0 | uc | 1 | 1 | 1 | 1 | 1 | 1 | 1 | 9 | |
| Hoving, 2006 | 1 | 1 | 1 | 0 | 0 | 1 | uc | 1 | 1 | 1 | 1 | 1 | 1 | 10 | |
| Javadov, 2021 | 1 | uc | 1 | 0 | 0 | uc | uc | 1 | uc | 1 | 1 | 1 | 1 | 7 | |
| Joshi, 2014 | uc | uc | uc | 0 | 0 | uc | uc | 1 | uc | 1 | 1 | 0 | 0 | 3 | |
| Jull, 2002 | 1 | 1 | 1 | 0 | 0 | 1 | 1 | 1 | 1 | 1 | 1 | 1 | 1 | 11 | |
| Just, 2009 | 1 | uc | 0 | 0 | 0 | 1 | uc | 1 | uc | 1 | 1 | 1 | 1 | 7 | |
| Kachingwe, 2008 | 1 | 1 | 1 | 1 | 0 | 1 | 1 | uc | uc | 1 | 1 | uc | 1 | 10 | |
| Khan, 2016 | 1 | 0 | uc | 0 | 0 | uc | uc | uc | uc | 1 | 1 | 1 | 1 | 5 | |
| Kromer, 2013 | 1 | 1 | 1 | 0 | 0 | 1 | 1 | 1 | 1 | 1 | 1 | 1 | 1 | 11 | |
| Kuklarni, 2016 | uc | uc | uc | 0 | 0 | 0 | uc | 1 | uc | 1 | 1 | 0 | 0 | 3 | |
| Lalnupuii, 2016 | 1 | uc | 1 | 1 | 0 | 1 | uc | 1 | uc | 1 | 1 | 0 | 1 | 8 | |
| Lau, 2011 | 1 | 1 | 1 | 0 | 0 | 1 | 1 | 1 | 1 | 1 | 1 | 1 | 1 | 11 | |
| Lee, 2016 | 1 | uc | 1 | 0 | 0 | 1 | 1 | 1 | uc | 1 | 1 | 1 | 1 | 9 | |
| Maiers, 2014 | 1 | 1 | 1 | 0 | 0 | 1 | 1 | 1 | 1 | 1 | 1 | 1 | 1 | 11 | |
| Menek, 2019 | 1 | uc | 1 | 0 | 0 | 0 | 1 | 0 | uc | 1 | 1 | 1 | 1 | 7 | |
| Michener, 2024 | 1 | 1 | 1 | 1 | 0 | uc | 1 | 1 | 1 | 1 | 1 | 1 | 1 | 11 | |
| Mintken, 2016 | 1 | 1 | 1 | 0 | 0 | 1 | 1 | 1 | 1 | 1 | 1 | 1 | 1 | 11 | |
| Mostamad, 2023 | 1 | 0 | 1 | 0 | 0 | 0 | 1 | 1 | 1 | 1 | 1 | 1 | 1 | 9 | |
| Murphy, 2010 | 1 | 0 | 1 | 0 | 0 | 0 | 1 | 1 | uc | 1 | 1 | 1 | 1 | 8 | |
| Nagata, 2019 | 1 | 1 | 1 | 1 | 0 | 0 | 1 | 1 | 1 | 1 | 1 | 1 | 1 | 11 | |
| Narang, 2014 | uc | 0 | 1 | 0 | 0 | 0 | uc | 1 | uc | 1 | 1 | 0 | 0 | 4 | |
| Naranjo-cinto, 2022 | 1 | 1 | 1 | 1 | 0 | 1 | 1 | 1 | 1 | 1 | 1 | 1 | 1 | 12 | |
| Nigam, 2020 | 1 | 1 | 1 | 0 | 0 | 1 | 1 | 1 | 1 | 1 | 1 | 1 | 1 | 11 | |
| Ojo Ojawo, 2016 | 1 | 1 | 1 | 0 | 0 | 0 | 1 | 0 | uc | 1 | 1 | 0 | 0 | 6 | |
| Ojo Ojawo, 2018 | 1 | 1 | 1 | 0 | 0 | 0 | 1 | 1 | 1 | 1 | 1 | 1 | 1 | 10 | |
| Poulsen, 2013 | 1 | 1 | 1 | 1 | 0 | 0 | 1 | 1 | 1 | 1 | 1 | 1 | 1 | 11 | |
| Rasmsusen, 2018 | 1 | 1 | 1 | 1 | 0 | 1 | 1 | 1 | 1 | 1 | 1 | 1 | 1 | 12 | |
| Reynolds, 2020 | 1 | 1 | 1 | 1 | 0 | 1 | 1 | 1 | 1 | 1 | 1 | 1 | 1 | 12 | |
| Rodriguez-Sanz, 2020 | 1 | 1 | 1 | 0 | 0 | 1 | 1 | 1 | 1 | 1 | 1 | 1 | 1 | 11 | |
| Sai, 2015 | 1 | 1 | 1 | 0 | 0 | 1 | 1 | 0 | 1 | 1 | 1 | 1 | 1 | 10 | |
| Satpute, 2015 | 1 | 1 | 1 | 1 | 0 | 1 | 1 | 1 | 1 | 1 | 1 | 1 | 1 | 12 | |
| Schulz, 2019 | 1 | 1 | 1 | 0 | 0 | 1 | 1 | 1 | 1 | 1 | 1 | 1 | 1 | 11 | |
| Waqas, 2023 | 1 | 1 | 1 | 1 | 0 | 1 | 1 | 1 | 1 | 1 | 1 | 1 | 1 | 12 | |
| Shenouda, 2014 | uc | uc | 1 | 0 | 0 | 0 | 1 | uc | uc | 1 | 1 | 1 | 1 | 6 | |
| Subhash, 2020 | uc | uc | 1 | 0 | 0 | 0 | uc | uc | uc | 1 | 1 | uc | uc | 3 | |
| Tauqeer, 2024 | 1 | 1 | 1 | 0 | 0 | 1 | 1 | 1 | 1 | 1 | 1 | 1 | 1 | 11 | |
| Tuncer, 2013 | 1 | 1 | 1 | 0 | 0 | 1 | 1 | 1 | uc | 1 | 1 | 1 | 1 | 10 | |
| Ughreja, 2017 | 1 | uc | uc | 0 | 0 | 0 | uc | 1 | 1 | 1 | 1 | 1 | 1 | 7 | |
| UK Beam Team, 2004 | 1 | uc | 1 | 0 | 0 | 0 | 1 | 1 | 1 | 1 | 1 | 1 | 1 | 9 | |
| Yang, 2015 | uc | uc | 1 | 0 | 0 | 0 | uc | 1 | uc | 1 | 1 | 1 | 1 | 6 | |
| Yiasemides, 2011 | 1 | 1 | 1 | 0 | 0 | 1 | 1 | 1 | 1 | 1 | 1 | 1 | 1 | 11 | |
| Dogan, 2021 | 1 | uc | 1 | 0 | 0 | 0 | 1 | 1 | uc | 1 | 1 | uc | 1 | 7 | |
| Nam, 2013 | uc | uc | 1 | 0 | 0 | 0 | 1 | 1 | uc | 1 | 1 | uc | 1 | 6 | |
| Espi-Lopez, 2020 | 1 | 1 | 1 | 0 | 0 | 1 | 1 | 1 | 1 | 1 | 1 | uc | uc | 9 | |
| Park, 2020 | 1 | uc | 1 | 0 | 0 | 1 | 1 | 1 | 1 | 1 | 1 | 1 | 1 | 10 | |
| Rezaie, 2021 | 1 | 1 | 1 | 1 | 0 | 1 | 1 | 1 | uc | 1 | 1 | 1 | 1 | 11 | |
| Rodriguez-Sanz, 2022 | 1 | 1 | 1 | 0 | 0 | 1 | 1 | 1 | uc | 1 | 1 | 1 | 1 | 10 | |
| Childs, 2004 | 1 | 1 | 1 | 0 | 0 | 1 | 1 | 1 | 1 | 1 | 1 | 1 | 1 | 11 | |
| Jüni, 2009 | 1 | 1 | 1 | 0 | 0 | 1 | 1 | 1 | 1 | 1 | 1 | 1 | 1 | 11 | |
| Hancock, 2007 | 1 | 1 | 1 | 1 | 0 | 1 | 1 | 1 | 1 | 1 | 1 | 1 | 1 | 12 | |
| Hallegraeff, 2009 | 1 | 1 | 1 | 0 | 0 | 0 | 1 | 1 | uc | 1 | 1 | 1 | 1 | 9 | |
| Satpute, 2019 | 1 | 1 | 1 | 1 | 0 | 1 | 1 | 1 | 1 | 1 | 1 | 1 | 1 | 12 | |
| Bergman, 2004 | 1 | 1 | 1 | 0 | 0 | 1 | 1 | 1 | 1 | 1 | 1 | 1 | 1 | 11 | |
| Nejati, 2019 | 1 | uc | 1 | 0 | 0 | 1 | 1 | 1 | uc | 1 | 1 | 1 | 1 | 9 | |
| Lytras, 2023 | 1 | 1 | 1 | 0 | 0 | 1 | 1 | 1 | 1 | 1 | 1 | 1 | 1 | 11 | |
| 1 = Was true randomization used for assignment of participants to treatment groups?  2 = Was allocation to treatment groups concealed?  3 = Were treatment groups similar at baseline?  4 = Were participants blind to treatment assignment?  5 = Were those delivering the treatment blind to treatment assignment?  6 = Were outcome assessors blind to treatment assignment?  7 =Were treatment groups treated identically other than the intervention of interest?  8 = Was follow up complete and if not, were differences between groups […] adequately described and analysed?  9 = Were participants analysed in the groups to which they were randomized?  10 = Were outcomes measured in the same way for treatment groups?  11 = Were outcomes measured in a reliable way?  12 = Was appropriate statistical analysis used?  13 = Was the trial design appropriate, and any deviations from the standard RCT design […] accounted for in the conduct and analysis of the trial? | | | | | | | | | | | | | | |  |

| **Supplementary Table 2:** Description of treatment in the intervention and control groups with the provided manual therapy definition and delivery mode | | | | |
| --- | --- | --- | --- | --- |
| First author, year | Treatment in Intervention group (IG) | Treatment in Control group (CG) | Manual therapy delivery | Adverse events (AE) |
| Abbott, 2013 | Treatment of CG in addition low-velocity low-amplitude (LVLA)-techniques and soft-tissue as seemed fit in each individual case.  *LVLA, concept not specified* | Multimodal supervised exercise program with warm-up, muscle strengthening, stretching, with individualized exercises. | MT was provided within, not in addition to CG treatment | No serious AE |
| Abbott, 2015 | Treatment of CG in addition to LVLA-techniques and soft-tissue as seemed fit in each individual case.  *LVLA, concept not specified* | Multimodal supervised exercise program with warm-up, muscle strengthening, stretching, with individualized exercises. | MT (45 min.) was provided in addition to exercise (45 min.) | One non-serious fall on the knee during one exercise session. |
| Akgüller, 2024 | High-velocity low-amplitude (HVLA) techniques of the cervical spine (C2-C7) with up to two attempts.  *HVLA, concept not specified* | Stabilization and strengthening exercises for arms, legs, and isometric cervical exercises. | MT (10 min.) was provided in addition to exercise (30 min). | No serious AE |
| Akhter, 2014 | HVLA-techniques of Maitland rotation/ lateral flexion techniques on painful and stiff cervical segments in supine position.  *Maitland HVLA* | Isometric, eccentric and concentric cervical movement exercises, stretching (10 repetitions). | MT (of unclear duration) was provided in addition to exercise (20 min.). | AE were not reported |
| Al-Banawi, 2023 | McKenzie Exercises, TENS, and education for 35-45 minutes. Grade I-II LVLA Maitland in first three sessions, Grade III-IV for last three.  *Maitland LVLA* | McKenzie Exercises, TENS, and education. | MT (15 minutes) was provided in addition to McKenzie, TENS, and education (35-45 minutes). | No serious AE |
| Ali, 2015 | General shoulder exercises, home program, grade II-III posterior-anterior and inferior-caudal Maitland glides LVLA-techniques (5x2-3 oscillations/second for 30 seconds)  *Maitland LVLA* | General shoulder exercises and home program. | MT was provided within, not in addition to CG treatment (45 minutes). | AE were not reported |
| Azlin, 2011 | Isometric exercises, lower limb stretches, closed kinetic chain exercises and static bicycling with heat pack. Additionally, Maitland LVLA-techniques for the knee and patella glides.  *Maitland LVLA* | Isometric exercises, lower limb stretches, closed kinetic chain exercises and static bicycling with heat pack. | MT (of unclear duration) delivery mode was not specified. | No serious AE |
| Bakken, 2021 | Spinal HVLA and LVLA-techniques in addition to a home stretching program.  *LVLA & HVLA, concept not specified* | Home stretching program, not further specified, neither content, nor the duration. | MT (of unclear duration) was provided in addition to a home stretching program (of unclear duration). | No serious AE, however, three non-serious AE in intervention, 1 in control group. |
| Bang, 2000 | Strengthening exercises and stretching in addition to Grade I-Grade V Maitland LVLA- and HVLA-techniques.  *Maitland LVLA & HVLA* | Strengthening exercises and stretching | MT (of unclear duration) was provided within, not in addition to CG treatment. | AE were not reported |
| Barbosa, 2008 | LVLA-techniques for shoulder joint and sternoclavicular joint in addition to ultrasound, in addition to treatment of CG.  *Maitland LVLA* | 3x20 eccentric repetitions for empty can abduction and biceps curls as exercise training, | MT (of unclear duration) delivery mode was not specified. | AE were not reported |
| Bergman, 2004 | All interventions of the CG with the addition of LVLA- and HVLA-techniques for cervical and thoracic spine and adjacent ribs. The practitioner based upon the examination findings made treatment choice.  *LVLA & HVLA, concept not specified* | Usual care, which consisted of oral pain medication, advice and reassurance, corticosteroid, and anaesthetic injections and eventually physiotherapy with exercises, massage, physical modalities, was considered. | MT (23 minutes) was delivered provided in addition to usual medical care. | AE were not reported |
| Blackman, 2014 | Grade b LVLA- and HVLA-techniques of the hip into flexion, abduction, extension (4x 60 seconds for each, 4/week) and same exercises as control group.  *Maitland LVLA & HVLA* | sit-to stand, mini squats, active hip extension and active hip abduction in standing, and bridging in supine 3x10 repetitions 4/week. | MT (of unclear duration) was provided in addition to a home exercise program. | AE were not reported |
| Bolton, 2020 | HVLA-techniques for proximal and distal tibiofibular joint prior exercises in supervised balance exercise at the first three sessions.  *HVLA, concept not specified* | Supervised balance exercises. | MT (of unclear duration) was provided in addition to supervised exercises. | AE were not reported |
| Bronfort, 2001 | HVLA-techniques in the cervical and thoracic spine (15 minutes) and some soft tissue techniques, no physical therapies. Followed by low-technique rehabilitative exercises (45 minutes).  *HVLA, concept not specified* | Aerobic exercises with a stationary bike (15-20 minutes), dynamic progressive exercises on MedX cervical extension and rotation machine with approximately twenty repetitions per set. | In the IGMT was added (15 min + 45 min) to exercise therapy. However, to balance for time and attention all participants attended twenty-one-hour sessions. | Notable increase in headache pain (8 in intervention, eight in control group). Other side effects were increased radicular pain in one patient in the intervention group. |
| Bronfort, 2014 | LVLA- and HVLA-techniques of lumbar vertebral or sacroiliac joints determined by individual presentation. Soft-tissue techniques, compression, physical therapy, and home exercise program as the CG. The clinician depending on the presentation and physical examination chose the specific technique.  LVLA & HVLA, concept not specified | Four one-hour visits with advice and home-exercise instructions. Stabilization exercises with flexion, extension patterns with 3x25 reps. 8-12 reps of pelvic tilt, quadruped every other day in standing, lying or seated position, bridging, abdominal curl-ups, side-bridging. | MT (10-20 minutes) was provided in addition to a home exercise program (45 minutes) | 5 SAE, all were unrelated to the study intervention (bowel obstruction, anaphylaxis, menorrhagia, trauma). Moderate self-limiting AE 30% of intervention and 42% of control group. |
| Camargo, 2015 | Grade III-IV Maitland LVLA-techniques for glenohumeral, scapulothoracic, acromioclavicular and sternoclavicular joints and cervical spine, soft tissue techniques, neuromuscular facilitation, rhythmic stabilization, contract relax techniques. The specific technique was chosen according to the individual presentation.  *Maitland LVLA* | 3 Stretching and three strengthening exercises supervised by PT (3x30sec holding stretches, elastic band resistance exercises for ERO, trapezius and serratus anterior exercises (pain free maximal range of motion). | MT (45 minutes) was provided in addition to supervised exercise program. | AE were not reported. |
| Celenay, 2016 | Cyriax and Kaltenborn LVLA-techniques (traction, gliding, and scapular mobilization) as patients required it in addition to the treatment of the control group.  *Kaltenborn & Cyriax LVLA* | 10-minute warm-up, 40 minutes stabilization exercises, 10-minute cool-down and stretching routine on 3 days per week. Further participants received postural education and deep neck flexion exercises were taught. | MT (15-20 minutes) was provided in addition to supervised exercise program (60 minutes). | No serious AE reported. Self-limiting soreness after one session (24-48 hours). |
| Celik, 2015 | LVLA-techniques grades I-IV for the glenohumeral joint in addition to the treatment of the control group.  Maitland LVLA | 20 seconds cyclic stretching exercise and sleepers stretch for the shoulder. Home exercise program with ten reps for all shoulder movement directions each and a cold pack. | MT (30 minutes) was provided in addition to supervised stretching (20 minutes). | AE were not reported. |
| Ceylan, 2023 | Movement with mobilization (MWM) from Mulligan depending on the individual presentation with three-by-ten reps for each movement in addition to the treatment of the control group.  *Mulligan MWM* | Electrotherapy with TENS (20 minutes), ultrasound (6 minutes), tendon-gliding exercises 3x10 reps per day, orthosis for 23 hours per day, strengthening exercises 3x10 reps per day, median nerve gliding exercise 5x5 seconds daily. | MT (of unclear duration) was provided in addition to exercise, neurodynamics and physical therapies (50-60 minutes). | AE were not reported. |
| Chen, 2009 | LVLA Maitland techniques Grades I-IV without thrusts in addition to the treatment of the control group.  *Maitland LVLA* | Neuromuscular exercises of the shoulder region in a pain-free manner tailored to the individual patient and participants were directed to perform exercises twice daily at least. | MT (of unclear duration) was provided in addition to neurodynamic shoulder exercises (30 minutes). | AE were not reported. |
| Childs, 2004 | HVLA-technique during the first two physiotherapy sessions and exercises, as the CG received.  *Maitland HVLA* | Progressively designed aerobic exercise, starting with 10 minutes of stationary bike or treadmill at self-selected pace and lumbar strengthening program. | MT (of unclear duration) was provided in addition to exercise. | No serious AE. |
| Conroy, 1998 | LVLA Maitland techniques to the glenohumeral joint with a maximum of 15 additional treatments compared to the control group. Other treatment was the same as in the control group.  *Maitland LVLA* | Hot pack, range-of-motion exercises, stretching, strengthening, and soft-tissue mobilization and patient education. | MT (of unclear duration) was provided in addition to exercise, education, soft-tissue mobilization, and hot pack. | AE were not reported. |
| Cook, 2014 | LVLA Maitland techniques to the cervical spine grade III posterior-anterior mobilisations in prone for 3x30 repetitions, tailored to everyone. Other than that, participants also received treatment from the control group.  *Maitland LVLA* | Tailored pragmatic evidence-based physiotherapy, dosage and interventions were specific to examination findings with manual therapy, self- and externally applied stretching, isotonic strengthening, restoration of movement for the shoulder. | MT (of unclear duration) provision mode is unclear. | No serious AE. |
| Copurgensli, 2014 | MWM Mulligan, NAGs and SNAGs from C2-C7 in addition to the treatment of the control group.  *Mulligan SNAGs & NAGs* | TENS (20 minutes), hot pack (15 minutes), exercises. | MT (of unclear duration) was provided in addition to exercise and physical therapies. | AE were not reported. |
| Corum, 2018 | HVLA-technique according to the manual examination of each patient. After each manipulation or sham manipulation patients were supervised for their exercises. In addition to the treatment of the control group.  *HVLA, concept not specified* | Sham manipulation, as in a HVLA to the cervicothoracic junction to not influence the upper cervical spine. Patients were instructed to perform a home exercise program 3/ week with 3x5 repetitions (30-60 second breaks in between), neck stretching exercises and upper body strengthening exercises. | MT (of unclear duration) was provided as part of a comprehensive physical therapy program and controlled for with a sham intervention. | Two patients in the IG dropped out of the study because of AE (headache, dizziness) while one patient in the CG discontinued treatment. |
| Delgado de la Serna, 2020 | Manual therapy techniques to the TMJ, self-massage and massage of masseter and temporal muscles as well as 90 seconds of oscillatory and glide mobilizations. In addition to the treatment of the control group.  *LVLA, concept not specified* | Temporomandibular joint (TMJ) exercises, tongue movements, advice for posture and jaw / head position was provided. | MT (of unclear duration) was provided in addition to exercise, self-massage, and advice. | No serious AE. |
| Dogan, 2021 | Three separate LVLA-techniques were applied to patients SI-joint (Stoddart cross technique, Selling technique, Maigne technique). Dosage or other parameters were not specified further. In addition to the treatment of the control group.  *LVLA, concept not specified* | Patients were instructed to perform a home exercise and stretching program: hamstring stretches, hip adductor stretch, piriformis stretch, quadriceps stretch, one knee to chest stretch, both knees to chest stretch, lower trunk rotation, and pelvic rotation stretch. Strengthening exercises were assigned after stretching exercises. Isometric hip abduction/adduction strengthening and prone position lumbar/hip strengthening exercises were given as strengthening exercises. 5 repetitions for each, two sessions daily. | MT (of unclear duration) was provided in addition to home exercise and stretching program. | AE were not reported. |
| Duymaz, 2018 | Three sets of mulligan mobilization techniques with overpressure on the cervical facet joints applied by the therapist or the patient with 3x 10 repetitions, 15-20 seconds break in between. Patients were instructed to self-mobilize 3x10 repetitions three times a day at home.  *Mulligan MWM* | The CG received a home exercise program with range of motion exercises and stretching including neck flexion, extension, right/left lateral flexion along with stretching exercises for upper trapezius, posterior part of deltoid and pectoral muscles to be practiced three times a day with ten repetitions. | MT (of unclear duration) was provided in addition to a home exercise and stretching program, which naturally consisted of other exercises in the CG than mulligan movements. | No serious AE. |
| Dwyer, 2015 | Patient education about the diagnosis and prognosis was provided, alongside lifestyle advice. LVLA- and HVLA-techniques were applied to the supposedly affected kinematic chain (knee, hip, foot, and spine). Further soft tissue treatment was applied as deemed necessary. 2-6 sets of 30 manual repetitions were applied in addition to the treatment of the control group.  *Maitland LVLA & HVLA* | Patient education, exercise prescription, soft tissue treatment, and passive stretches (on treatment days only) to the knee and elsewhere along the full kinetic chain, where needed, based on functional assessment. Education consisted of information about the diagnosis and prognosis, and advice on health promotion and lifestyle. The content and timing of treatment were important in that advice, education, and training were provided to participants at the onset of their treatment program (week 1) and reinforced at 2 other points during the treatment period (weeks 2 and 4). Home exercise program with reinforcements in later sessions. No manual joint (LVLA, HVLA) techniques were applied. | MT (3x20 minutes extra) was provided in addition to a comprehensive physical therapy program with education, advice, exercises, home exercises, and lifestyle interventions. | No serious AE. |
| Dziedzic, 2005 | LVLA- and HVLA-techniques and soft tissue techniques graded as appropriate to the patients’ signs and symptoms judged by the treating therapist. In addition to the treatment of the control group.  *LVLA & HVLA, concept not specified* | Individualized education, advice, and a home exercise program with active and resisted neck movements. Dosage and frequency were not further specified. | MT (of unclear duration) was included in the treatment time which was 20 minutes in total. | No serious AE. |
| Eldesoky, 2019 | LVLA Maitland oscillatory posterior-anterior and rotational techniques were applied with 2-3 movements at each spinal level for 10 repetitions with 30 seconds for each technique. In addition to the treatment of the control group.  *Maitland LVLA* | Both groups received the same ultrasound and exercise program. 5 min ultrasound 3/week for 4 weeks on the neck, stretching and strengthening. The program included stretching exercise for scalene, upper trapezius, levator scapulae, sternocleidomastoid, and pectoralis major muscles. | MT (of unclear duration) was provided in addition to ultrasound, exercise and strengthening program. | No serious AE. |
| Eliason, 2021 | LVLA Kaltenborn techniques and traction techniques during the first 6 weeks added to the exercises and home exercise program. Lateral, dorsal and ventral mobilization of the humeral head was performed for 30 seconds, 3 times. In addition to the treatment of the control group.  *Kaltenborn LVLA* | 3x10 sets of exercises while allowing some pain (NRS 1-4/10). Each exercise was held 2-5 seconds, 3x10 sets with scapula retraction, rowing, abduction, adduction, external rotation, shoulder shrugs, trapezius stretching and pectoralis major stretching. Exercise intensity was increased gradually with elastic bands and dumbbells according to pain and exhaustion on an individual basis. Stretching exercises were held for 20-30 seconds, two repetitions. Both groups also performed a home exercise program twice daily, on days with supervised exercise once daily. | MT (of unclear duration) was provided in addition to a supervised strengthening and stretching program as well as a home exercise program. | No serious AE. |
| Espi-Lopez, 2020 | Combination of ten different manual and myofascial techniques applied on the cervical, suboccipital, and temporomandibular areas with LVLA on C7 vertebra, posterior anterior LVLA-techniques on C5 vertebra, mobilization upper neck, suboccipital inhibition technique for 2 minutes, suboccipital mobilization technique with occipito-atlo-axoidea thrust (HVLA) with a maximum of two attempts to achieve a cavitation, trigger points on masseter, temporal and sternocleidomastoid muscles, myofascial technique on pterygoid lateral, medial and masseter muscle, TMJ mobilization technique, and temporomandibular massage. In addition to the treatment of the control group.  *LVLA & HVLA, concept not specified* | Both groups used a personalized occlusal splint which patients were instructed to wear for 12h a day for the duration of the study. | MT (45 minutes, 4 appointments each) was provided in addition to occlusal splint therapy. | AE were not reported. |
| Evans, 2012 | Spinal manipulative therapy after a clinician having identified the supposedly aberrantly moving spinal segments through static and dynamic spinal palpation to the cervical and thoracic spine using LVLA- and HVLA-techniques and up to five minutes of soft tissue massage to facilitate treatment. In addition to the treatment of the control group.  *LVLA & HVLA, concept not specified* | Neck and upper body exercises supervised high intensity sessions with individualized intensity and load increase. Focus was cervical strengthening exercises with patients wearing headgear with variable weight attachments (1.25-10 lbs.’) guided by a pulley system with 3x15-25 repetitions of dynamic flexion, extension, rotation exercises performed and upper body exercises with push-ups, dumbbell shoulder and chest exercises in addition to a light aerobic warm-up and stretching before and after working out. | MT (15-20 minutes) was provided in addition to supervised exercise therapy (60 minutes). | Only mild and transient adverse events. |
| Farooq, 2018 | LVLA Maitland techniques with posterior-anterior oscillatory mobilization were provided in the prone lying position.  The grade, duration, and repetitions of the mobilization were determined by the treating therapist, based on his decision making and clinical reasoning skills, to meet the patient's requirement following the dosage described by  clinical experience of more than five years. In addition to the treatment of the control group.  *Maitland LVLA* | Patients received routine PT, which comprised patient education, a home exercise plan, an educational pamphlet with a standard set of neck exercises and general advice on posture correction. The home exercise program included stretching exercises for levator scapulae, upper trapezius, scalene muscles and active neck mobility and isometric exercises. For stretching patients were instructed to maintain end of range for 2x30 seconds. Neck ROM exercises each movement 10 times. Isometric exercises against pressure of their head against resistance of their hand/ fingers for 5x 10 seconds daily. In addition, they received superficial thermal therapy over the painful area for 15 minutes (250W infrared lamp), ultrasound therapy in continuous mode (1.5W/cm2 for 10 minutes), high frequency TENS for 20 minutes. | MT (of unclear duration) was provided in addition to a home exercise program, an educational pamphlet, TENS, ultrasound, thermal therapy, stretching and exercise program. | AE were not reported |
| Fathollahnejad, 2019 | HVLA-techniques for 10 minutes according to the manual passive examination to check for ROM restrictions of the cervical spine. In addition to the treatment of the control group.  *HVLA, concept not specified* | Patients were instructed on how to perform the exercises and received postural advice. 5-minute walking as warm-up, strengthening exercises targeting periscapular muscles 3x10-15 repetitions. In addition, patients performed stretching to increase flexibility of the pectoralis muscle and the cervical neck extensors. | MT (of unclear duration) was provided in addition to a supervised exercise program. | AE were not reported |
| Fitzgerald, 2016 | LVLA- and HVLA-techniques addressing knee flexibility, soft tissue manipulations of lower leg muscles and additional optional MT techniques for the hip, foot and ankles as required by the individuals. 3x30 oscillations were applied with grade III-V mobilizations and a maximum of 6 sets. Further manual stretches to quadriceps, hamstrings, gastrocnemius three times (1x 60 seconds, 2x30 seconds, 3x20 seconds) were performed and soft tissue mobilization and 10-30x physiological movements with 3-6 sets according to the therapist. In addition to the treatment of the control group.  *LVLA & HVLA, concept not specified* | 10 Minute aerobic warm-up with treadmill walk or stationary cycling. Strengthening, stretching and neuromuscular control with additional exercises based on the examination findings which aim to address flexibility as well. Examples constitute leg extensions, partial squats and squats, step-ups and step-downs, bridging variations, prone hamstring curls, bilateral calf raises and unilateral lowering. Hip abductions exercise as well. Strengthening exercises were 3x10 repetitions with a 10 second isometric holding phase. 1 Minute stretching must be performed at each setting. 3x2 minutes with challenging exercise. | MT (15-20 minutes) was provided in addition to strengthening and stretching exercises. | Two participants had mild hip and knee stiffness and more pain after the first treatment. Three other patients experienced non-project related adverse events. |
| French, 2013 | Individuals were treated with LVLA-techniques based on pain and stiffness of the individual with no more than 5 different MT techniques allowed during an individual session. Available techniques were posterior anterior Maitland techniques, MWM mulligan techniques grade I-III. Physiotherapists were instructed to use 2-5 techniques during one appointment. In addition to this, the participants received the same treatment as the control group.  *Maitland LVLA & Mulligan MWM* | Flexibility and strengthening exercises within a semi-structured protocol, which was individually, progressed according to the individual patient assessment findings. Strengthening focussed on low-load exercise with non-weight-bearing positions and progressing to functional positions with key target muscles as the gluteal muscles. 5 minute warm up on a stationary bike, 5x 30 second stretching exercises 3 times per day of hip extensors, abductors, hamstrings, external and internal rotation. Strengthening exercises were mostly isometric bridging, gluteus medius exercises in standing and side lying as well as wall-squats, step-ups, step-downs, and lunges with body weight. Further a home exercise program was implemented and advice to undertake aerobic exercise like cycling, walking, swimming for 30 minutes on 5 days a week. | MT (15 minutes) was provided in addition to a stretching and low-load exercise program (30 minutes). | AE were not reported. |
| Ganesh, 2015 | Posterior-anterior oscillatory techniques LVLA Maitland techniques with grades I-IV with a rate of 2-3 oscillations per second and a frequency of 3-4 mobilization of the joint lasting 30 seconds each and one minute rest time in between. In addition, patients received the same intervention as the control group.  *Maitland LVLA* | Supervised exercise program consisting of flexibility and strengthening exercises with stretching to cervical and scapular muscles, deep neck flexor strengthening, isometric exercises for extensors, side flexors (both sides) and rotators (both sides), anti-gravity strengthening to rhomboids, middle and lower trapezius and cervical ROM exercises with 1x10 repetitions with 6 seconds holding and 10 seconds rest in between. Further all participants received postural advice and to continue the exercises four weeks at home. | MT (of unclear duration) was provided in addition to the stretching and strengthening exercise and postural advice. | No serious AE were reported, only transient muscle and joint soreness for some participants of the mobilization group. |
| Gonzalez-Iglesias, 2009a | HVLA-techniques seated for the thoracic spine once per week for three weeks. If no popping was heard on the first attempt, the therapist repositioned the patient and performed a second manipulation, which was the maximum allowed per patient per session. In addition, patients received the same treatment as the control group.  *HVLA, concept not specified* | Both groups received electrotherapy and a thermal program with an infrared lamp (250 W) located 50cm from the patients’ neck for 15 minutes. After that TENS with a frequency of 100 Hz and 250ms stimulation was applied for 20 minutes with 2 electrodes bilaterally to C7 spinosus processus. | MT (maximum of two HVLA) was provided in addition to the infrared thermotherapy and TENS. | AE were not reported. |
| Gonzalez-Iglesias, 2009b | HVLA-techniques seated for the thoracic spine on three consecutive Mondays. If no popping was heard on the first attempt, the therapist repositioned the patient and performed a second manipulation, which was the maximum allowed per patient per session. In addition, patients received the same treatment as the control group.  *HVLA, concept not specified* | Both groups received electrotherapy and a thermal program with an infrared lamp (250 W) located 50cm from the patients’ neck for 15 minutes. After that TENS with a frequency of 100 Hz and 250ms stimulation was applied for 20 minutes with 2 electrodes bilaterally to C7 spinosus processus. | MT (maximum of two HVLA) was provided in addition to the infrared thermotherapy and TENS. | AE were not reported |
| Gonzalez-Rueda, 2021 | LVLA-techniques with upper cervical glide mobilization of the upper cervical joints and a force direction dorsally from the shoulder to the patient’s forehead with 5 minutes and cycles of 30 seconds for mobilization and 10 seconds rest with a slight pressure to perform a stretching mobilization. In addition, patients received the same physiotherapy program as the control group.  *Kaltenborn LVLA* | Superficial thermotherapy (20 minutes) with a heat lamp, stretching and auto-traction of the cervical spine and thoracic mobilization exercises for upper trapezius, levator scapulae, pectoralis major muscles. Stretching was performed 10x15 seconds with 5 seconds rest in between. Cervical spine auto-traction was performed by patients themselves under the occiput for 10x15 seconds and 5 seconds rest, thoracic spine auto-mobilization with a Kaltenborn wedge 2x15 seconds for each segment and 5 seconds’ rest. | MT (5 minutes in 6 of 15 sessions extra) was provided on top of… | No serious AE. |
| Grunnesjo, 2004 | Two general medicine practitioners and 9 physiotherapists treated this group. The basic strategy was the same stay active concept as the CG received. In addition, patients received manual therapy or muscle stretching with or without matching home exercises. LVLA-techniques by the two physicians and physiotherapists in 2/3 cases. However, in this group physicians also prescribed steroids and local anaesthetics’ several times.  *Kaltenborn LVLA* | Two orthopaedic surgeons and 8 physiotherapists treated this group. Patients were instructed to stay active and take part in physical activities and stay fit and physicians were instructed to avoid sick certificates or certify them as short as possible with advice to stretch if it feels good. | MT (of unclear duration) was provided in addition to the stay active concept and analgesics and in some cases patients in the IG also received steroid injections and local anaesthetics. | AE were not reported. |
| Gutierrez-Espinoza, 2023 | Patients in this group received passive manual scapular mobilization techniques with superior and inferior glides, rotations, and distractions to the scapula. 3x10 repetitions with one cycle taking 6 seconds, 30 second break in between. In addition, the same exercise program was performed as the CG had.  *LVLA, concept not specified* | Standardized exercise program with stretching and strengthening of the rotator cuff and scapular muscles. Stretching exercises targeted the upper trapezius, pectoralis minor, and posterior region of the shoulder with 3x30 seconds and 30 second rest in between. Following this, 3 strengthening exercises were performed with elastic resistance bands, external shoulder rotation, shoulder extension and shoulder protraction targeting the serratus anterior. 3x10 repetitions with 1 minute rest in between. | MT (of unclear duration) was provided in addition to an exercise and stretching program. | AE were not reported. |
| Haider, 2018 | Thoracic LVLA- and several HVLA-techniques without exact dosage or specification of frequency were provided to patients in the IG in addition to the treatment of the control group.  *Maitland LVLA & HVLA* | Hot or cold pack at the thoracic spine and exercise therapy for mobility with flexion and extension exercises, strengthening exercises with an elastic band, holding bar for shoulder rotation and flexion. Dosage and further details were not reported in the trial. | MT (of unclear duration) was provided in addition to an exercise, mobility and strengthening, and physical therapy program with hot/ cold pack. | AE were not reported. |
| Hallegraeff, 2009 | LVLA- and HVLA-techniques to the sacroiliac joint according to the physical examination of the therapist and no other technique was applied, in each treatment session only one manipulation was applied with an added time investment of approximately four minutes. This was in addition to the treatment of the control group.  *LVLA & HVLA, concept not specified* | Gradually increasing the level of physical activity by prescribing a low intensity, low endurance exercise program to train abdominal oblique and straight muscles for 2 minutes, stretch lumbar extensors for 2 minutes with approximately 5 minutes twice per day. Further patients received an information leaflet and the advice to stay active along with postural advice. | MT (4 minutes per session) was provided in addition to a home exercise program, postural advice, and advice to stay active. | AE were not reported. |
| Hancock, 2007 | LVLA- and HVLA-techniques were permitted while most patients received mostly LVLA techniques a small proportion also were treated with HVLA-techniques. Treatment was adjusted to the clinical presentation rather than applying the same treatment to all patients.  *Maitland LVLA & HVLA* | Advice from the general practitioner along with paracetamol as needed, maximum of 4g per day. LVLA and HVLA were controlled for with sham intervention with a detuned ultrasound machine to account for the additional time. | MT (30-40 minutes initially, and 20 minutes following sessions) was provided in addition to usual care but was controlled for with a sham intervention (detuned ultrasound machine). So, the additional time was accounted for. | There was 11 mild AE in the intervention- and 11 AE in the CG while non was related to the manual intervention but rather the diclofenac and placebo medication comparison. No serious AE were reported. |
| Hoving, 2006 | Muscular, articular LVLA-techniques and coordination or stabilization techniques to treat segmental movement dysfunctions. Therapists could instruct patients to perform home exercises. No further information was provided in terms of dosage, frequency, duration etc.  *LVLA, concept not specified* | Individualized PT with exercise, active and passive movement strategies and stretching and functional exercises were performed. Manual traction or massage could be applied but advanced mobilization techniques were not allowed. Technically this was usual care, and the IG received manual therapy instead, not in addition to usual care. | MT (of unclear duration) was compared with conventional PT (of unclear duration) but was performed once weekly while PT took place twice per week, because the study design was pragmatic. | AE were not reported. |
| Javadov, 2021 | SIJ LVLA- and HVLA-techniques in three sessions were administered. The dosage, frequency was not further specified. In addition, patients received the same home exercise program as the CG did.  *LVLA & HVLA, concept not specified* | Patients were instructed to perform a home exercise program with 2x10 repetitions per day with 20 seconds break in between. Exercises included SIJ self-mobilization, piriformis stretch, gluteus medius and minimums stretch, gluteus maximus stretch, stretching gluteal and piriformis muscles together, hip muscle isometric strengthening exercises. | MT (of unclear duration) was provided in addition to a home exercise program. | AE were not reported. |
| Joshi, 2014 | Maitland compression techniques with medial glide of the patella was administered with 3x15 compressions and vastus medialis obliquus strengthening exercises and 20 minutes of short-wave diathermy with an anterior posterior alignment of electrodes. The vastus medialis obliquus program was the same as the control IG received.  *Maitland LVLA* | Patients received short wave diathermy for 20 minutes and a vastus medialis (VMO) strengthening program with exercises in sitting (squeezing rolled towel between knees), contracting gluteal muscles at the same time and progression to standing and stepping up- and down while emphasizing the VMO muscle. No Maitland compression techniques were administered. The frequency, dosage or training parameters were not further specified. | MT (of unclear duration) was provided in addition to short-wave diathermy for the knee and a strengthening program. | AE were not reported. |
| Jull, 2002 | LVLA- and HVLA Maitland techniques were administered to the cervical spine where therapists examine the patient and adjust the treatment according to their examination. Dosage is not further specified. Patients in the IG received the same exercise program as the CG did.  *Maitland LVLA & HVLA* | Patients performed a low-load endurance exercise program to train muscle control of the cervicoscapular region with specific exercises to address impairment of neck flexor synergy. Craniocervical flexion exercises with pressure sensor feedback, muscles of the scapular with serratus anterior and lower trapezius were trained through scapular adduction and retraction. These two formal exercises were required to be done twice daily to increase endurance capacity. In addition, patients were instructed to perform isometric exercises with a low level of rotatory resistance to train co-contraction of neck flexors and extensors. Further patients received postural correction exercises during the day. Dosage and training parameters were not specified further. | MT was provided within the treatment time of approximately 30 minutes. No additional time was spent with patients in the intervention group. | No serious AE. |
| Jüni, 2009 | Within 24 hours of randomisation patients underwent HVLA- and LVLA-techniques, and muscle energy techniques with a maximum of five appointments, according to each individual presentation. In addition, patients received usual care as well like the control group.  *Maitland LVLA & HVLA* | Advice to return to normal activities and avoid bed rest and paracetamol, diclofenac or dihydrocodeine were prescribed from the treating physician. Other treatments, like physiotherapy, or other drugs were not allowed. | MT (of unclear duration) was provided in addition to usual care consisting of paracetamol or other analgesic drugs from the general practitioner and no other interventions allowed. | 2 AE in the intervention (acute pancreatitis, acute motor, and sensory loss due to a herniated disc at L5 before treatment) and 2 AE in the CG (cholelithiasis and femoroacetabular impingement), however, none were study related. |
| Just, 2009 | 10 minutes of LVLA-techniques for the shoulder, the specific techniques were chosen by the therapist according to the individual presentation, mostly comprising Kaltenborn mobilization techniques like gliding, traction and passive mobilization. No further specification of dosage, techniques or else was made.  *Kaltenborn LVLA* | Individual strengthening of the rotator cuff, stabilization exercises of scapular muscles and massage and self-mobilization of the shoulder joint capsule like Klein-Vogelbach, Spiraldynamik (German techniques). No further specification of dosage, exercises or else was made. | MT (of unclear duration) was provided within the 30 minutes treatment time and the exercises and massage techniques. | No serious study-related AE. 4 patients dropped out due to radicular pain after the initial examination before treatment administration (in the control group). |
| Kachingwe, 2008 | LVLA-techniques with anterior, posterior, and inferior glenohumeral joint glides, long-axis distraction grades I-IV depending on the reactivity (Kaltenborn). Each mobilization was applied for 30 seconds at a rate of one mobilization every 1-2 seconds followed by a 30 second rest, totalling 3x30 seconds mobilization per technique.  *Kaltenborn LVLA* | Supervised exercises with posterior capsule stretching postural correction exercises, and an exercise program focusing on rotator cuff strengthening and scapular stabilization with external rotation and an elastic band and scapular retraction. Every treatment ended with 10-15 minutes cold pack to decrease potential inflammation, and participants were instructed to perform a home exercise program once daily. No further specification was mode for dosage, frequency etc. | MT (of unclear duration) was provided in addition to a supervised exercise program, a home exercise program and cold pack. | AE were not reported. |
| Khan, 2016 | Intermittent manual cervical traction with a towel was given for 20 minutes with 10 seconds traction period and 5 second rest. In addition, patients received the same treatments as the control group.  *Kaltenborn LVLA* | Combination of active range of motion exercises, TENS, and superficial thermotherapy. Patients were trained to execute all the exercises 2x25 repetitions daily. TENS was provided on constant mode for 20 minutes. | MT (20 minutes) was provided in addition to an exercise program, TENS, and superficial thermo-therapy. | AE were not reported. |
| Kromer, 2013 | Individually adapted exercises and individualized manual PT. LVLA- and HVLA Kaltenborn techniques according to the individual presentation with glide techniques and anterior-posterior techniques, neural glides according to Butler. Initially gliding and stretching was done for 20-30 seconds and subsequently progressed on an individual basis. Patients received the same exercise program as the control group.  *Kaltenborn LVLA & HVLA* | 2/day for the first week, then once daily with dynamic exercises 2x10 repetitions with elastic bands. Shoulder and neck stretch for 2x10 seconds and progressed with sets going from 2 to 3 and repetitions from 10 to 20. Further the yellow elastic band was replaced with a green one, higher resistance. Patients received six sessions of supervised exercises and were instructed to perform home exercises (2 of 8) daily. | MT (10-15 minutes) was provided in addition to a supervised exercise program and a home exercise program. | AE were not reported. |
| Kulkarni, 2016 | MWM mulligan techniques according to the individual presentation with medial or lateral glides, rotation MWM with 3 repetitions on day one and progressive increase of load with 6 on day 2, 9 on day 3 etc. Patients received the same treatment regimen as the CG did in addition.  *Mulligan MWM* | TENS with 100Hz frequency for 15-20 minutes was applied as well as an exercise program consisting of isometric exercises and stretching for the entire lower limb. 3x10 repetitions with 6 seconds holing phase and 3 seconds break in between. Hip adduction with a towel in between the thighs, hamstring contraction while lying supine and quadriceps contraction with a towel under the knee were the 3 exercises. Further calf, quadriceps, hamstring stretches were performed 3/day for 2 days. | MT (of unclear duration) was provided in addition to an exercise and stretching program. | AE were not reported. |
| Lalnunpuii, 2016 | Maitland LVLA-techniques grade II-III glides performed according to the individual presentation with tibiofemoral anterior-posterior glide, tibio-femoral posterioranterior glide, patello-femoral-caudal-cephlaoid glide with 2-3 oscillations per second for 1-2 minutes. In addition, individuals performed the same exercise program as the control group.  *Maitland LVLA* | Isometric quadriceps and hamstring strengthening exercises with 4x10 repetitions of 15 second holding phases. Flexibility exercises as quadriceps and hamstring stretches 3x30 seconds, knee range of motion exercises 2x30 seconds under supervision of the therapist. Warm up and cool down exercises for 5-10 minutes were given before starting and after the completion of exercises in all groups. | MT (of unclear duration) was provided in addition to an exercise and stretching program. | AE were not reported. |
| Lau, 2011 | HVLA-technique to the thoracic spine was applied, if no popping sound was heard the patient was repositioned and a second attempt was performed on each subject per session.  *HVLA, concept not specified* | Infrared radiation therapy for 15 minutes over the painful cervical area. A standardized set of informational material and general advice on neck care was given as well as neck exercises prescribed involving active neck mobilization (10 repetitions), isometric contractions (flexion, extension, side flexion, rotation for 10x 5seconds), stretching of upper trapezius and scalene muscles (10x5-8s seconds) and postural correction exercise. | MT (of unclear duration) was provided in addition to an infrared radiation therapy and an educational booklet with a prescription of the same home exercise program. | No serious AE. |
| Lee, 2016 | HVLA-techniques for 10 minutes according to the examination of the therapist. Deep cervical flexor training for 15 minutes, self-stretching of levator scapulae and upper trapezius muscles as cool down exercises for 10 minutes. So, both groups received 35 minutes of treatment time.  *HVLA, concept not specified* | Deep cervical flexor training for 25 minutes, self-stretching of levator scapulae and upper trapezius muscles as cool down exercises for 10 minutes. The exercise intensity was determined by the patient’s status and increased progressively and a biofeedback unit to provide visual feedback of the pressure level and patients were instructed to target levels between 22-30mmhg. Isometric contractions were performed 10x10 seconds with 5 seconds rest in between. | MT was provided within the treatment time (35 minutes) and alongside an exercise and stretching program. | No serious AE. |
| Lytras, 2023 | Light massage and stretching prior to the HVLA-technique. Stretching was applied to the upper part of the trapezius, levator scapulae, sternocleidomastoid muscle and the manual techniques comprised LVLA, HVLA for 15 minutes. Patients also performed the same 45-minute exercise program as the control group.  *HVLA, concept not specified* | Endurance and resistance training exercises for the neck and scapula muscles with retraining of the longus colli, endurance training of the deep cervical flexors, neck muscle ROM exercises in all directions, resistance exercises for the neck and the upper limb (12-15 repetitions) with resistance bands, isometric exercise for neck flexion, extension, side bending, and rotation wit 20-70% of maximum voluntary contraction capacity. Ion top patients performed stretching exercises for the neck and upper limb muscles. | MT was provided on top (15 minutes) of the exercise and stretching program (45 minutes). | AE were not reported. |
| Maiers, 2014 | Participants received up to 20 visits of HVLA-techniques based upon the examination findings and individualized by the therapist. No further specifications were made. This was complemented with optional adjunct therapies like soft tissue massage, hot and cold packs were applied to the cervical and upper thoracic area. The same program for home exercise was performed by this group than the control group.  *HVLA, concept not specified* | Four 45–60-minute sessions with participants receiving basic information regarding pain management, postural instructions, and practical demonstrations of body mechanics for lifting, pushing, pulling, and rising from lying down. They were instructed to stay active, exercise daily to improve flexibility, balance, coordination, and strength. Exercises included head retraction, cervical flexion, and extension isometric or resistance tubing, full spine flexion/ extension cycles. Exercises were progressed as soon as 20 repetitions of an exercise could be done with proper form. No further specification was made concerning dosage, frequency, etc. | MT (additional maximum of 20 visits per patient) with optional adjunct therapies like soft tissue massage, hot and cold packs were applied to the cervical and upper thoracic area. | One participant fell and fractured his radius while performing study-related exercises during a supervised visit. Nonserious AE like more pain and muscle soreness were frequent. 58% in home exercise and 56% in SMT and home exercise group. |
| Menek, 2019 | MWM techniques for flexion, abduction, external and internal rotation according to the individual presentation. 3x10 repetitions and a rest interval of 30 seconds in between each sequence was performed, totalling 20 minutes. Further this group received the same treatment regimen as the control group.  *Mulligan MWM* | Exercise program once a day for 5 days a week. Codman exercises, finger stairs, shoulder handwheel, shoulder capsule stretching exercises were performed. In addition, flexion, abduction, extension, external and internal rotation stretching for the shoulder (5x20 seconds). Strengthening exercises were progressed with elastic band (3x10 repetitions) for shoulder flexion, abduction, extension, external and internal rotation with one minute rest in between each exercise. Both groups also received 1.5 MHz ultrasound for 6 minutes and 100 Hz TENS for 20 minutes. | MT was provided on top (20 minutes) of exercise and stretching program, ultrasound, and TENS. | AE were not reported. |
| Michener, 2024 | HVLA- and LVLA-techniques were administered at the thoracic spine, posterior shoulder and glenohumeral joint. Clinicians performed these techniques for a total of 10-15 minutes and were required to use at least 1 technique for each of the three body regions and could select low-grade or high-grade techniques according to the irritability.  Techniques used comprised thoracic posterior-anterior glides in prone, seated glides, HVLA-thrusts in prone and supine for a maximum of 2 attempts. Thoracic distraction thrust while seating, posterior glide glenohumeral mobilization in prone, Mulligan MWM posterior glide with elevation, posterior shoulder stretch, passive stretching into internal rotation, inferior glenohumeral glides in supine, posterior anterior glides on the clavicle, inferior glide of clavicle on the acromion.  Techniques and intensity were chosen by the treating therapist.  LVLA & HVLA, concept not specified | Three phase exercise programs with stretching. Body weight or exercise bands were used to target scapular stabilizers, rotator cuff and flexibility exercises and exercises to promote an erect posture through chin tucks and scapular retraction were performed. 2-3x10 repetitions with an elastic rubber band were performed.  Phase I: resistance training for the rotator cuff muscles.  Phase II: continued with resistance exercises of the shoulder external and internal rotators with a focus on the rotator cuff muscles at 45-90° degrees of elevation in shoulder scaption, shoulder elevation exercises, and the addition of progressive strengthening of serratus and middle and lower trapezius.  Phase III: all the Phase II exercises with the addition of higher-level resistance exercises including lawn mower pull, protraction, planks, and use of the Bodyblade at multiple angles of shoulder elevation. Individuals progressed to more resistance (colour of elastic band) when they were able to perform 2-3 sets of 10 repetitions with minimal symptoms or fatigue. Individuals went from Phase I to Phase II when they were able to perform full sets and repetitions of the exercises with a red resistive band and from Phase II to Phase III when they could perform all exercises in Phase II for 1 week with minimal symptoms. | MT was provided on top (10-15 minutes each session) of an exercise program. | No serious AE. |
| Mintken, 2016 | High-dose cervicothoracic MT during the first two visits and cervicothoracic range of motion exercises. During visits 3 to 8 both groups had the same comprehensive stretching and strengthening program. The techniques during the first two visits comprised 5 thoracic HVLA-techniques targeting upper, middle, and lower thoracic spine and one LVLA-technique directed at the lower cervical spine. Individuals received HVLA-techniques up to two times unless a cavitation was noted for up to 10 HVLA-technique per treatment session.  *MWM & LVLA, concept not specified* | Two sessions of cervicothoracic ROM exercises. First one was general cervical exercise called 3-finger ROM exercise, second was a general thoracic-mobility exercise in supine over a towel. Individuals performed these 3-4x10 repetitions per day. During visits 3 through 8 patients were instructed to perform a stretching and 3-phase progressive strengthening program comprising exercises for scapular stabilizers, rotator cuff, flexibility exercises and exercises to promote an erect posture through chin tucks and scapular retraction. Individuals did these 2-3x10 per day with elastic bands. They progressed from phase I to phase II when they were able to perform full sets and repetitions of the exercises with a resistive band.  Phase I: resistance training for the rotator cuff muscles.  Phase II: continued with resistance exercises of the shoulder external and internal rotators with a focus on the rotator cuff muscles at 45-90° degrees of elevation in shoulder scaption, shoulder elevation exercises, and the addition of progressive strengthening of serratus and middle and lower trapezius.  Phase III: all the Phase II exercises with the addition of higher-level resistance exercises including lawn mower pull, protraction, planks, and use of the Bodyblade at multiple angles of shoulder elevation. | MT was provided in addition to cervicothoracic ROM exercises and a progressive stretching and strengthening program. | No serious AE. |
| Mostamad, 2023 | Patients also received MWM mulligan techniques 3x10 repetitions with rotational pressure on the tibia while moving the knee into flexion. Patients also received the same physical therapy program as the intervention group.  *Mulligan MWM* | Patients received electrotherapy with 60-100 Hz TENS with 60ms pulse duration, each session lasting 20 minutes, continuous US therapy (1 MHz with 1.5W/cm² for 10 minutes) using Ultrasound 215A, 20 minutes of hot pack and quadriceps exercises for 10 minutes without further specification of the exercises in question. | MT (of unclear duration) was provided in addition to an exercise program, TENS, ultrasound, and hot pack. | AE were not reported. |
| Murphy, 2010 | HVLA- and LVLA-techniques after physical examination and according to the clinician’s judgement one or two treatments per week with treatment direct at the cervical and upper thoracic spine. Myofascial trigger points were also treated as needed in the cervical muscles and the shoulder, wrist and elbow were treated as needed. Patients received the same exercise program as the control group.  *LVLA & HVLA, concept not specified* | Both groups were taught how to achieve a neutral spine, pelvis and correct diaphragmatic breathing and postural alignment, correct postures in everyday living and about possible causes of neck pain Subjects were prescribed a strength and endurance program of 1-2x6-8 repetitions for isometric exercises held for a duration of 3-5 seconds, 1-2x12-15 repetitions for dynamic exercises (3-0-3 eccentric/concentric) with 4/5 ratings of perceived exertion rate. They were also encouraged to undertake 20 minutes of aerobic activity on 2-3 days per week at an intensity of 4/5 perceived exhaustion. After one-month patients were progressed to 3x12 repetitions and an intensity of 5-6 rate of perceived exhaustion and continued aerobic activity for 25 minutes on 2-3 days per week. Exercises consisted of wall angels, range of motion exercises for the shoulder with flexion, abduction, scapular retraction, cervical extension, cervical retraction, quadruped, plank, wall squats, dead bug, seated hip flexion, triceps extension, flexion and abduction with elastic rubber band, scapular rowing, wall push ups. Advanced dead bugs and roll outs. | MT (of unclear duration) was provided in addition to a comprehensive exercise program. | AE were not reported. |
| Nagata, 2019 | Patients received Jog-Manipulation which was developed by the authors department as a combination of several types of manipulations: pivot made of gauze was set on the last molar, then closing type with fulcrums on both sides, side-to-side-type, opening type, closing type with fulcrums on the impaired side were executed continuously. If an insufficient opening of <40mm of the mouth could be obtained, the same process was repeated three times at the first visit and each subsequent visit of the patients until the restoration of mouth-opening limitation <40mm. Patients also the same treatment as the control group.  *HVLA, concept not specified* | Standard therapy was provided to both groups with cognitive behavioural therapy, education, and self-exercise. Self-exercise consisted of one exercise were patients pulled down on their bilateral lower last molar with their secondary fingers, while opening the jaw to the greatest possible extent with 3x5 repetitions. The second exercise comprised simplified myofunctional therapy with maximum mouth opening, clenching, protrusion of the lip, maximum mouth opening and maximum tongue protrusion without use of the patient’s finger. Patients were instructed to execute every hour throughout the day. Patients were guided to perform two types of self-exercise, if sufficient recovery of the mouth opening had been achieved, patients were prescribed a reduction of exercise strength. | MT (of unclear duration) was provided in addition to cognitive behavioural therapy, education, and self-exercises. | AE were not reported. |
| Nam, 2013 | Patients in the experimental group received the same treatment as the CG with the addition of MWM Mulligan techniques. Patients performed internal rotation on the tibia while the patient flexed their knee, and the patient repeated this movement 10 times without pain and knee joint gliding without pain 3x10 with 30-second breaks in between.  *Mulligan MWM* | Patients did not limit their daily movements or occupational activities and were instructed to not seek treatment other than the study-related treatments. Patients received 10 minutes of hot pack, interferential current therapy for 20 minutes and ultrasound therapy for 5 minutes. Then patients performed trunk stabilization exercises. Patients lay prone, lifted both legs through cross extension without knee flexion and repeated the motion for 10 seconds without pain, and lowered the legs ten times. 3 sets with 30 seconds breaks between each session. Therapists fixed the patients lumbar region to prevent corresponding activities of the upper extremities. | MT (of unclear duration) was provided in addition to hot packs, electrotherapy, ultrasound, and stabilization exercises. | AE were not reported. |
| Narang, 2014 | Patients received Kaltenborn LVLA-techniques with traction, dorsal and ventral gliding, patella mobilization laterally and medially.  *Kaltenborn LVLA* | Patients received hot packs once in the treatment facility and a second time at home for 15-30 minutes. They also performed isometric quadriceps exercises while lying supine with a towel under the knee and isometrically contract the quadriceps for 10 seconds and isotonically while sitting with a weight cuff tied around the ankle to increase the resistance though the 90° degrees of flexion to full extension and resistance. | MT (of unclear duration) was provided in addition to isometric and isotonic low-load exercises. | AE were not reported. |
| Naranjo-Cinto, 2022 | Patients in the IG received the same exercise program as the CG and real MT. Patients received glenohumeral LVLA-techniques in supine with 30° degrees of shoulder abduction and 90° elbow flexion with traction to the glenohumeral head and inducing a flexion-extension movement to the elbow with their forearm at the same time. When traction was applied the elbow as moved toward flexion and when traction was released, the elbow was moved toward extension (neural sliding mobilization technique) with 3x15 repetitions at 2 >Hz frequency. In addition, patients received a rib-cage technique with ipsilateral LVLA-technique near the second rib and costotransversal joint with posterior-anterior LVLA-techniques over 3 minutes at 2 Hz frequency.  *LVLA, concept not specified* | Both groups received a therapeutic exercise program consisting of isometric exercises with a progressive load. They were self-resisted by the patient in a sitting position and performed shoulder flexion, abduction, internal and external rotation with 3x20 seconds of contraction and 10 seconds rest in between one set. The patients were instructed to perform these every day to increase the load based on their tolerance.  Patients in the CG received sham MT that was conducted in the same position as the real MT. The therapist placed their hands in the same way as the real MT without inducing any type of movement but maintained the contact with their hands on the skin of the patient for 3 minutes. Treatment was provided without pain. | MT (of unclear duration) was controlled for with a specific sham MT intervention to account for physical contact, context, and attention. Patients in both groups performed isometric low-load exercises daily. | No serious AE. |
| Nejati, 2019 | Patients performed the same exercise program as the control group. In the IG patients received MT with 2 manoeuvres of innominate rotation with posterior mobilization and SIJ manipulation with LVLA- and HVLA-techniques in supine.  *LVLA & HVLA, concept not specified* | Patients were instructed on how to perform the exercises at home daily and were supervised once at home for 12 weeks. Patients were instructed to perform posterior innominate self-mobilization in supine, sacroiliac joint stretches, spinal stabilization exercises in 4 phases:  Phase I: supine abdominal draw-in, abdominal draw-in with one knee drawn to chest, abdominal draw-in with the heels sliding backward one after the other, abdominal draw-in with both knees drawn to the chest, supine twist, prone bridging on elbows, side bridging on elbows, prone cobra, quadruped opposite arm-leg lift.  Phase II: abdominal draw-in with feet on the medicine ball plus abdominal draw-in with feed on the ball and added movement, prone bridging on elbows with single-leg hip extension, quadruped opposite arm-leg lifts, with cuff or dumbbell weights.  Phase III: prone bridging, with the feet on the ball, side bridging with single-leg hip abduction, quadruped opposite arm-leg lifts on half foam rollers, twisting while seated on medicine ball.  Phase IV: soccer ball-size medicine ball to the patient who resisted the movement and performed the same exercises as in phase III. Each exercise was done 10 times per day. | MT (of unclear duration) was provided in addition to a supervised and home exercise program. | AE were not reported. |
| Nigam, 2020 | Patients in the IG received the same exercise program and moist heat application as the control group. They also received MWM Mulligan techniques to the affected knee prior to the exercise program. Therapists applied pain free manual sustained glide force to the proximal tibia in lateral, medial, rotational, anterior, or posterior direction. While this was maintained, subjects were instructed to move the knee in the symptomatic direction towards flexion or extension as far as possible without pain. The direction of glide, which had the best effect, was chosen for the treatment. The technique was progressed to weight bearing once full range was achieved pain free while lying supine. 3x6-10 repetitions were delivered in each session. Patients also learnt self-applied MWM in the first session and were instructed to perform these at home.  *MWM Mulligan* | Subjects received moist heat for 15 minutes from a hydrocollator pack wrapped in soft towel around the knee and an exercise program. Exercises included pelvic bridging against body weight in crook lying, lifting the pelvis for 5 seconds. Knee flexion was performed in prone lying while knee extension was performed in sitting. Resistance was provided with a weighted ankle cuff commencing at 1kg and progressing to 2 kg. Mini squat exercises were undertaken in standing and involved closed chain hip- and knee flexion. Single leg heel raise exercise was performed against body weight with 3x15 repetitions and progressed 5x20 repetitions as tolerated. Patients were instructed to perform the supervised exercises daily at home to and were advised to undertake brisk walking daily for 20 minutes. | MT (of unclear duration) was provided in addition to a supervised and home exercise program and moist heat application. | No serious AE. |
| Ojo Ojoawo, 2016 | Patients in the IG performed the same exercises as the control group, received the same massage and an ice pack. In addition, they received transvers oscillatory pressure techniques lying prone to the cervical spine rhythmically for 3x20 seconds with a rest period of 2 minutes for a session per day.  *Maitland LVLA* | Both groups underwent the same exercise program with cervical spine retraction, rotation in each direction, extension, contralateral side-bending stretching exercises, passive stretching and isometric exercises of posterior neck muscles and isometric exercises to the posterior neck muscles with 10x10 seconds against the therapist’s hand. In addition, patients received ice chips in a towel to the cervical region for 7 minutes and kneading massage with methyl salicylate ointment for 3 minutes. No further dosage for the exercise regimen was specified. | MT (of unclear duration) was provided in addition to an exercise program, massage, and ice therapy. | AE were not reported. |
| Ojo Ojoawo, 2018 | Patients in the IG performed the same exercises as the control group, received the same massage and an ice pack. In addition, they received transvers oscillatory pressure techniques lying prone to the cervical spine rhythmically for 3x20 seconds with a rest period of 2 minutes for a session per day.  *Maitland LVLA* | Both groups underwent the same exercise program with cervical spine retraction, rotation in each direction, extension, contralateral side-bending stretching exercises, passive stretching and isometric exercises of posterior neck muscles and isometric exercises to the posterior neck muscles with 10x10 seconds against the therapist’s hand. In addition, patients received ice chips in a towel to the cervical region for 7 minutes and kneading massage with methyl salicylate ointment for 3 minutes. No further dosage for the exercise regimen was specified. | MT (of unclear duration) was provided in addition to an exercise program, massage, and ice therapy. | AE were not reported. |
| Park, 2020 | Patients in the IG performed the same exercises as the CG and received LVLA-techniques, more precise, oscillations with central posterior-anterior mobilization in prone 2x30 seconds with 1 minute rest in between. The exercise program also took 15 minutes with the addition of the MT program.  *Maitland LVLA* | A single physiotherapist with 3 sessions/week for 4 weeks each session taking 15 minutes supervised exercise program. Mobilization and exercise, interventions were performed in groups and all patients received the same number of interventions. Patients in the CG performed foam roll stretches, marching on a roller 2x10 repetitions, thoracic extension at the wall using bodyweight 2x10 repetitions and standing neck/ chest stretches as cool down for 15 minutes. | MT (of unclear duration) was provided in addition to an exercise program. | AE were not reported. |
| Poulsen, 2013 | Patients in the IG received the same educational program as the control group. In addition, they received trigger point release therapy to the posterior and lateral hip muscles with digital pressure being applied to trigger points until patients indicated numbness due to the pressure (1-3 minutes). Further patients received muscle energy technique with 10-20 second contraction of the antagonist muscle using 30% of full contraction potential and the therapist resisting that movement to achieve isometric contraction. This is immediately followed by agonist contraction in the direction of the resistance. This was done 3x10 in the directions of the affected range of movement. Lastly, patients received hip joint manipulation (HVLA-technique) with combined movements of flexion, internal rotation, flexion with external rotation or flexion with translatory abduction and manipulations could be assisted by a drop mechanism of the treatment table. The patient’s leg was placed 10-15° in abduction and 20-30° flexion, for the second technique the patient’s leg was placed in a loose packed position of 25-35° of abduction, 20-30° of flexion and 30-40° of external rotation. For the third technique, the leg was placed with 20-25° of abduction and 0-10° of flexion with the knee in slight flexion and the therapist’s hands placed around the distal ankle or distal femur and each manipulation could be applied 1-3 times.  *HVLA, concept not specified.* | Patients were instructed to not initiate or alter use of pain medication or glucosamine products during the intervention period. Patients in the CG received patient education with one personal 1-on-1 interview (45 minutes) on the pain experience, influence of activities of daily living and self-motivation and advice on exercise. Following this, patients attended three 1.5-hour group sessions where hip anatomy was taught, diagnosis of the disease was explained, muscle action and hip anatomy were explained and the importance of an active lifestyle emphasized. Lastly patients were educated on pain sensitization and self-management and alternative treatment options including medication and surgery. In a 30 minute, follow up interview patients could ask unanswered questions and were instructed to perform home stretching exercises. | MT (of unclear duration) was provided in addition to individual and group educational sessions. | No serious AE, however, 7 patients in the IG reported discomfort, muscle soreness or mild pain appearing up to 24h after MT, lasting no more than 24h and not affecting daily life. One patient reported moderate pain appearing after 4 weeks of therapy, lasting for 2 weeks, and having some effect on daily living. |
| Rasmussen, 2008 | Patients performed the same two exercises as the CG did. They also received a HVLA-technique at the perceived spinal level of reduced movement.  *HVLA, concept not specified* | Patients were instructed to perform the two exercises with a gradual increase of the extension with 4-6 sets of 3-5 repetitions. Patients were instructed to perform these exercises as often as possible during the day and at least half an hour. | MT (of unclear duration) was provided in addition to two extension exercises. | 4 Patients in the IG reported worsening of back pain after 4 weeks and 3 patients in the control group. Similar numbers were reported after 3- and 12 months. |
| Reynolds, 2020 | Participants received the same treatments as the control group. The MT techniques were HVLA-technique in supine to the upper cervical spine at C0-C1 with a distraction technique, a rotational upslope technique at C2-C3 on each side. If cavitation occurred on the first trial, the therapist moved to the next location, if not a second attempt was made with a maximum of 2 attempts on each level on each side yielding 4-8 HVLA-technique at each visit.  *LVLA & HVLA, concept not specified* | Patients received 2 minutes of suboccipital release, education, and an instruction for a home exercise program. Education included a discussion of the role of PT, suggestions how to manage pain and on habits like gum chewing, clenching, biting and stress management and sleep hygiene. Patients were instructed to perform 6 standardized exercises (The Rocabado 6x6) and two additional exercises with lateral jaw movement training with a hyperboloid material placed between the central incisors and the goal was coordinative movement in the frontal plane. The second exercise was a 3-finger rotation for the cervical spine ROM. The Rocabado exercises consist of resting position, controlled opening, axial extension of the neck with overpressure and posture correction/ scapular retraction. The control intervention for the MT was a sham treatment. Clinicians placed the participant in the manipulation position stopping short of tissue tension and maintained the position for 15 seconds, repositioned to neutral or resting position with no thrust. Participants received this technique at each of the 4 locations. | MT (of unclear duration) was controlled for with a sham intervention with no thrust controlling for time, context, and physical contact in addition to a home exercise program, education, and 2 minutes of suboccipital release techniques for both groups. | No serious AE, 36% of the IG and 48% of the sham group reported side effects. The most severe side effect was aggravated neck pain lasting 7 days after the 1-week visit, other side effects were mild and moderate. |
| Rezaie, 2021 | Patients in the interventional group received the same routine treatment as the control group. They also received MT to the TMJ and cervical spine. Therapists applied medial and anterior-posterior mobilization (LVLA) to the TMJ, patients were asked to open and close the mouth 10 times, the therapist’s hand was placed over the thumb digit of another hand and the mandibular condyle was mobilized medially. Another mobilization direction was anterior-posterior and applied through the auditory canal with 1 oscillation per 2 seconds. Mobilization was 3x2 minutes and 30 seconds of rest in between, totalling 7 minutes. Patients also received MT to the cervical spine and soft tissue release techniques. MT to the cervical spine was also 1 oscillation every 2 seconds, 3x2 minutes with 30 seconds rest in between totalling 7 minutes to the upper cervical spine with posteriorly directed force on the frontal region of the patient while lying supine.  *LVLA, concept not specified* | Patients were asked not to take NSAIDs or muscle relaxants. All patients received routine treatment consisting of TENS for 15 minutes, ultrasound (1 MHz, 0.8-1.5W/cm² continuous output) for 5 minutes and a gentle massage for 25 minutes of the masseter in circular direction. | MT (15-20 minutes) was provided in addition to routine care which consisted of ultrasound, electrotherapy, and a gentle massage. | AE were not reported. |
| Rodriguez-Sanz, 2020 | Patients performed the same exercise program as the control group. They also received HVLA and LVLA-techniques to the upper cervical spine depending on each patient’s clinical findings. Therapists used C0-C1 and C2-3 techniques and if necessary C1-C2 techniques. Techniques were traction manipulation and upper cervical translatoric dorsal, ventral-cranial glides. Patients in this group rested 30 seconds between repetitions instead of 40 seconds to apply the MT techniques and maintain the same session length as the control group. The authors made no further dosage or intensity specification.  *Kaltenborn LVLA & HVLA* | 1/week for four weeks with each exercise session lasting 20 minutes with 2x10 repetitions, holding each exercise for 10 seconds with a 40 second rest between each repetition and two minutes between blocks. Initially, patients started with performing cervical stabilization exercises and performed contraction of deep neck flexor muscle activity. This was followed by exercises involving other muscles (flexion, extension, rotations, inclinations) and external resistance was used to increase the intensity of the exercises and was advanced unilaterally towards the most symptomatic side. All exercises were performed with prior contraction of the deep flexors; patients were encouraged to perform home exercises every day 2-5 times per day after the first session. | MT (of unclear duration) was included in the treatment time, so no additional time was spent with patients in the intervention group, both groups performed an exercise program and home exercise program. | No serious AE, however, 3 participants of the CG reported mild and transient aggravation of neck pain in the 6 months follow-up. |
| Rodriguez-Sanz, 2022 | Patients in this group performed the same exercise as the CG with the only difference being that the rest between each exercise repetition was 30 seconds to have 3 minutes to apply the MT techniques. The MT were HVLA and LVLA to the upper cervical spine with traction manipulation on C0-C1, C1-2, C2-C3, interapophysiary traction manipulation C2-3 and upper cervical translatoric dorsal glide C0-C1, C1-2, ventral-cranial glide C2-3. The authors made no further dosage or intensity specification.  *Kaltenborn LVLA & HVLA* | Patients performed cervical stabilization exercises with Biofeedback for the deep neck flexor muscle activity. Exercises were undertaken in supine with the cervical spine in neutral position and without pain. 2x10 repetitions with 10 seconds holding phase and 40 second rest between each repetition and 2 minutes between blocks. | MT (of unclear duration) was included in the treatment time, so no additional time was spent with patients in the intervention group. | AE were not reported. |
| Sai, 2015 | Patients in the IG performed the same exercises and home-exercise program as the control group. They also received MWM Mulligan techniques to the involved shoulder with poster-lateral force on the anterior aspect of the humeral head while being asked to perform the affected movement to the end of pain-free range with overpressure under sustained gliding force (3x10 repetitions with 30 seconds rest between each set).  *Mulligan MWM* | Codman’s exercises, stretching exercises and scapular setting exercises were provided to patients in both groups. All patients were instructed to perform these as a home exercise program twice daily as well. No further specification was made concerning repetitions, dosage, and frequency. | MT (of unclear duration) was provided in addition to an exercise program and home exercise program. | AE were not reported. |
| Satpute, 2015 | The IG received the same exercise and home exercise program as the control group. In addition, they received MWM mulligan techniques to the shoulder with a caudal glide along the line of the humerus while stabilizing the scapula. The patient was encouraged to move his or her arm actively behind his or her back with assistance from the therapist’s abdomen against the patient’s humerus. At the end, an overpressure to the movement was applied. 3x10 repetitions with 60 seconds rest intervals between each set. Exercises should always be pain-free.  *Mulligan MWM* | Patients received moist hot packs for the shoulder region 10 minutes before exercise and then performed a structured exercise protocol under supervision. Resistance bands were used for strengthening isometrically and hold a position for 10 seconds. Shoulder flexion in supine, scapular retraction in prone, scapular retraction in standing, shoulder internal and external rotation with the arm by the side, scapular protraction in standing 10x10 seconds followed by a 5-second rest. In addition, they performed stretching exercises targeted to the shoulder posterior capsule; one exercise was lying on the affected side, 90° elbow flexion, 90° shoulder flexion and internal rotation which was held for 5x30 seconds with rest intervals of 10 seconds. The second stretch was with assistance from the patients opposite hand 5x30 seconds with a rest interval of 10 seconds. A home exercise program was given to all participants comprising all exercises above without resistance band with the instruction to perform the program on treatment day once and on no treatment days twice. | MT (of unclear duration) was provided in addition to an exercise and home exercise program. | AE were not reported. |
| Satpute, 2019 | Patients received the same treatment as the control group, in addition they received spinal mobilization with leg movement for 5 minutes and an encouragement to continue with a home exercise program.  Patients were asked to move their affected leg to the limit of pain-free range of the single leg raise, while doing this, the therapist applied transverse pressure to the superior vertebrae at the affected spine level (L4 in case of L4 for instance) away from the painful side. Participants were instructed to stop the leg movement at the point leg pain was provoked, easing back, and then maintaining the position for 3 seconds before returning to the starting position. This was progressed from 1x3 repetitions on the first occasion to 2-3x6-8 repetitions on the first occasion with a rest interval of 30 seconds between sets. Progression was also achieved by applying pain-free overpressure to the range of straight leg raise.  *LVLA, concept not specified* | Sessions comprised neural mobilization, structured exercise, and TENS. A neural slider mobilization was applied with the subject inside lying, rhythmical movement of hip and knee flexion followed by hip and knee extension was conducted for 5x30 seconds. All participants received 2x5-7 repetitions of lumbar spine ROM exercises in 4-point kneeling, comprising pelvic tilting and heel sitting (lumbar flexion). Participants also received 4 pole low-intensity TENS (80-100Hz, wave duration 50-100ms) with two electrodes applied on the lumbar spine and 2 more on affected lower extremity for 30 minutes. Participants performed the exercise at home once per day 3x 10 repetitions with progressions as comfortable. | MT (of unclear duration) was provided in addition to an exercise and home exercise program. | No serious AE. |
| Schulz, 2019 | Patients in the IG received the same advice to perform the described home exercise program. In addition, MT based on physical condition and tolerance was administered. Up to 4 min of adjunct therapies to facilitate SMT (light soft tissue massage, active and passive stretching, ischemic compression of tender points, ice and heat).  HVLA- and LVLA-techniques whenever possible. Patients could attend up to 20 sessions with 10-20 minutes per session in addition to the home exercise program.  *HVLA & LVLA, concept not specified* | Home exercise program with instructions for self-care with postural adjustments, physical therapies, medications, importance of movement, staying active, instructions in low load exercises with graded progressions, to be done at home to improve balance, coordination, strength, and endurance. In addition, it comprised stretching exercises (seated or standing lumbar flexion, full spine flexion/ extension, motion cycles, quadriceps stretch, hamstring stretch, hip stretch, head retraction, chest expansion). Muscle strength and endurance exercises, chair squats, abdominal curls, seated back extension (isometric or using resistance tubing seated upright rows (using resistance tubing), and push-ups. Balance exercises: standing knee lifts, standing straight-leg hip flexion and extension.  Treatment was tailored to the individual and progressed as fast as possible. Patients were instructed to perform this exercise program once per week (45-60 minutes). | MT was provided in addition to (10-20 minutes per session, up to 20 sessions) a home exercise program. | Six Serious AE were recorded and all were non-study related. One hospitalization for cholecystectomy, one death due to lung cancer in the home exercise group, one hospitalization for acute cardiac symptoms, one new diagnosis of prostate cancer, one injury attending a hockey game, one transient ischemic attack during follow-up in the intervention group. |
| Sharif-Waqas, 2023 | Patients received thoracic HVLA- and LVLA-techniques in the prone position applied to hypomobile spinal segments identified by posterior-anterior and transverse pressure. No further specification was made by the authors concerning frequency, intensity, location of the technique applied.  *HVLA & LVLA, concept not specified.* | Thoracic spinal muscle exercises with 3x10 repetitions and rest period of 1 minute between intervals. Patient performed these sitting, prone lying, medium resistance, and elastic band was used. Scapular retraction was performed sitting with elbows at 90° degrees bent with an elastic band, in prone lying a second exercise was performed with the arms flexed and extending the thoracic spine. The third exercise was in sitting for thoracic rotation with the elastic band around the arms. Fourth exercise was sitting or prone with trunk side flexion. Both group salsa received ergonomic advice for maintaining correct anatomical posture. | MT (of unclear duration) was provided in addition to a supervised exercise program. | AE were not reported. |
| Shenouda, 2014 | Patients received MWM mulligan techniques as in medial glide, lateral glide, rotational MWM, dorsal glide with active knee flexion with the patient lying prone, supine, and sitting depending on the individual technique. Patients also received the same treatments as the control group.  *Mulligan MWM* | All patients received shock-wave therapy in long sitting position on the affected knee, slightly flexed and the hip abducted. Each patient received 10.000 shocks, 2.000 shocks per session and 5 sessions, each 1 week apart (0.18 mJ/mm², energy level 2-4, pulse rate 160/min, 6Hz). Further all patients completed an exercise program with stretching the hamstrings, calf muscles and straight leg raises (3x10 repetitions) and 3 sub maximal isometric contractions in the quadriceps muscle. | MT (of unclear duration) was provided in addition to an exercise and stretching program and shockwave therapy. | AE were not reported. |
| Subhash, 2020 | Patients performed Mulligan MWM in weight bearing quadruped, the therapist applied a rotational input on the scapula while pulling it caudally, moving it towards the thoracic spine, the patient rocks back slowly resulting in a shoulder flexion without pain. 3x10 repetitions and 10 minutes of hot pack and the same treatment as the control group.  *Mulligan MWM* | Patient performed active assisted ROM exercises with a cane, pendulum exercises, corner push up, cross body adduction, strengthening exercises, internal and external rotation with 1kg weight inside lying, active pain free range of motion in scaption, chair press, push-ups, and upright rows. | MT (of unclear duration) was provided in addition to an exercise program. | AE were not reported. |
| Tauqeer, 2024 | Patients received LVLA Grade III-IV techniques, including arthro-kinematic movements for different sub-joints at the shoulder, such as the glenohumeral, scapulothoracic, sternoclavicular, and acromioclavicular joints and cervical spine alongside soft tissue techniques and contract relax techniques. MT was individualized according to each patient’s needs; the progression was dependent on the assessment at the time of each MT session. It also included glenohumeral mobilization (anterior, posterior, inferior glide) and long-axis distraction Grade I-IV depending on irritability. A rate of one mobilization every one to two seconds, with 5x 30 seconds with 30-second rest in between was performed. Patients also performed the same exercise program as the control group.  *Maitland LVLA* | The CG was given only strengthening and stretching exercises for the involved and uninvolved side for 25-30 minutes under supervision. Upper trapezius, pectoralis minor, posterior shoulder was targeted for stretching and strengthening exercises. Patients were instructed to perform maximum external rotation, shoulder extension in prone position, wall-push ups for strength (5x3 repetitions initially, progressed to 5x5 repetitions). The stretches were 3x7 repetitions with 30 seconds break in between and increased 10x3 with stretches for pectoralis minor, upper trapezius and posterior shoulder. | MT (45 minutes) was provided in addition to an exercise and stretching program (25-30 minutes). | AE were not reported. |
| Tuncer, 2013 | Patients received soft tissue mobilization with intra- and extra-oral deep friction massage of painful muscles, TMJ mobilization (LVLA-technique) with caudal, ventro-caudal traction and ventral and mediolateral translation, TMJ stabilization (gentle isometric tension exercises against resistance, coordination exercises (guided opening and closing jaw movements) and post-isometric relaxation and stretching techniques for the masticatory and neck muscles. Patients also received the same routine care as the control group.  *Kaltenborn LVLA.* | Patients received education on pain, ergonomic advice, breathing exercises, relaxation techniques, posture correction exercises, mandibular exercises such as active and repetitive assisted muscle stretching, mouth opening and closing, medial and lateral gliding and resistance exercises. The authors made no further specification on dosage, frequency, intensity. | MT (of unclear duration) was provided in addition to education, advice, and a home exercise program | No serious AE. |
| Uhgreja, 2017 | Patients received MWM Mulligan techniques non-weight bearing (supine) and weight-bearing (on leg on a stool) to their affected knee with medial glide, medial rotation to the tibia while extending and bending the knee. Patients were instructed to perform 2x10 repetitions first non-weight bearing and progress to weight-bearing position depending on the irritability and to include it in their daily life. In total 4x10 repetitions per session were given, and patients instructed to walk a few steps. Afterwards patients received the same care the CG received.  *Mulligan MWM* | Short-wave diathermy for 10 minutes and exercises. Strengthening exercises consisted of static quadriceps exercise in supported long sitting, vastus medialis obliquus exercises in supine, resisted quadriceps exercise in sitting position with weight cuff around the ankle or manual resistance, hamstring flexion in prone against ankle weight or manual resistance, hip abduction while lying on the side. These were done isometrically for 10x10 seconds with 3 seconds break in between. Patients also performed stretching exercises with calf stretching in supine and hamstring stretching in supine with assistance from the therapist (3x30 seconds). Patients in both groups were advised to exercise at home as well. | MT (of unclear duration) was provided in addition to shortwave diathermy, an exercise, and a stretching program alongside advice to perform a home exercise. | AE were not reported. |
| UK Beam Team, 2004 | Patients in the IG attended eight sessions of HVLA over six weeks and eight sessions of exercise in the next six weeks with a refresher class at twelve weeks. The authors made no further specification upon dosage, frequency, intensity, techniques.  *HVLA, concept not specified.* | Patients participated in a group exercise program with groups of up to 10 people. The authors made no further specification of exercises, intensity, frequency, mode, rest, or any other training parameter. | MT (of unclear duration) was provided in addition to a group exercise program. | No serious AE. |
| Yang, 2015 | Patients received an upper thoracic lift manipulation (HVLA). While sitting with a posterior-anterior thrust direction along with horizontal adduction and expiration. The second possibility was a thoracic HVLA in supine where an anterior-to-posterior direction thrust is applied to T1-T4. No further specification concerning dosage, number of attempts or else was made. Patients also performed the same stability program as the control group.  *HVLA, concept not specified* | Patients performed static stability training and dynamic stability training for muscle endurance and power with a biofeedback unit for activating deep muscles like the longus colli and longus capitis. In dynamic stability, training subjects maintained cervical flexion and active assistive and active methods. After it was progressed to dynamic training through extremity movement while paying attention to cervical stability. Subjects were instructed to keep a target position 5x3 seconds with 15 seconds break in between. | MT (of unclear duration) was provided in addition to an exercise program with biofeedback. | AE were not reported. |
| Yiasemides, 2011 | In addition to the same advice and exercise, participants in the IG also received LVLA techniques to the shoulder, sternoclavicular and acromioclavicular joints in a passively sustained or oscillatory manner. Each technique was individualized from an intensity, directional and technique point of view. A minimum of 60% of all treatments to participants in the experimental group must involve passive shoulder LVLA to ensure an adequate dosage in this group.  *Maitland LVLA* | All participants received advice and exercises. Advice was on how to avoid painful movements during daily living, how to maintain a normal scapulohumeral rhythm, using the affected upper limb in a slow and careful manner. Exercises involved all shoulder muscle force couples (flexion, abduction, isolated rotation exercises, scapular depression exercises). The exercises were performed in a pain-free manner and tailored to the individual and as a daily home-based program and reviewed by the treating therapist 1-2 times per week. | MT (of unclear duration) was provided in addition to an exercise and home exercise program. | No serious AE. |
| AE, adverse event; CG, control group; IG, intervention group; HVLA, high velocity low-amplitude technique (manipulation); LVLA, low velocity low-amplitude technique (mobilization); MT, manual therapy; MWM, mobilization with movement (Mulligan); NAGs, natural apophyseal glide technique (Mulligan); SNAGs, sustained natural apophyseal glide technique (Mulligan); TENS, transcutaneous electrical nerve stimulation; | | | | |

## Assessment of clinical relevance

While there is ongoing debate and critique regarding how researchers assess statistical significance in relation to clinical relevance—particularly in determining the smallest worthwhile effect or minimal clinically important difference (Ferreira et al. 2012), —several studies have been published to guide clinicians and researchers on this topic. These studies primarily focus on low back pain (LBP), neck pain, and interventions such as physiotherapy and non-steroidal anti-inflammatory drugs. Based on this research, it is estimated that patients generally consider an improvement of 20-30% in pain intensity, function, or disability as meaningful (Christiansen et al. 2018; Fritsch et al. 2023; Hansford et al. 2023; Hansford et al. 2024). However, these effect sizes are specific to the intervention being studied.

Given the scarcity of evidence, we also included available thresholds from minimally clinically important difference (MCID) studies, despite the acknowledged limitations and ongoing criticisms—particularly the lack of patient involvement and the generic nature of these thresholds. The table below lists the questionnaires used in the included studies to assess function, pain, and disability, along with the thresholds applied to determine clinical relevance.

| **Supplementary Table 3**: Patient-reported outcome measures and the Minimally clinically important difference (MCID) | |
| --- | --- |
| Patient-reported Outcome Measure / Assessment tool | Minimally clinically relevant difference (MCID) |
| Visual analogue scale (VAS), numeric rating scale (NRS) | >1.5 points, >30% reduction for pain on the NRS (NRS, 1-10)  (Hansford et al. 2024; Fritsch et al. 2023; Christiansen et al. 2018) |
| Western Ontario and McMasters Universities Osteoarthritis Index (WOMAC) | >14.0 points  (Clement et al. 2018) |
| Neck Disability Index (NDI) | >7.5 points  (Young et al. 2009) |
| Oswestry Disability Index (ODI) | >12.8 points  (Copay et al. 2008) |
| Northwick Park Neck Pain Questionnaire (NPQ) | >3.0-5.0 points  (Sim et al. 2006) |
| Hip Disability and Osteoarthritis Outcome Score (HOOS) | >10.0 points  (Lyman et al. 2018) |
| Shoulder Pain and Disability Index (SPDI) | >14-20.0 points  (Dabija und Jain 2019) |
| Disability of the Arm, Shoulder, and Hand (DASH) | >8.0-13.0 points  (Dabija und Jain 2019) |
| Dutch Shoulder Disability Questionnaire (SDQ) | >14.0 points  (Dabija und Jain 2019) |
| Lower Extremity Functional Scale (LEFS) | >12.0 points  (McCormack et al. 2015) |
| Foot and Ankle Ability Measure (FAAM) | >9.0-77.0 points (not really applicable)  (Hung et al. 2019) |
| Tinnitus Handicap Inventory (THI) | >7.0 points  (Zeman et al. 2011) |
| Quick Disability of the Arm, Shoulder, and Hand (Quick-DASH) | >10.0 points  (Polson et al. 2010) |
| Short-Form-36 physcial component score (Quality of Life) | >1.8 units  (Fu et al. 2021) |
| Constant Murley Score | >10.0 points  (Kukkonen et al. 2013) |
| Roland Morris Disability Questionnaire (RMDQ) | >3.5 points  (Ostelo und Vet 2005) |
| Knee Injury and Osteoarthritis Outcome Score (KOOS) | >10.0 points  (Jacquet et al. 2021) |
| Ankle Joint Functional Assessment Tool (AJFAT) | Not available  (Eechaute et al. 2007) |

Literaturverzeichnis

Christiansen, David H.; Vos Andersen, Nils-Bo de; Poulsen, Per H.; Ostelo, Raymond W. (2018): The smallest worthwhile effect of primary care physiotherapy did not differ across musculoskeletal pain sites. In: *Journal of Clinical Epidemiology* 101, S. 44–52. DOI: 10.1016/j.jclinepi.2018.05.019.

Clement, Nicholas D.; Bardgett, Michelle; Weir, David; Holland, James; Gerrand, Craig; Deehan, David J. (2018): What is the Minimum Clinically Important Difference for the WOMAC Index After TKA? In: *Clinical orthopaedics and related research* 476 (10), S. 2005–2014. DOI: 10.1097/CORR.0000000000000444.

Copay, Anne G.; Glassman, Steven D.; Subach, Brian R.; Berven, Sigurd; Schuler, Thomas C.; Carreon, Leah Y. (2008): Minimum clinically important difference in lumbar spine surgery patients: a choice of methods using the Oswestry Disability Index, Medical Outcomes Study questionnaire Short Form 36, and pain scales. In: *The Spine Journal* 8 (6), S. 968–974. DOI: 10.1016/j.spinee.2007.11.006.

Dabija, Dominique I.; Jain, Nitin B. (2019): Minimal Clinically Important Difference of Shoulder Outcome Measures and Diagnoses: A Systematic Review. In: *American journal of physical medicine & rehabilitation* 98 (8), S. 671–676. DOI: 10.1097/PHM.0000000000001169.

Eechaute, Christophe; Vaes, Peter; van Aerschot, Lieve; Asman, Sara; Duquet, William (2007): The clinimetric qualities of patient-assessed instruments for measuring chronic ankle instability: a systematic review. In: *BMC Musculoskelet Disord* 8, S. 6. DOI: 10.1186/1471-2474-8-6.

Ferreira, Manuela L.; Herbert, Robert D.; Ferreira, Paulo H.; Latimer, Jane; Ostelo, Raymond W.; Nascimento, Dafne P.; Smeets, Rob J. (2012): A critical review of methods used to determine the smallest worthwhile effect of interventions for low back pain. In: *Journal of Clinical Epidemiology* 65 (3), S. 253–261. DOI: 10.1016/j.jclinepi.2011.06.018.

Fritsch, Carolina Gassen; Ferreira, Paulo H.; Lung, Thomas; McLachlan, Andrew J.; Ferreira, Manuela L. (2023): The smallest worthwhile change on function from a self-management intervention for non-persistent low back pain. In: *European spine journal : official publication of the European Spine Society, the European Spinal Deformity Society, and the European Section of the Cervical Spine Research Society*. DOI: 10.1007/s00586-023-07633-4.

Fu, Vivian; Weatherall, Mark; McNaughton, Harry (2021): Estimating the minimal clinically important difference for the Physical Component Summary of the Short Form 36 for patients with stroke. In: *The Journal of international medical research* 49 (12), 3000605211067902. DOI: 10.1177/03000605211067902.

Hansford, Harrison J.; Jones, Matthew D.; Cashin, Aidan G.; Ostelo, Raymond Wjg; Chiarotto, Alessandro; Williams, Sam A. et al. (2023): The smallest worthwhile effect on pain intensity of nonsteroidal anti-inflammatory drugs and exercise therapy for acute and chronic low back pain: a benefit-harm trade-off study. In: *Journal of physiotherapy*. DOI: 10.1016/j.jphys.2023.08.006.

Hansford, Harrison J.; Jones, Matthew D.; Cashin, Aidan G.; Ostelo, Raymond Wjg; Chiarotto, Alessandro; Williams, Sam A. et al. (2024): The smallest worthwhile effect on pain intensity of exercise therapy for people with chronic low back pain: a discrete choice experiment study. In: *J Orthop Sports Phys Ther*, S. 1–26. DOI: 10.2519/jospt.2024.12279.

Hung, Man; Baumhauer, Judith F.; Licari, Frank W.; Voss, Maren W.; Bounsanga, Jerry; Saltzman, Charles L. (2019): PROMIS and FAAM Minimal Clinically Important Differences in Foot and Ankle Orthopedics. In: *Foot & ankle international* 40 (1), S. 65–73. DOI: 10.1177/1071100718800304.

Jacquet, Christophe; Pioger, Charles; Khakha, Raghbir; Steltzlen, Camille; Kley, Kristian; Pujol, Nicolas; Ollivier, Matthieu (2021): Evaluation of the "Minimal Clinically Important Difference" (MCID) of the KOOS, KSS and SF-12 scores after open-wedge high tibial osteotomy. In: *Knee Surgery, Sports Traumatology, Arthroscopy* 29 (3), S. 820–826. DOI: 10.1007/s00167-020-06026-0.

Kukkonen, Juha; Kauko, Tommi; Vahlberg, Tero; Joukainen, Antti; Aärimaa, Ville (2013): Investigating minimal clinically important difference for Constant score in patients undergoing rotator cuff surgery. In: *Journal of shoulder and elbow surgery* 22 (12), S. 1650–1655. DOI: 10.1016/j.jse.2013.05.002.

Lyman, Stephen; Lee, Yuo-Yu; McLawhorn, Alexander S.; Islam, Wasif; MacLean, Catherine H. (2018): What Are the Minimal and Substantial Improvements in the HOOS and KOOS and JR Versions After Total Joint Replacement? In: *Clinical orthopaedics and related research* 476 (12), S. 2432–2441. DOI: 10.1097/corr.0000000000000456.

McCormack, Joshua; Underwood, Frank; Slaven, Emily; Cappaert, Thomas (2015): The Minimum Clinically Important Difference on the VISA-A and LEFS for Patients with Insertional Achilles Tendinopathy. In: *International Journal of Sports Physical Therapy* 10 (5), S. 639–644.

Ostelo, Raymond W. J. G.; Vet, Henrica C. W. de (2005): Clinically important outcomes in low back pain. In: *Best practice & research. Clinical rheumatology* 19 (4), S. 593–607. DOI: 10.1016/j.berh.2005.03.003.

Polson, Kate; Reid, Duncan; McNair, Peter J.; Larmer, Peter (2010): Responsiveness, minimal importance difference and minimal detectable change scores of the shortened disability arm shoulder hand (QuickDASH) questionnaire. In: *Manual therapy* 15 (4), S. 404–407. DOI: 10.1016/j.math.2010.03.008.

Sim, Julius; Jordan, Kelvin; Lewis, Martyn; Hill, Jonathan; Hay, Elaine M.; Dziedzic, Krysia (2006): Sensitivity to change and internal consistency of the Northwick Park Neck Pain Questionnaire and derivation of a minimal clinically important difference. In: *The Clinical journal of pain* 22 (9), S. 820–826. DOI: 10.1097/01.ajp.0000210937.58439.39.

Young, Brian A.; Walker, Michael J.; Strunce, Joseph B.; Boyles, Robert E.; Whitman, Julie M.; Childs, John D. (2009): Responsiveness of the Neck Disability Index in patients with mechanical neck disorders. In: *The spine journal : official journal of the North American Spine Society* 9 (10), S. 802–808. DOI: 10.1016/j.spinee.2009.06.002.

Zeman, Florian; Koller, Michael; Figueiredo, Ricardo; Aazevedo, Andreia; Rates, Marcello; Coelho, Claudia et al. (2011): Tinnitus handicap inventory for evaluating treatment effects: which changes are clinically relevant? In: *Otolaryngology--head and neck surgery : official journal of American Academy of Otolaryngology-Head and Neck Surgery* 145 (2), S. 282–287. DOI: 10.1177/0194599811403882.
